# Effects of high-intensity interval training on body composition, cardiorespiratory fitness, and selected lipid outcomes in adult women with overweight or obesity: a systematic review and meta-analysis

**DOI:** 10.3389/fphys.2026.1904753

**Published:** 2026-09-22

**Authors:** Yulong Wang, Qianyin Tan, Jingyou Zhong, Caixia Zhang, Yanhao Wang, Wei Gao, Ming Li

**Affiliations:** 1 School of Physical Education and Sports Science, Fujian Normal University, Fuzhou, Fujian, China; 2 Anglo-Chinese College, Fuzhou, Fujian, China; 3 School of Physical Education, Huaibei Normal University, Huaibei, Anhui, China

**Keywords:** body composition, cardiorespiratory fitness, high-intensity interval training, lipid outcomes, overweight or obesity, systematic review and meta-analysis, women

## Abstract

**Objective:**

This systematic review and meta-analysis aimed to evaluate the effects of high-intensity interval training (HIIT) on body composition, cardiorespiratory fitness, and selected lipid outcomes in adult women with overweight, obesity, or normal-weight obesity.

**Methods:**

This review followed the PRISMA guidelines and was registered in PROSPERO (CRD420261394616). PubMed, Web of Science Core Collection, Embase, and MEDLINE via Ovid were searched from database inception to 26 July 2026. Eligible studies were randomized controlled trials involving adult women with overweight, obesity, or normal-weight obesity and comparing HIIT with control conditions without structured exercise. Outcomes included body weight (BW), body mass index (BMI), body fat percentage (BF%), fat mass (FM), maximal or peak oxygen uptake (VO_2_max/VO_2_peak), high-density lipoprotein cholesterol (HDL-C), low-density lipoprotein cholesterol (LDL-C), total cholesterol (TC), and triglycerides (TG). Hedges’ g with 95% confidence intervals (CIs) was used as the pooled effect measure, and random-effects meta-analyses were performed.

**Results:**

Fifteen randomized controlled trials were included. Compared with controls without structured exercise, HIIT significantly reduced BW (g = −0.33, 95% CI −0.58 to −0.08; P = 0.01), BMI (g = −0.56, 95% CI −0.95 to −0.17; P< 0.01), BF% (g = −0.76, 95% CI −1.35 to −0.17; P = 0.01), and FM (g = −0.66, 95% CI −1.11 to −0.20; P< 0.01), and significantly improved VO_2_max/VO_2_peak (g = 1.09, 95% CI 0.70 to 1.47; P< 0.01). HIIT also increased HDL-C and reduced LDL-C, whereas effects on TC and TG were not statistically significant. Subgroup and meta-regression analyses were exploratory. Evidence certainty ranged from low to very low.

**Conclusions:**

Current randomized evidence suggests that, compared with controls without structured exercise, HIIT may favor adiposity-related body-composition outcomes and cardiorespiratory fitness in adult women with overweight, obesity, or normal-weight obesity, whereas its effects on selected lipid outcomes remain uncertain. The review did not systematically evaluate other cardiometabolic outcomes, including blood pressure, insulin resistance, glycemic control, or inflammatory markers. The findings should be interpreted cautiously because of the limited number of studies, small sample sizes, between-study heterogeneity, and low to very low certainty of evidence.

**Systematic Review Registration:**

https://www.crd.york.ac.uk/PROSPERO/, identifier CRD420261394616.

## 1 Introduction

Overweight and obesity represent one of the most pressing public health challenges worldwide. In 2022, approximately 2.5 billion adults were overweight, including more than 890 million living with obesity, and 43% of adults worldwide were classified as overweight, with the prevalence reaching 44% among women (NCD Risk Factor Collaboration (NCD-RisC), 2024). Moreover, the global age-standardized prevalence of obesity among adult women increased from 8.8% in 1990 to 18.5% in 2022, indicating a more than twofold increase over approximately three decades (NCD Risk Factor Collaboration (NCD-RisC), 2024). The associated health burden is also substantial, as higher-than-optimal body mass index was estimated to have contributed to approximately 3.7 million deaths from noncommunicable diseases in 2021 (GBD 2021 Risk Factors Collaborators, 2024). Overweight and obesity are not merely characterized by increased body weight but are also frequently accompanied by excessive adipose tissue accumulation, adverse alterations in body composition, and elevated cardiometabolic risk (Afshin et al., 2017; Caballero, 2019; Ross et al., 2020). In women, the obesity-related health burden has distinctive features. Influenced by factors such as age, estrogen status, menopausal stage, endocrine dysregulation, and sex-specific patterns of fat distribution, women may be particularly susceptible to increased body fat percentage, greater fat mass, and abnormalities in lipid metabolism, thereby further increasing the risk of cardiovascular disease, type 2 diabetes, and metabolic syndrome (Yusuf et al., 2005; Kodama et al., 2009; Jabbour and Iancu, 2021; Patten et al., 2023). Therefore, effective exercise interventions targeting women with overweight or obesity are of considerable importance for improving body composition and reducing cardiometabolic risk.

Body composition and cardiometabolic health indicators provide complementary insights into the health status of women with overweight or obesity. Body weight (BW) and body mass index (BMI) are commonly used indices for assessing overall adiposity; however, neither can distinguish changes in adipose tissue from those in lean or non-fat tissue compartments (Cornier et al., 2011; Nuttall, 2015). In contrast, body fat percentage (BF%) and fat mass (FM) more directly reflect changes in adipose tissue and are therefore particularly valuable for determining whether exercise interventions elicit meaningful fat-loss effects (Keating et al., 2017; Maillard et al., 2018). Beyond body composition, maximal or peak oxygen uptake (VO_2_max/VO_2_peak) is a key indicator of cardiorespiratory fitness and is closely associated with cardiovascular health and long-term disease risk (Lee et al., 2012; Milanović et al., 2015). In addition, lipid markers, including high-density lipoprotein cholesterol (HDL-C), low-density lipoprotein cholesterol (LDL-C), total cholesterol (TC), and triglycerides (TG), reflect lipid metabolic status and serve as important clinical indicators of cardiometabolic health (Cornelissen and Smart, 2013; Pescatello et al., 2015). Therefore, the combined assessment of body composition, cardiorespiratory fitness, and lipid profiles may provide a more comprehensive evaluation of the effects of exercise interventions on health outcomes in women with overweight or obesity.

Exercise intervention is a cornerstone non-pharmacological strategy for the management of overweight and obesity (Piercy et al., 2018). High-intensity interval training (HIIT) consists of repeated bouts of vigorous-intensity exercise interspersed with periods of recovery and is typically characterized by a relatively short training duration, a potent physiological stimulus, and the potential to improve cardiorespiratory fitness (Kessler et al., 2012; Tjønna et al., 2013; Keating et al., 2014; Weston et al., 2014; Cassidy et al., 2017; Kwok et al., 2022). From a mechanistic perspective, HIIT may exert favorable effects on body composition and cardiometabolic health through several pathways, including increased energy expenditure, enhanced fat oxidation, improved mitochondrial function, augmented cardiorespiratory fitness, and modulation of lipid metabolism (Talanian et al., 2007; Ramos et al., 2015; Batacan et al., 2017). For women with overweight or obesity, the time-efficient nature of HIIT may help address common barriers to traditional exercise interventions, such as insufficient time and poor adherence, and it is therefore considered a promising exercise strategy for this population (Kessler et al., 2012; Grossman et al., 2018; Batrakoulis et al., 2020).

However, the overall effects of HIIT on body composition and cardiometabolic health in women with overweight or obesity require further clarification. Although previous studies have suggested that HIIT may improve obesity-related health outcomes, study populations have often included general adults, mixed-sex samples, or male participants, and evidence specifically focused on women with overweight or obesity remains comparatively limited (Cocks et al., 2013; Dupuit et al., 2020; Danković et al., 2025). In addition, some studies have primarily focused on body weight, BMI, or single metabolic indicators, whereas comprehensive evaluations of multidimensional outcomes, including BF%, FM, VO_2_max/VO_2_peak, HDL-C, LDL-C, TC, and TG, remain insufficient (Way et al., 2019; Lopes et al., 2021). Moreover, differences across studies in age stage, baseline BMI, health status, intervention duration, training frequency, and exercise modality may influence the effects of HIIT and may also represent important sources of between-study heterogeneity (Zeng et al., 2021; Zhang et al., 2021; Wang et al., 2024).

On this basis, the present systematic review and meta-analysis aimed to quantitatively synthesize the effects of HIIT on three prespecified outcome domains in adult women with overweight, obesity, or normal-weight obesity: body composition, comprising BW, BMI, BF%, and FM; cardiorespiratory fitness, represented by VO_2_max/VO_2_peak; and selected lipid outcomes, comprising HDL-C, LDL-C, TC, and TG. Exploratory subgroup and meta-regression analyses were also conducted to examine whether selected participant and intervention characteristics might be associated with variation in the pooled effects. The review was not designed to evaluate the full spectrum of cardiometabolic health, including blood pressure, insulin resistance, glycemic control, or inflammatory markers. The findings were intended to characterize the currently available evidence and identify priorities for future intervention research rather than to define a definitive or optimal HIIT prescription.

## 2 Methods

### 2.1 Literature search

This review was conducted in accordance with the Preferred Reporting Items for Systematic Reviews and Meta-Analyses (PRISMA) guidelines (Page et al., 2021) and was prospectively registered in the PROSPERO database (registration number: CRD420261394616). PubMed, Web of Science Core Collection, Embase, and MEDLINE via Ovid were searched from database inception to 26 July 2026. These information sources were selected *a priori* to provide complementary coverage of biomedical, clinical, and multidisciplinary literature. PubMed and MEDLINE via Ovid were both retained because they were specified in the original search plan and provide different search interfaces for implementing controlled-vocabulary and free-text strategies, although substantial overlap between the two sources was expected and was addressed through systematic deduplication. Embase was included to broaden international biomedical coverage and to enable searching using Emtree indexing, whereas Web of Science Core Collection was included to provide multidisciplinary and citation-indexed coverage. The Cochrane Library, including the Cochrane Central Register of Controlled Trials, and Scopus were not included in the prespecified search plan. Their omission should not be interpreted as indicating that these sources were unnecessary; rather, it represents a limitation of the review and may have reduced the completeness of study retrieval.

The revised database-specific search strategies combined controlled vocabulary terms and free-text terms and were structured primarily around the target population, high-intensity interval training and related terminology, and randomized study design. Population-related terms included overweight or obesity and, where applicable, female-related terms. Outcome-related terms were removed from the revised search strategies to improve search sensitivity because eligible trials might not report all relevant outcomes in their titles, abstracts, keywords, or indexing terms. The exact search syntax was adapted according to the indexing system and search fields of each database.

No language or publication-status restrictions were applied during the electronic database searches. Ineligible publication types, including conference abstracts, narrative reviews, systematic reviews, meta-analyses, study protocols, commentaries, and case reports, were excluded during eligibility assessment according to the prespecified criteria. Trial registries and dedicated grey-literature databases were not systematically searched. However, the reference lists of included studies and relevant systematic reviews were manually examined to identify potentially eligible reports that might not have been captured by the electronic database searches. The complete search strategies and the numbers of records retrieved from each database are presented in **Supplementary Table 1**.

### 2.2 Selection process

All record s identified through the revised electronic database searches were imported into EndNote 20 for record management and deduplication. One reviewer (Y.-L.W.) initially identified bibliographic duplicates based on the title, author names, publication year, journal, digital object identifier (DOI), and other citation information. All records identified as potential duplicates were subsequently checked manually before removal. No automation tools were used to exclude records during title and abstract screening or full-text eligibility assessment.

After deduplication, the remaining records were provided to two reviewers (Q.-Y.T. and J.-Y.Z.), who independently screened the titles and abstracts against the prespecified eligibility criteria. Records that were clearly unrelated to the review question or did not meet the requirements regarding the study population, intervention, comparator, outcomes, or study design were excluded. Any disagreement between the two reviewers was resolved through discussion. If consensus could not be reached, a third reviewer (C.-X.Z.) independently assessed the record and made the final determination.

The full texts of all potentially eligible articles were subsequently assessed independently by the same two reviewers to determine final inclusion. At this stage, potential overlap between publications was also evaluated at the study level. To identify multiple publications originating from the same trial or participant cohort, the reviewers compared the author list, research institution and recruitment setting, participant recruitment period, trial registration number, ethics approval information, sample size, baseline participant characteristics, eligibility criteria, intervention and comparator protocols, study duration, assessment time points, and reported outcomes. Cross-references between potentially related publications were also examined.

Publications showing substantial concordance across these characteristics were considered multiple reports of the same underlying study rather than independent studies. To avoid double-counting participants, only one dataset from the same participant cohort was included for a given outcome and assessment time point. The report providing the most complete methodological information, the most comprehensive eligible outcome data, or the assessment time point most consistent with the prespecified review criteria was preferentially used. When companion publications from the same trial reported different eligible outcomes without duplicating participants within the same meta-analysis, the relevant data could be extracted from the respective reports, but the publications were linked and treated as reports of one underlying study. Any uncertainty regarding overlapping datasets was resolved through discussion, with adjudication by the third reviewer when necessary.

In addition to the revised electronic database searches, the reference lists of relevant systematic reviews and included studies were manually examined to identify potentially eligible reports that might not have been captured by the database searches. Records identified through this supplementary citation search were assessed using the same prespecified eligibility criteria and study-selection procedures. The number and disposition of records identified through this supplementary citation search are reported in Section 3.1 and presented separately in the PRISMA 2020 flow diagram (**Figure 1**).

**Figure 1:**
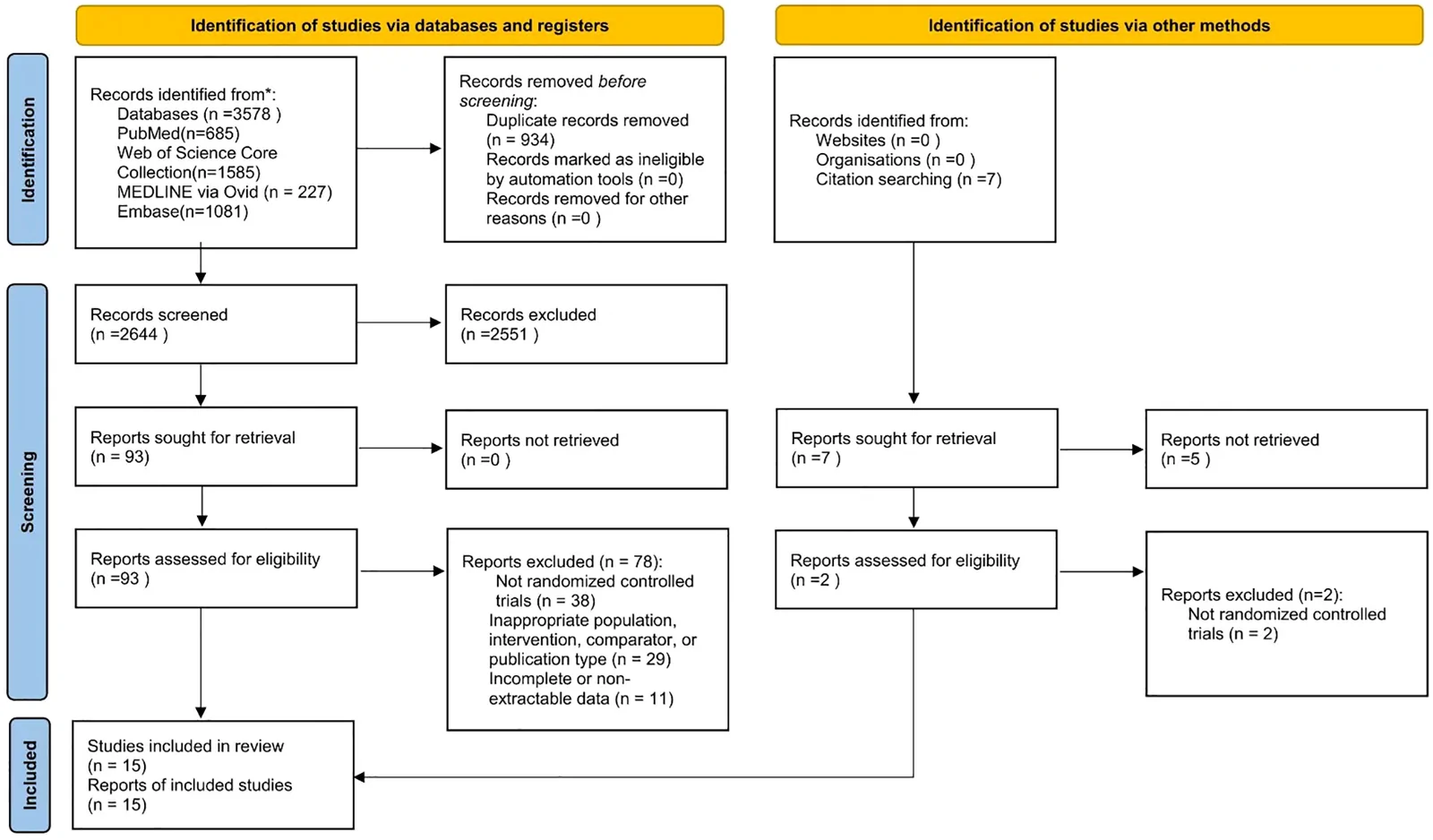
PRISMA 2020 flow diagram of the study selection process.

### 2.3 Eligibility criteria

#### 2.3.1 Inclusion criteria

Studies were included if they met the following criteria: (1) Participants were adult women aged ≥18 years with overweight, obesity, or normal-weight obesity. For studies using BMI-based definitions, overweight and obesity were defined according to the prespecified criteria reported in the original trials. Under the conventional adult BMI classification, overweight was defined as BMI ≥25.0 kg/m^2^ and obesity as BMI ≥30.0 kg/m^2^. Where an original study explicitly applied an ethnicity- or country-specific BMI threshold, the corresponding criterion was accepted. Studies using an adiposity-based definition were also eligible when the original trial explicitly identified excessive adiposity using a prespecified body fat percentage criterion. Normal-weight obesity was defined as a BMI within the normal range combined with a body fat percentage meeting the obesity threshold specified in the original study. Specifically, Hu et al. (2022) defined normal-weight obesity in women as a BMI of 18.5–24.9 kg/m^2^ combined with BF% >30%. Population eligibility was assessed from the prespecified criteria reported in the original studies and was not inferred solely from the group-level mean baseline BMI. Clinical conditions such as polycystic ovary syndrome, metabolic syndrome, postmenopausal status, hypertension, or a history of breast cancer were not, by themselves, considered sufficient evidence of overweight or obesity in the absence of an explicit adiposity criterion. When the available report did not permit conventional overweight or obesity eligibility to be verified for all retained participants, the study was classified as having unclear population eligibility and evaluated in a dedicated sensitivity analysis. Study-specific eligibility criteria and review-level decisions are presented in **Supplementary Table 9**. Studies enrolling both men and women were included only when data for eligible female participants could be extracted separately. Studies involving elite athletes, professional athletes, or competitive training populations were excluded from the scope of this review. (2) The experimental group received aerobic HIIT as the primary exercise intervention. Eligible HIIT protocols consisted of repeated bouts of vigorous- to near-maximal-intensity exercise interspersed with periods of active or passive recovery. Eligible exercise modalities included running, walking/running, cycling, circuit training, functional interval training, and other exercise protocols that clearly met the operational definition of aerobic HIIT adopted in this review. Protocols explicitly classified by the original investigators as sprint interval training (SIT), rather than HIIT or high-intensity training (HIT), were not included in the primary analysis. Eligible studies were required to report sufficient information on the intervention, including exercise intensity, interval and recovery structure, training frequency, and intervention duration. (3) The comparator was required not to include structured exercise and consisted of no exercise intervention, maintenance of usual lifestyle or habitual physical activity, usual care, health education, placebo, or another eligible non-exercise condition. To estimate the independent effect of HIIT, intervention arms involving an additional active co-intervention introduced as part of the trial protocol, such as an active nutritional supplement, were not included in the primary comparison. In factorial trials, the placebo-matched comparison or the comparison without an active co-intervention was used when it provided a direct comparison between HIIT and a control condition without structured exercise. Stable background care or treatment received equally by the intervention and comparator groups was permitted. Moderate-intensity continuous training and other structured exercise comparators were not included in the primary analyses.(4) Studies reported at least one of the following nine outcomes and provided extractable data suitable for meta-analysis. Body composition outcomes included BW, BMI, BF%, and FM. Cardiorespiratory fitness was represented by VO_2_max/VO_2_peak. Selected lipid outcomes included HDL-C, LDL-C, TC, and TG. (5) The study design was a randomized controlled trial (RCT).

#### 2.3.2 Exclusion criteria

Studies were excluded if they met any of the following criteria:(1) essential statistical information could not be obtained from the original publication, including the group-level post-intervention mean, standard deviation or standard error, and sample size, and the required data could not be derived using the prespecified statistical conversions described in Section 2.5; (2) the publication type was ineligible, including conference abstracts, narrative reviews, systematic reviews, meta-analyses, study protocols, commentaries, case reports, and animal studies; (3) the study population was inconsistent with the scope of this review, such as studies that did not enroll adult women or from which data for women with overweight or obesity could not be extracted separately; (4) the intervention was inconsistent with the research question, such as studies in which HIIT was not the primary intervention in the experimental group or in which the intervention protocol could not be clearly judged as meeting the defining characteristics of high-intensity interval training; (5) the comparator was ineligible, such as studies in which the control group also received HIIT or in which the independent effect of HIIT could not be clearly distinguished from that of the comparator intervention; (6) the study did not report any of the prespecified outcomes of interest, or reported relevant outcomes but did not provide extractable data; (7) the publication duplicated another report or reused participants from the same underlying trial. Multiple reports from the same trial were linked and treated as one study. For the same participant cohort, outcome, and assessment time point, only one dataset was included to avoid double-counting, according to the procedures described in the Selection Process section; and (8) the study was not a randomized controlled trial.

### 2.4 Data extraction

Data extraction was performed independently by two reviewers, and any discrepancies were checked and resolved through discussion. When agreement could not be reached, a third reviewer adjudicated. The extracted information included the first author, publication year, participant characteristics, sample sizes in the HIIT and comparator groups, participant sex, population characteristics, age, baseline BMI, exercise modality, training frequency, intervention duration, HIIT protocol, training intensity, comparator intervention, and outcome data. In addition, implementation, feasibility, and safety information was extracted when reported, including the number of randomized and completing participants, dropout numbers and reasons, intervention adherence or session attendance, exercise supervision, adverse events, prescribed training dose, and actual completed training dose. Prescribed training dose included exercise modality, intensity, interval and recovery structure, weekly frequency, session duration, intervention duration, and progression strategy. When an item was not reported in the original publication, it was recorded as not reported rather than inferred. Absence of adverse-event reporting was not interpreted as evidence that no adverse event occurred. Before the statistical analyses were conducted, a moderator-coding framework was prespecified in the data-extraction and analysis plan. The prespecified moderator domains included age stage, baseline BMI, intervention duration, training frequency, health status, and exercise modality. The corresponding categories were defined as follows: age<40 versus ≥40 years; baseline BMI<30 versus ≥30 kg/m^2^; intervention duration ≤8 versus >8 weeks; training frequency ≤3 versus >3 sessions per week; health status classified as general overweight/obesity or normal-weight obesity, polycystic ovary syndrome (PCOS), or comorbidities/special health conditions and exercise modality classified as running-/walking-based HIIT, cycling-based HIIT, or circuit-/functional-training-based HIIT. These moderator domains and coding categories were established before the outcome-specific subgroup and meta-regression analyses and were not selected according to the direction, magnitude, or statistical significance of the observed effect estimates.

The outcomes were categorized into three domains. Body composition outcomes included BW, BMI, BF%, and FM. Cardiorespiratory fitness was represented by VO_2_max/VO_2_peak. Selected lipid outcomes included HDL-C, LDL-C, TC, and TG.

For continuous outcomes, post-intervention group means, measures of dispersion, and sample sizes were used in the primary meta-analyses. Post-intervention values were selected because they were the most consistently reported data format across the included trials, whereas change scores and baseline-adjusted between-group estimates were not reported in a sufficiently consistent format to support a common analytical approach. To avoid combining effect estimates derived from different statistical frameworks, change-score estimates and adjusted estimates were not pooled together with unadjusted post-intervention values.

When baseline outcome data were available, the baseline means and measures of dispersion for the HIIT and comparator groups were also extracted and examined as described below. No numerical outcome included in the meta-analysis was obtained through graphical digitization. When an original study reported a standard error rather than a standard deviation, or reported an outcome using a unit different from that selected for the present analysis, the data were converted according to the procedures described in Section 2.5.

Baseline comparability was assessed descriptively for each available outcome by examining the direction and magnitude of differences in baseline means between the HIIT and comparator groups. Baseline significance tests reported in the original trials were not used as the sole basis for judging comparability or determining study eligibility. Where a potentially important baseline imbalance was identified, it was recorded during data verification and considered when interpreting the corresponding study and pooled estimate. No study was included or excluded solely on the basis of whether a baseline difference reached statistical significance.

When an included study contained multiple intervention or comparator arms, each arm was assessed separately against the prespecified eligibility criteria. Arm-level selection followed an explicit hierarchy: (1) participants were required to meet the population eligibility criteria; (2) the intervention was required to meet the operational definition of aerobic HIIT adopted in this review; (3) the comparator was required not to receive structured exercise; (4) the study arm was required to report an eligible outcome at the prespecified post-intervention assessment point; and (5) the comparison was required to permit the independent effect of HIIT to be reasonably isolated without an unmatched active co-intervention.

Arm selection was therefore based on these prespecified methodological criteria rather than on a subjective judgement regarding which arm appeared most closely aligned with the research question. The direction, magnitude, or statistical significance of the reported results was not considered during arm selection. Protocols explicitly classified by the original investigators as sprint interval training rather than HIIT or HIT were not included in the primary analysis. Similarly, an intervention arm involving an additional active co-intervention introduced as part of the trial protocol was excluded because it did not permit the independent effect of HIIT to be isolated. In factorial trials, the placebo-matched comparison or the comparison without an active co-intervention was selected when it provided an eligible direct comparison between HIIT and a control condition without structured exercise.

Accordingly, for Smith-Ryan et al. (2016), the 2MIN/HIT arm was compared with the non-exercise control arm, whereas the 1MIN arm was excluded because it was classified as SIT in the original report. For Sanayei et al. (2022), data were extracted from the HIIT-plus-placebo arm and the placebo-only arm because placebo administration was matched between groups and the principal between-group difference was the HIIT intervention. The HIIT-plus-*Chlorella vulgaris* and *Chlorella vulgaris*-only arms were not included because they involved an active nutritional co-intervention.

Within each outcome-specific meta-analysis, no participant group, including a shared comparator group, was entered more than once. Application of the prespecified arm-level eligibility criteria resulted in the retention of only one eligible HIIT-versus-comparator comparison from each included multi-arm trial. When a trial enrolled participants spanning different body-weight or adiposity categories, subgroup data were extracted separately only when the original publication reported those data separately. No subgroup was reconstructed from a study-level mean BMI or other aggregate characteristics. Trial-level population eligibility and data-extraction decisions are summarized in **Supplementary Table 9**.

All arm-level eligibility decisions were independently checked by two reviewers, and any disagreement was resolved through discussion, with adjudication by a third reviewer when necessary. The eligibility decision and corresponding rationale for each arm of the included multi-arm trials are presented in **Supplementary Table 2**. All extracted and converted data were independently cross-checked by two reviewers before inclusion in the meta-analysis.

### 2.5 Data conversion and unit standardization

All statistical and unit conversions were performed using Microsoft Excel (Microsoft Corporation, Redmond, WA, USA) and were independently cross-checked by two reviewers. Standard deviations were preferentially extracted directly from numerical tables or text in the original reports. When an original study reported a standard error (SE) rather than a standard deviation (SD), the reported mean was retained and the SD was calculated separately for each intervention or comparator group using the following equation:


$$SD=SE\times \sqrt{n}$$

where SD represents the standard deviation, SE represents the standard error, and n represents the sample size of the corresponding intervention or comparator group. No graph-digitization software was used because none of the numerical outcomes included in the meta-analysis required extraction from figures.

Blood lipid outcomes were standardized to mmol/L before meta-analysis. For total cholesterol (TC), high-density lipoprotein cholesterol (HDL-C), and low-density lipoprotein cholesterol (LDL-C), values reported in mg/dL were converted using the following equations:


$$Mean_{mmol/L}=\frac{Mean_{mg/dL}}{38.67}$$

$$SD_{mmol/L}=\frac{SD_{mg/dL}}{38.67}$$

For triglycerides (TG), values reported in mg/dL were converted using the following equations:


$$Mean_{mmol/L}=\frac{Mean_{mg/dL}}{88.57}$$

$$SD_{mmol/L}=\frac{SD_{mg/dL}}{88.57}$$

The same conversion factor was applied to both the mean and SD because both statistics were expressed on the same measurement scale. No missing SD was imputed using the SD from another study, a pooled SD across studies, or an assumed pre–post correlation coefficient. No included outcome required estimation of the mean or SD from medians, interquartile ranges, ranges, or confidence intervals. When neither an SD nor an SE was reported and the required dispersion measure could not be derived using the prespecified conversion procedures, the corresponding outcome was considered non-extractable and was not included in the meta-analysis.

### 2.6 Risk-of-bias assessment

Risk of bias in the included randomized controlled trials was reassessed using the revised Cochrane Risk of Bias tool for randomized trials (RoB 2; version 22 August 2019) (Higgins et al., 2024). The assessment focused on the effect of assignment to intervention, regardless of adherence to the assigned intervention.

The assessment covered the following five RoB 2 domains: (1) bias arising from the randomization process; (2) bias due to deviations from intended interventions; (3) bias due to missing outcome data; (4) bias in measurement of the outcome; and (5) bias in selection of the reported result. The signaling questions and decision algorithms provided in the RoB 2 guidance were used to inform the domain-level and overall judgements. Each domain and the overall assessment were classified as low risk of bias, some concerns, or high risk of bias, and a written justification was recorded for each judgement.

Because several included trials contributed multiple closely related outcomes, the potential for bias was considered in relation to the outcomes contributed by each study, while one conservative summary row per study was retained for presentation in the main risk-of-bias figures. In particular, potential differences across body-composition outcomes, cardiorespiratory-fitness outcomes, and lipid outcomes were considered when judging missing outcome data, outcome measurement, and selection of the reported result. When the judgement for a domain differed across eligible outcomes within the same study, the less favorable judgement was carried forward to the study-level summary. The outcomes contributed by each study and the supporting reasons for all domain-level judgements are reported in **Supplementary Table 5**.

For Domain 1, information regarding random sequence generation, allocation concealment, and any baseline differences suggesting a potential problem with the randomization process was examined. A study was not judged to be at low risk solely because it was described as randomized when the sequence-generation or allocation-concealment procedures were insufficiently reported.

For Domain 2, we considered whether deviations from the intended interventions arose because of the trial context, whether relevant co-interventions were balanced between groups, whether intervention adherence or exclusions from the analysis could have affected the comparison, and whether the analytical approach was appropriate for estimating the effect of assignment to intervention. Because blinding of participants and exercise personnel is generally difficult in exercise-training trials, lack of blinding alone was not considered sufficient for a high-risk judgement. Instead, the potential consequences of knowledge of intervention assignment were evaluated.

For Domain 3, missing outcome data were evaluated by considering the amount of missing data, reasons for missingness, between-group balance, the statistical method used to address missing data, and whether missingness could have depended on the true outcome value. These considerations were applied to the outcomes actually contributed by each study.

For Domain 4, the objectivity and consistency of the outcome-measurement procedures, assessor knowledge of intervention assignment, and the likelihood that such knowledge could influence the measured result were considered. Standardized anthropometric and laboratory measurements were distinguished from outcomes that could be more susceptible to participant effort, test termination, or operator judgement, including VO_2_max/VO_2_peak testing and some body-composition measurements.

For Domain 5, available trial registrations, study protocols, prespecified outcomes, measurement time points, and analytical methods were compared with the reported results. Absence of a prospective protocol or sufficiently detailed analysis plan was treated as a source of uncertainty rather than automatically as evidence of high risk.

Two reviewers independently completed the RoB 2 assessments. Disagreements were resolved through discussion, and a third reviewer adjudicated when consensus could not be reached. The overall judgement was classified as high risk when at least one domain was judged to be at high risk; as some concerns when no domain was at high risk but at least one domain raised some concerns; and as low risk when all five domains were judged to be at low risk. The assessments were conducted using the official RoB 2 framework, and the risk-of-bias visualizations were generated using R version 4.5.1.

### 2.7 Statistical analysis

Statistical analyses were conducted using Stata version 18.0. All outcomes included in the meta-analysis were continuous variables. The primary meta-analyses used the post-intervention sample size, mean, and standard deviation (SD) of the HIIT and comparator groups. Post-intervention values were selected because they were the most consistently reported data format across the included trials, whereas change scores and baseline-adjusted between-group estimates were not reported consistently enough to support comparable outcome-specific syntheses. To maintain a consistent analytical approach, change-score and baseline-adjusted estimates were not pooled with unadjusted post-intervention values. Baseline comparability was assessed descriptively as described in Section 2.4, and data conversion and unit standardization followed the procedures described in Section 2.5.

For each prespecified outcome, Hedges’ g and its corresponding 95% confidence interval (CI) were calculated. Each outcome was synthesized separately, and effect estimates from different outcome domains were not combined into a single pooled estimate. The use of a standardized effect measure was prespecified to maintain a consistent effect-size calculation and reporting framework across the nine outcome-specific syntheses; no direct comparison of Hedges’ g magnitudes across different outcome domains was intended. Because several comparisons had small group sample sizes, with HIIT-group sample sizes ranging from 8 to 30 participants, Hedges’ g was used to reduce the small-sample bias of the uncorrected standardized mean difference (Lin and Aloe, 2021). The pooled estimates therefore represent standardized between-group differences rather than absolute changes expressed in natural units. For BW, BMI, BF%, FM, LDL-C, TC, and TG, Hedges’ g< 0 indicated a greater reduction in the HIIT group than in the comparator group. For VO_2_max/VO_2_peak and HDL-C, Hedges’ g > 0 indicated a more favorable effect of HIIT.

Given the anticipated clinical and methodological diversity across the included studies, all primary analyses were conducted using random-effects models. This choice was based on expected variation in the underlying intervention effects and was not determined solely by the statistical significance of the heterogeneity test. Between-study heterogeneity was assessed using Cochran’s Q test, the I^2^ statistic, and the between-study variance τ^2^. I^2^ values of approximately 25%, 50%, and 75% were used as broad reference points for low, moderate, and high heterogeneity, respectively (Higgins et al., 2003). These thresholds were not treated as rigid decision rules; I^2^ was interpreted together with the direction and dispersion of the study-specific estimates, the overlap of their CIs, and the magnitude of τ^2^. Between-study variance was estimated using the restricted maximum likelihood (REML) method (Veroniki et al., 2016).

Potential sources of between-study heterogeneity were explored using subgroup analyses and univariable random-effects meta-regression. The moderator domains of age stage, baseline BMI, intervention duration, training frequency, health status, and exercise modality, together with their coding categories, were established in the review-level data-extraction and analysis plan before the outcome-specific analyses were conducted. They were not selected according to the direction, magnitude, or statistical significance of the observed results (Thompson and Higgins, 2002). Subgroup analyses were conducted when studies could be classified according to the prespecified categories. The numbers of independent effect estimates and participants contributing to each subgroup were reported as K and N, respectively, in the corresponding tables. Categories containing only one effect estimate were retained for transparent reporting but were interpreted descriptively and were not regarded as reliable evidence of effect modification. Interpretation focused primarily on the formal test for between-subgroup differences rather than on whether the pooled estimate within an individual subgroup reached statistical significance.

Univariable random-effects meta-regression was conducted for the study-level moderators of mean participant age, baseline BMI, intervention duration, and training frequency when sufficient between-study variation was available. Health status and exercise modality were examined using subgroup analyses because they were categorical variables and several categories were sparsely represented. No multivariable meta-regression model was fitted because the available number of independent effect estimates was insufficient to support reliable simultaneous estimation of multiple moderator effects. The univariable models included 8–12 independent effect estimates; models with fewer than 10 estimates were retained only for exploratory interpretation and were not used for confirmatory inference. Results were reported using the regression coefficient (β), 95% CI, P value, residual I^2^, residual τ^2^, and estimated R^2^ where available. Estimated R^2^ was interpreted descriptively as the reduction in the estimated between-study variance associated with the fitted model, rather than as a definitive proportion of heterogeneity causally explained. Because the analyses used aggregate study-level characteristics and were based on relatively few effect estimates, they had limited statistical power and were susceptible to ecological bias and confounding between study characteristics. Accordingly, subgroup and meta-regression findings were regarded as exploratory and hypothesis-generating rather than confirmatory or causal and were not used to define an optimal age-, BMI-, duration-, frequency-, health-status-, or modality-specific HIIT prescription (Thompson and Higgins, 2002).

Four sets of *post hoc* sensitivity analyses were conducted to assess the stability of the primary findings. First, all outcome-specific meta-analyses were repeated after excluding studies judged to be at overall high risk of bias according to RoB 2. Second, for BW, BMI, BF%, and FM, a population-definition sensitivity analysis was conducted after excluding Hu et al. (2022), which enrolled women with normal-weight obesity defined by a normal-range BMI combined with an elevated body fat percentage. This analysis assessed whether the findings remained consistent when the evidence was restricted to studies using conventional BMI-based definitions of overweight or obesity and did not imply that Hu et al. (2022) was ineligible for the primary analysis. Third, a separate population-eligibility sensitivity analysis excluded studies for which conventional overweight or obesity eligibility could not be clearly verified from the available report, according to the review-level classifications presented in **Supplementary Table 9**. Hu et al. (2022) was not included in this third category because the normal-weight-obesity phenotype was explicitly defined and was examined separately in the preceding analysis.

Fourth, leave-one-out sensitivity analyses were conducted for outcomes with I^2^ ≥50% in the primary analysis. Each study was sequentially omitted, and the pooled Hedges’ g, 95% CI, P value, I^2^, and τ^2^ were re-estimated to evaluate the influence of individual studies on the direction, magnitude, statistical interpretation, and heterogeneity of the pooled result (Viechtbauer and Cheung, 2010). Outcome-specific quantitative pooling was performed only when at least two studies remained after an exclusion. All sensitivity analyses used the same random-effects REML model as the corresponding primary analysis. No formal deletion threshold was applied, and no study was removed from the primary analysis solely because of a sensitivity-analysis finding. These analyses were interpreted as exploratory assessments of robustness.

Sensitivity analyses excluding active exercise comparators were not applicable because no moderate-intensity continuous training or other structured exercise comparator was included in the primary meta-analyses. Change-score sensitivity analyses were not feasible because change scores and their corresponding measures of dispersion were not reported consistently enough to support comparable outcome-specific analyses. No graphically digitized, imputed, or distributionally estimated means or SDs were used. As described in Section 2.5, the only transformations involved conversion of reported SEs to SDs using the corresponding sample sizes and standardization of blood-lipid units; these were prespecified algebraic conversions rather than imputations.

Potential small-study effects and funnel-plot asymmetry were explored through visual inspection of funnel plots and Egger’s regression test (Egger et al., 1997; Sterne et al., 2011). Egger’s test was formally interpreted only when at least 10 independent effect estimates were available for an outcome; results based on fewer than 10 estimates were reported descriptively because of limited statistical power (Sterne et al., 2011). Because the effect measure used in this review was a standardized mean difference, Egger’s test results were interpreted cautiously and were treated as exploratory evidence of small-study effects rather than as a definitive diagnosis of publication bias. Funnel-plot asymmetry was not considered synonymous with publication bias because it may also arise from between-study heterogeneity, methodological differences, chance, or other small-study effects. A non-significant Egger’s test was not interpreted as evidence that publication bias was absent. For pooled-effect estimates, meta-regression coefficients, and Egger’s regression tests, two-sided P< 0.05 was considered statistically significant. Between-subgroup differences were evaluated using the corresponding Q test.

The corresponding Egger’s regression plots for BW, BMI, BF%, FM, VO_2_max/VO_2_peak, HDL-C, LDL-C, TC, and TG are presented in **Supplementary Figures 1**-**9**, respectively. The corresponding regression-analysis plots for these outcomes are presented in **Supplementary Figures 10**-**18**, respectively.

Finally, the certainty of evidence for each prespecified outcome was assessed using the Grading of Recommendations Assessment, Development and Evaluation (GRADE) approach (Guyatt et al., 2008). Evidence from randomized controlled trials initially started at high certainty and was evaluated across five domains: risk of bias, inconsistency, indirectness, imprecision, and publication bias. Risk of bias was judged using the outcome-specific RoB 2 assessments and the contribution of studies rated as low risk, some concerns, or high risk. Inconsistency was evaluated using I^2^, τ^2^, the direction and dispersion of study-specific estimates, the overlap of CIs, and whether the observed heterogeneity could be explained with sufficient certainty. Indirectness was assessed by comparing the populations, interventions, comparators, and outcomes in the available evidence with the prespecified review question. Imprecision was judged according to the total information size, the width of the pooled 95% CI, whether the CI crossed the line of no effect, and whether it included materially different effect magnitudes or directions. Publication bias was evaluated by integrating the scope of the electronic database and supplementary citation searches, the number and size of the included studies, funnel-plot distributions, Egger’s test results when sufficiently interpretable, and available trial-registration or protocol information. Each downgrade decision was made separately for each outcome and was supported by an outcome-specific explanation in **Table 1**. The resulting certainty of evidence was categorized as high, moderate, low, or very low.

**Table 1:** GRADE assessment of the certainty of evidence.

Outcome	Participants (RCTs)	Certainty-of-evidence assessment					Hedges’ g (95% CI)	CIGRADE certainty of evidence and recommendation grade
		Risk of bias	Inconsistency	Indirectness	Imprecision	Publication bias		
Body weight	336 (11)	Serious^a^	Not serious^b^	Not serious^d^	Serious^e^	Not serious^g^	−0.33 (−0.58 to −0.08)	⊕⊕○○ Low
BMI	343 (11)	Serious^a^	Serious^c^	Not serious^d^	Serious^e^	Not serious^g^	−0.56 (−0.95 to −0.17)	⊕○○○ Very low
Body fat percentage	297 (10)	Serious^a^	Serious^c^	Not serious^d^	Serious^e^	Not serious^g^	−0.76 (−1.35 to −0.17)	⊕○○○ Very low
Fat mass	229 (8)	Serious^a^	Serious^c^	Not serious^d^	Serious^e^	Not serious^h^	−0.66 (−1.11 to −0.20)	⊕○○○ Very low
Maximal or peak oxygen uptake	370 (12)	Serious^a^	Serious^c^	Not serious^d^	Serious^e^	Not serious^g^	1.09 (0.70 to 1.47)	⊕○○○ Very low
High-density lipoprotein cholesterol	253 (8)	Serious^a^	Serious^c^	Not serious^d^	Serious^e^	Not serious^h^	0.84 (0.24 to 1.45)	⊕○○○ Very low
Low-density lipoprotein cholesterol	253 (8)	Serious^a^	Serious^c^	Not serious^d^	Serious^e^	Not serious^h^	−0.78 (−1.29 to −0.27)	⊕○○○ Very low
Total cholesterol	276 (9)	Serious^a^	Serious^c^	Not serious^d^	Very serious^f^	Not serious^h^	−0.35 (−0.70 to 0.01)	⊕○○○ Very low
Triglycerides	276 (9)	Serious^a^	Serious^c^	Not serious^d^	Very serious^f^	Not serious^h^	−0.24 (−0.69 to 0.21)	⊕○○○ Very low

RCT, randomized controlled trial; CI, confidence interval; GRADE, Grading of Recommendations Assessment, Development and Evaluation. Randomized controlled trial evidence started at high certainty and was rated down by one or two levels for serious or very serious concerns in five domains: risk of bias, inconsistency, indirectness, imprecision, and publication bias. GRADE categories were interpreted as follows: ⊕⊕⊕⊕, high certainty (the true effect is likely to be close to the estimate); ⊕⊕⊕○, moderate certainty (the true effect is likely to be close to the estimate but may be substantially different); ⊕⊕○○, low certainty (the true effect may be substantially different from the estimate); and ⊕○○○, very low certainty (the true effect is likely to be substantially different from the estimate). Hedges’ g< 0 indicates a greater reduction with HIIT for BW, BMI, BF%, FM, LDL-C, TC, and TG; Hedges’ g > 0 indicates a greater increase with HIIT for VO_2_max/VO_2_peak and HDL-C.^a^ Risk of bias: Downgraded by one level because the body of evidence for each outcome included trials with some concerns or high overall RoB 2 judgements, mainly related to the randomization process, missing outcome data, and selection of the reported result. The inability to blind participants and exercise personnel was not used alone as a reason for downgrading. ^b^ BW was not downgraded for inconsistency because heterogeneity was low (I^2^ = 24.23%) and study estimates were generally consistent in direction. ^c^ BMI, BF%, FM, VO_2_max/VO_2_peak, HDL-C, LDL-C, TC, and TG were downgraded by one level for inconsistency because moderate-to-substantial heterogeneity remained and was not explained with sufficient certainty. ^d^ No outcome was downgraded for indirectness because the populations, HIIT interventions, comparators without structured exercise, and measured outcomes were directly aligned with the review question. ^e^ BW, BMI, BF%, FM, VO_2_max/VO_2_peak, HDL-C, and LDL-C were downgraded by one level for imprecision because the total information size was limited and the 95% CIs included materially different magnitudes of effect. ^f^ TC and TG were downgraded by two levels for imprecision because the information size was limited and the 95% CIs crossed the line of no effect; the TG interval also included a possible effect in the opposite direction. ^g^ For outcomes with at least 10 studies, no downgrade was applied for publication bias because no marked funnel-plot asymmetry or statistically detectable small-study effect was identified; however, publication bias could not be excluded. ^h^ For outcomes with fewer than 10 studies, no downgrade was applied because no direct evidence strongly suggested publication bias; however, assessment was limited, and non-significant Egger tests were not interpreted as evidence that publication bias was absent.

## 3 Results

### 3.1 Literature search results

The revised electronic database searches identified 3,578 records, including 685 records from PubMed, 1,585 from Web of Science Core Collection, 1,081 from Embase, and 227 from MEDLINE via Ovid. After removal of 934 duplicate records, 2,644 records remained for title and abstract screening. A total of 2,551 records were excluded during title and abstract screening, and 93 reports were sought for retrieval. All 93 reports were successfully retrieved and assessed for eligibility. After full-text review, 78 reports were excluded: 38 were not randomized controlled trials, 29 had an ineligible population, intervention, comparator, or publication type, and 11 had incomplete or non-extractable data.

In addition, seven records were identified through supplementary citation searching. Full texts were sought for all seven reports; five could not be retrieved, and the remaining two reports were assessed for eligibility. Both reports were excluded because they were not randomized controlled trials. No additional eligible studies were identified through the supplementary citation search. Ultimately, 15 randomized controlled trials were included in the systematic review and meta-analysis (Almenning et al., 2015; Arad et al., 2015; Zhang et al., 2015; Smith-Ryan et al., 2016; Connolly et al., 2017; Ballesta-García et al., 2020; Benham et al., 2021; Samhan et al., 2021; Hu et al., 2022; Philbois et al., 2022; Sanayei et al., 2022; Guo et al., 2023; Kazemi et al., 2023; Mohammadi et al., 2023; Taha et al., 2023). The detailed study selection process is shown in **Figure 1**.

### 3.2 Study characteristics and risk-of-bias assessment

A total of 15 randomized controlled trials published between 2015 and 2023 were included in this review (Almenning et al., 2015; Arad et al., 2015; Zhang et al., 2015; Smith-Ryan et al., 2016; Connolly et al., 2017; Ballesta-García et al., 2020; Benham et al., 2021; Samhan et al., 2021; Hu et al., 2022; Philbois et al., 2022; Sanayei et al., 2022; Guo et al., 2023; Kazemi et al., 2023; Mohammadi et al., 2023; Taha et al., 2023). All participants were women, including women with general overweight or obesity, normal-weight obesity, polycystic ovary syndrome, abdominal obesity, postmenopausal obesity or metabolic syndrome, breast cancer survivors with overweight or obesity, and women with obesity and hypertension. Sample sizes in the HIIT groups ranged from 8 to 30 participants. The included studies showed some variability in participants’ health status, age stage, and baseline BMI. With respect to adiposity classification, the eligible populations included women with BMI-defined overweight or obesity and women with an explicitly defined adiposity-based obesity phenotype. Population eligibility was determined from the prespecified criteria reported in the original trials rather than solely from the group-level mean baseline BMI. In particular, Hu et al. (2022) enrolled women with normal-weight obesity. Although the mean baseline BMI in this study was 21.93 ± 0.28 kg/m^2^, participants were required to have a BMI of 18.5–24.9 kg/m^2^ together with BF% >30%. Therefore, this study was included on the basis of an explicitly defined body-fat-based obesity phenotype rather than BMI-defined overweight or obesity.

The HIIT interventions varied across the included studies and mainly comprised treadmill-based HIIT, interval running, cycle ergometer training, HIIT-based aerobic exercise, high-intensity circuit training, functional high-intensity interval training, resistance-based interval training, and outdoor running- or walking-based HIIT. Intervention duration ranged primarily from 3 to 18 weeks. Training frequency was most commonly three sessions per week, although a small number of studies implemented two, four, or five sessions per week. Training intensity was prescribed mainly according to maximal heart rate, peak heart rate, heart-rate reserve, maximal oxygen uptake, peak oxygen uptake, maximal aerobic speed, or ratings of perceived exertion. All included comparisons involved HIIT versus comparator groups that did not receive structured exercise. The comparator conditions included no training or no additional exercise, maintenance of usual lifestyle or habitual physical activity, health education, placebo, and usual care without structured exercise. In studies involving medication, dietary advice, placebo, or other background management, the comparison was included only when the independent effect of HIIT could be reasonably distinguished. No moderate-intensity continuous training or other active exercise comparator was included in the pooled analyses. Detailed comparator conditions are presented in **Table 2**.

**Table 2:** Characteristics of included studies.

Study	Sample Size					Intervention							Outcomes
	HIIT group, n	Sex	Population	Age, years	Baseline BMI, kg/m^2^	Exercise Modality	Frequency, sessions/week	Duration, weeks	HIIT Protocol	Intensity	Control group, n	Comparator Condition	
Zhang et al., 2015 (Zhang et al., 2015)	12	Women	Chinese female college students with overweight or obesity	21.0 ± 1.0	25.8 ± 2.7	Treadmill-based HIIT	4	12	Four 4-min running bouts interspersed with 3-min walking recovery; including a 10-min warm-up and a 5-min cool-down,	85–95% HRpeak; recovery at 50–60% HRpeak	11	No-training control; participants maintained their usual daily activities and dietary habits.	BW, BMI, BF%, FM, VO_2_max, TC, TG
Smith-Ryan et al., 2016 (Smith-Ryan et al., 2016)	10	Women	Sedentary women with overweight or obesity	33.6 ± 11.6	28.6 ± 3.7	Cycle ergometer	3	3	2MIN: 5 × 2-min high-intensity bouts.	2MIN: 80–100% VO_2_peak.	9	No-training control.	BF%, FM, VO_2_peak, HDL-C, LDL-C, TC, TG
Sanayei et al., 2022 (Sanayei et al., 2022)	11	Women	Women with overweight or obesity	29.09 ± 4.70	29.98 ± 2.90	Running-based HIIT	3	8	Progressed from 20 × 40-s bouts in week 1 to 12 × 20-s bouts in weeks 5–8; including warm-up and cool-down.	Week 1: 50–60% HRR; week 2: 60–70% HRR; week 3: 70–80% HRR	9	Placebo control; participants did not undertake HIIT.	BW, BF%, VO_2_max
Hu et al., 2022 (Hu et al., 2022)	17	Women	Chinese female college students with normal-weight obesity	19.20 ± 1.10	21.93 ± 0.28	Treadmill/comprehensive high-intensity exercise training	2	4	Three 9-min sets of high-intensity comprehensive exercises, with 1-min recovery between sets.	90% HRmax	13	Weekly health education without participation in exercise training.	BW, BMI, BF%, FM
Mohammadi et al., 2023 (Mohammadi et al., 2023)	14	Women	Women with PCOS	24.2 ± 4.8	29.52 ± 4.5	Interval running	3	8	30-s running bouts alternated with 30-s active recovery; 4–6 sets, each consisting of 4 laps, with 5-min recovery between sets.	100–110% maximal aerobic velocity	14	Participants maintained their usual lifestyle and performed no additional exercise.	BW, BMI, BF%, VO_2_max, HDL-C, LDL-C, TC, TG
Philbois et al., 2022 (Philbois et al., 2022)	25	Women	Women with PCOS	29 ± 4	27.8 ± 4.2	Treadmill-based HIIT	3	16	5-min warm-up, HIIT main session, and 5-min cool-down; 2-min high-intensity bouts alternated with 3-min low-intensity recovery.	High intensity at 85–90% HRR; low intensity at 65–70% HRR	25	No exercise training.	BW, BMI, VO_2_peak, HDL-C, LDL-C, TC, TG
Benham et al., 2021 (Benham et al., 2021)	13	Women	Women with PCOS	29.1 ± 4.1	31.4 ± 8.6	Self-selected aerobic instructor-led HIIT	3	24	Ten cycles of 30-s high-intensity exercise followed by 90-s low-intensity recovery; including a 5-min warm-up and a 5-min cool-down.	High intensity at 90% HRR or Borg 9/10; recovery consisted of low-intensity aerobic exercise	16	No-exercise control; participants maintained their habitual physical activity levels.	BW, BMI, VO_2_max, HDL-C, LDL-C, TC, TG
Almenning et al., 2015 (Almenning et al., 2015)	8	Women	Women with PCOS	27.2 ± 5.5	26.1 ± 6.5	Treadmill, outdoor walking or running, or cycling, selected by participants	3	10	Two weekly sessions of 4 × 4-min HIIT with 3-min recovery between bouts, plus one weekly session of 10 × 1-min HIIT.	4 × 4-min bouts at 90–95% HRmax, with recovery at approximately 70% HRmax; 10 × 1-min bouts performed at maximal intensity	9	No-training control.	BW, BMI, BF%, FM, VO_2_max, HDL-C, LDL-C, TC, TG
Guo et al., 2023 (Guo et al., 2023)	23	Women	Young female college students with overweight or obesity	20.00 ± 1.48	25.53 ± 2.59	Aerobic-exercise-based HIIT	3	4	Campus aerobic HIIT consisting of 8 movements × 4 sets, with 10-s passive rest between movements.	>85% HRmax	25	No exercise training; participants maintained their usual lifestyle.	BF%, VO_2_max
Kazemi et al., 2023 (Kazemi et al., 2023)	15	Women	Postmenopausal women with metabolic syndrome	45–65	28.33 ± 0.36	HIIT-based aerobic training	3	8	3-min high-intensity training alternated with 3-min moderate-intensity walking.	High intensity at 80–90% HRmax; recovery at 55–65% HRmax	15	Participants maintained their usual lifestyle and received no exercise-training intervention.	BW, BMI, FM, VO_2_peak, HDL-C, LDL-C TC TG
Taha et al., 2023 (Taha et al., 2023)	25	Women	Women with obesity and hypertension	48.32 ± 4.48	33.90 ± 2.58	Cycle ergometer	3	12	10-min warm-up followed by 4 × 4-min high-intensity cycling bouts, with 3-min active recovery between bouts.	85–90% HRpeak; recovery at 60–70% HRpeak	28	Participants continued their usual medications and dietary advice without taking part in supervised exercise training.	HDL-C, LDL-C, TC, TG
Samhan et al., 2021 (Samhan et al., 2021)	30	Women	Breast cancer survivors with overweight or obesity	49.7 ± 8.9	33.22 ± 1.29	Treadmill	3	8	5-min warm-up followed by 4 × 4-min high-intensity intervals, with 3-min active recovery between bouts.	75–90% HRmax; recovery at 50–60% HRmax	30	Usual care; participants maintained their daily activities and did not undertake structured exercise.	BW, BMI, BF%, FM, VO_2_peak
Connolly et al., 2017 (Connolly et al., 2017)	15	Women	Inactive premenopausal women	44 ± 7	25.3 ± 4.9	Self-paced cycle ergometer HIIT	3	12	Five 5-min training blocks, each consisting of 30 s at low intensity, 20 s at moderate intensity, and 10 s at high intensity.	Self-paced; high-intensity phase at approximately 94–95% HRpeak	15	Inactive control; participants maintained their usual daily lifestyle.	BW, BMI, BF%, FM, VO_2_peak, HDL-C, LDL-C, TC,TG
Arad et al., 2015 (Arad et al., 2015)	9	Women	African American women with overweight or obesity	29 ± 4	32.5 ± 3.6	Cycle ergometer HIIT	3	14	Approximately 24 min per session: 6-min warm-up followed by four high-intensity cycles.	Work intervals at 75–90% HRR; recovery at 50% HRR	11	Participants maintained their usual physical activity and followed dietary control to maintain a stable body weight.	BW, BMI, BF%, FM, VO_2_peak
Ballesta-García et al., 2020 (Ballesta-García et al., 2020)	17	Women	Middle-aged and older women	66.3 ± 5.4	30.4 ± 4.13	High-intensity interval circuit training (HICT)	2	18	Circuit-training format, approximately 1 h per session.	High-intensity circuit training	17	Non-exercise control group.	BMI

HIIT, high-intensity interval training; HIIC, high-intensity interval circuit; HICT, high-intensity circuit training; BMI, body mass index; BW, body weight; BF%, body fat percentage; FM, fat mass; VO_2_max, maximal oxygen uptake; VO_2_peak, peak oxygen uptake; HDL-C, high-density lipoprotein cholesterol; LDL-C, low-density lipoprotein cholesterol; TC, total cholesterol; TG, triglycerides; HRpeak, peak heart rate; HRmax, maximal heart rate; HRR, heart-rate reserve; MAV, maximal aerobic velocity; Borg, Borg rating of perceived exertion. Group sample sizes refer to the post-intervention or evaluable participants reported by the original studies; detailed randomized and completed sample sizes, attrition, and reasons for withdrawal are provided in **Supplementary Table 6**. Age and baseline BMI were extracted as reported in the original studies and are presented primarily as mean ± standard deviation or as ranges. Training frequency is expressed as sessions per week, and intervention duration is expressed in weeks. Outcomes refer to body composition and cardiometabolic health indicators reported in the included studies and available for data extraction. In Hu et al. (2022), normal-weight obesity was defined as a BMI of 18.5–24.9 kg/m^2^ combined with BF% >30% in women; therefore, the reported mean BMI of 21.93 ± 0.28 kg/m^2^ was within the expected normal-BMI range for this adiposity-defined phenotype. Reference numbers in the table (Almenning et al., 2015; Arad et al., 2015; Zhang et al., 2015; Smith-Ryan et al., 2016; Connolly et al., 2017; Ballesta-García et al., 2020; Benham et al., 2021; Samhan et al., 2021; Hu et al., 2022; Philbois et al., 2022; Sanayei et al., 2022; Guo et al., 2023; Kazemi et al., 2023; Mohammadi et al., 2023; Taha et al., 2023) correspond to those in the main text.

The outcomes reported in the included studies were categorized into three domains. Body composition outcomes included BW, BMI, BF%, and FM. Cardiorespiratory fitness was represented by VO_2_max/VO_2_peak. Selected lipid outcomes included HDL-C, LDL-C, TC, and TG. Overall, the included studies differed in HIIT modality, intervention duration, training frequency, intensity prescription, supervision, adherence reporting, and comparator conditions. The pooled estimates should therefore be interpreted in light of this clinical and methodological heterogeneity. The basic characteristics of the included studies are presented in **Table 2**.

Control-group sample sizes, comparator conditions, and relevant co-interventions are summarized in **Table 2**. Detailed information on randomized and completed participants, attrition and reasons for withdrawal, adherence or attendance, supervision, adverse events, prescribed training dose, and actual completed dose is provided in **Supplementary Table 6**. Relevant considerations regarding outcome measurement and assessor blinding are reported in **Supplementary Table 5**. Reporting was heterogeneous across the included trials. Prescribed exercise modality, intensity, weekly frequency, and intervention duration were generally available, whereas session attendance, adherence definitions, actual completed exercise dose, and adverse-event information were not reported consistently. Dropouts occurred in several trials, but the completeness and detail of the reported reasons varied. Exercise supervision was described in a number of trials, although the supervision setting, personnel, and degree of monitoring were not standardized. Accordingly, feasibility and safety could not be quantitatively synthesized, and absence of adverse-event reporting was not interpreted as evidence of absence.

The revised RoB 2 assessments are summarized in **Figures 2**, **3**, with detailed supporting judgements provided in **Supplementary Table 5**. In the conservative study-level summary, 11 of the 15 trials raised some concerns and four were judged to be at high risk of bias; no trial was judged to be at overall low risk. High-risk judgements were driven primarily by substantial or unevenly distributed missing outcome data. Other concerns mainly reflected insufficient reporting of random-sequence generation or allocation concealment, potential susceptibility of effort- or operator-dependent measurements to knowledge of intervention assignment, and the absence of prospectively available protocols or sufficiently detailed analysis plans. The inability to blind participants and exercise personnel was not considered sufficient on its own for a high-risk judgement. Potential differences across outcome types were considered when judging missing outcome data, outcome measurement, and selection of the reported result.

**Figure 2:**
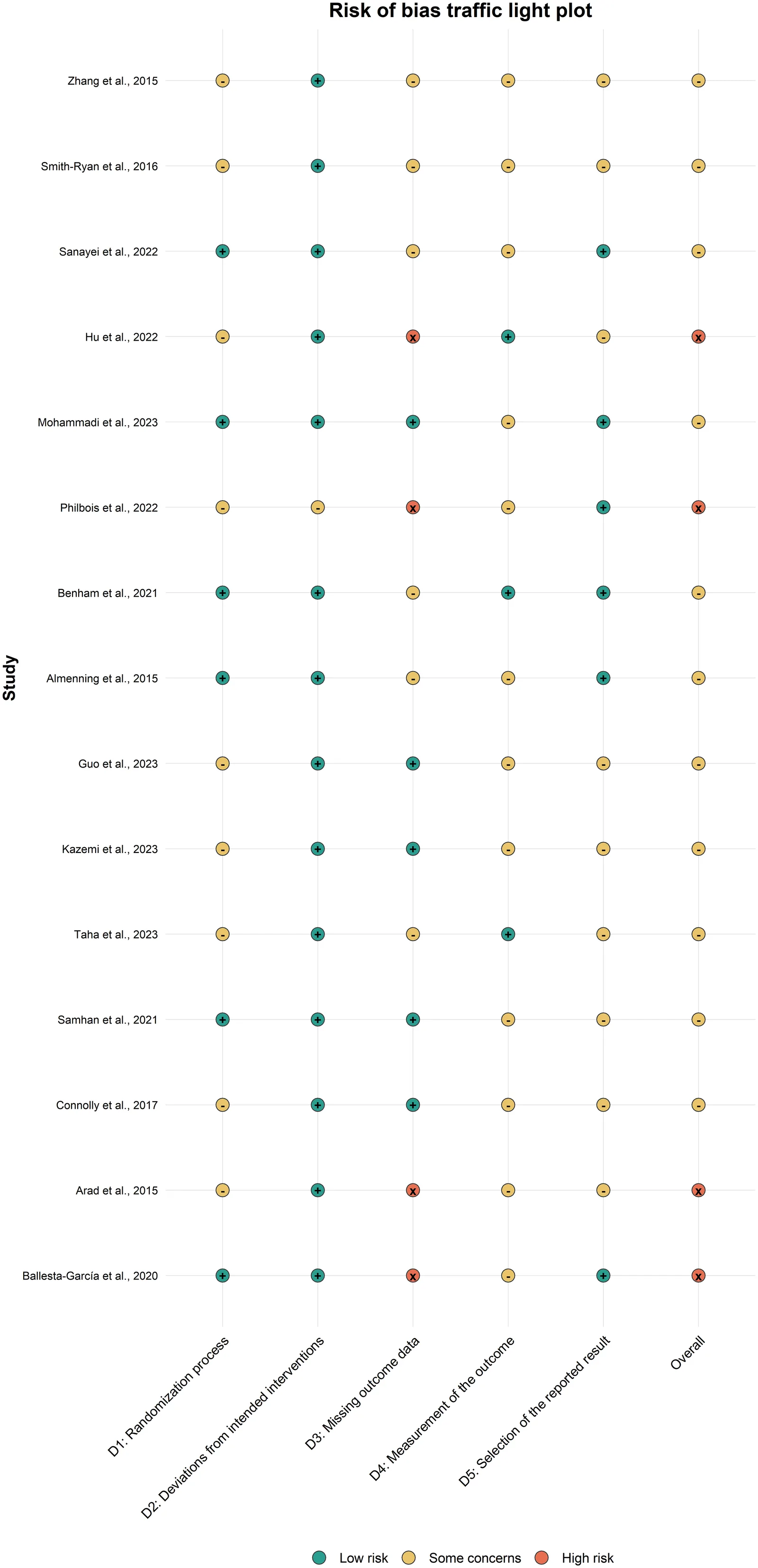
Traffic-light plot of study-level RoB 2 judgements for the included randomized controlled trials.

**Figure 3:**
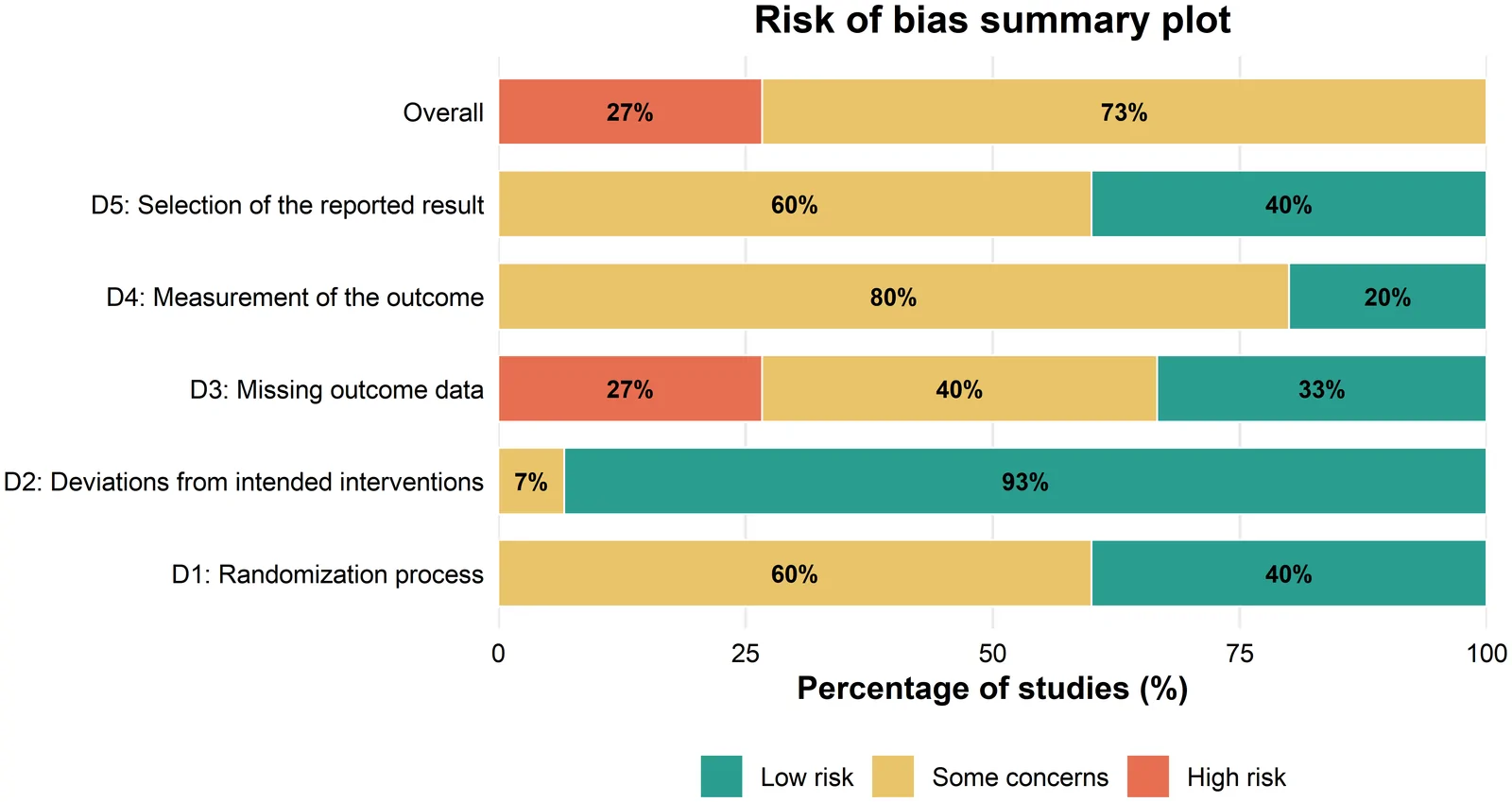
Distribution of study-level RoB 2 judgements across the included randomized controlled trials.

### 3.3 Effects of HIIT on body composition outcomes

#### 3.3.1 Effects of HIIT on BODY WEIGHT

A total of 11 studies involving 336 participants contributed data to the body weight outcome. As shown in **Figure 4**, the random-effects model indicated that, compared with control conditions, HIIT was associated with a significant reduction in body weight, with a pooled effect size of Hedges’ *g* = −0.33, 95% CI −0.58 to −0.08; *p* = 0.01. The heterogeneity test indicated low between-study heterogeneity (*Q*(10) = 14.02, *p* = 0.17; *I2* = 24.23%). These findings suggest that HIIT may be associated with a modest reduction in body weight, with relatively consistent effects across studies. All moderator analyses reported below were exploratory and hypothesis-generating. The moderator domains and coding categories were prespecified before statistical analyses. The number of independent effect estimates and participants contributing to each subgroup is reported as K and N, respectively, in **Tables 3**-**11**. Some moderator categories contained only one or two effect estimates; these estimates are retained for transparent reporting but are not interpreted as reliable evidence of effect modification. The exact number of independent effect estimates contributing to each meta-regression model and relevant limitations in moderator distribution are summarized in **Supplementary Table 4**.

**Figure 4:**
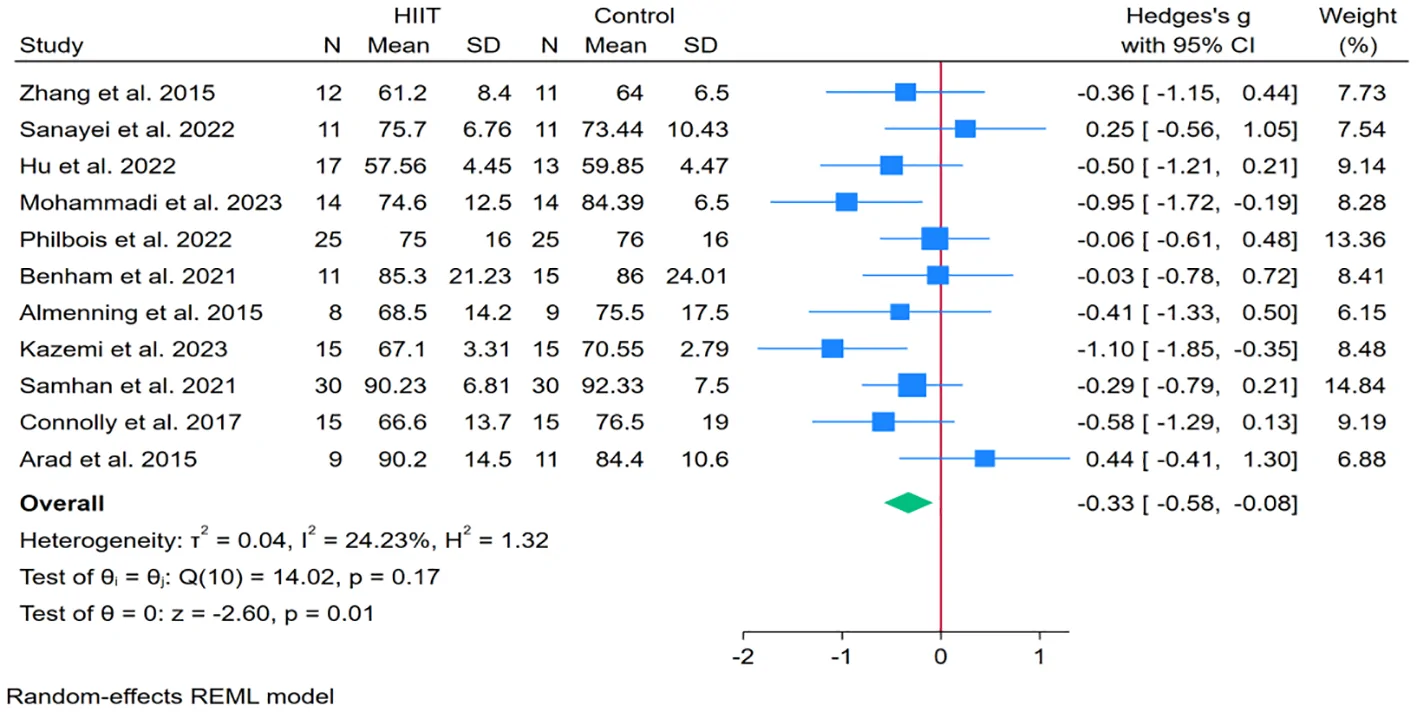
Forest plot of the effects of HIIT on body weight. Squares represent study-specific effect estimates, with the size of each square proportional to the study weight. Horizontal lines indicate 95% confidence intervals (CIs). The diamond represents the pooled effect estimate, and its width indicates the 95% CI. Effect sizes are expressed as Hedges’ *g* and were pooled using a random-effects restricted maximum likelihood (REML) model. Negative values favor HIIT for body weight reduction.

**Table 3:** Subgroup analysis based on the meta-analysis results for body weight.

Subgroup		K (N)	Hedges’ g	95%CI	Pd	Q	I^2^ (%)	Pm
Age stage								0.17
	<40 years	8 (216)	−0.21	−0.49 to 0.08	0.16	8.61	14.76	
	≥40 years	3 (120)	−0.59	−1.05 to −0.13	0.01	3.09	35.89	
Baseline BMI								0.63
	BMI<30 kg/m^2^	7 (202)	−0.38	−0.69 to −0.07	0.02	7.56	22.1	
	BMI ≥30 kg/m^2^	4 (134)	−0.23	−0.74 to 0.28	0.37	6.17	52.16	
Intervention duration								0.20
	≤8 weeks	5 (170)	−0.51	−0.94 to −0.08	0.02	7.76	48.1	
	>8 weeks	6 (166)	−0.17	−0.46 to 0.13	0.28	4.04	0	
Training frequency								0.68
	≤3 sessions/week	9 (283)	−0.31	−0.62 to 0.00	0.05	13.75	40.86	
	>3 sessions/week	2 (53)	−0.44	−0.97 to 0.10	0.11	0.07	0	
Health status								0.59
	General overweight/obesity or abnormal body composition	5 (125)	−0.19	−0.58 to 0.20	0.34	5.3	22.86	
	PCOS	4 (121)	−0.32	−0.75 to 0.11	0.14	4.12	29.14	
	Comorbid disease or special health condition	2 (90)	−0.64	−1.43 to 0.14	0.11	3.08	67.48	
Exercise modality								0.21
	Running-/walking-based HIIT	6 (200)	−0.28	−0.55 to 0.00	0.05	5.38	0	
	Cycling-based HIIT	3 (76)	−0.09	−0.67 to 0.48	0.75	3.35	0.13	
	Circuit-/functional-training-based HIIT	2 (60)	−0.79	−1.37 to −0.20	0.01	1.28	21.7	

K (N) denotes the number of effect sizes included in the pooled estimate and the total number of participants contributing to that estimate. Hedges’ *g* represents the pooled effect size; negative values indicate a greater reduction in BW with HIIT compared with the control condition. 95% CI, 95% confidence interval; *P*d, *p* value for the pooled effect estimate within each subgroup; *Q* and *I2*, heterogeneity statistics; *P*m, *p* value for between-subgroup differences; PCOS, polycystic ovary syndrome; HIIT, high-intensity interval training; BW, body weight; BMI, body mass index.

**Table 4:** Subgroup analysis based on the meta-analysis results for BMI.

Subgroup		K (N)	Hedges’g	95%CI	Pd	Q	I^2^ (%)	Pm
Age stage								0.55
	<40 years	7 (194)	−0.46	−0.84 to −0.08	0.02	10.66	43.93	
	≥40 years	4 (149)	−0.77	−1.70 to 0.17	0.11	17.73	86.35	
Baseline BMI								0.03
	BMI<30 kg/m^2^	6 (180)	−0.90	−1.47 to −0.34	<0.01	15.73	69.45	
	BMI ≥30 kg/m^2^	5 (163)	−0.17	−0.48 to 0.13	0.26	4.56	0	
Intervention duration								0.11
	≤8 weeks	4 (148)	−1.07	−1.96 to −0.17	0.02	19.32	84.1	
	>8 weeks	7 (195)	−0.30	−0.57 to −0.02	0.04	4.57	0	
Training frequency								0.13
	≤3 sessions/week	9 (290)	−0.47	−0.91 to −0.03	0.04	23.71	70.5	
	>3 sessions/week	2 (53)	−1.02	−1.58 to −0.45	<0.01	0.85	0	
Health status								0.68
	General overweight/obesity or abnormal body composition	5 (132)	−0.53	−1.01 to −0.05	0.03	7.69	47.3	
	PCOS	4 (121)	−0.36	−0.71 to −0.01	0.05	2.78	0	
	Comorbid disease or special health condition	2 (90)	−1.16	−3.30 to 0.99	0.29	17.2	94.18	
Exercise modality								0.18
	Running-/walking-based HIIT	5 (178)	−0.38	−0.67 to −0.09	0.01	3.38	0	
	Cycling-based HIIT	3 (76)	−0.11	−0.63 to 0.41	0.68	2.71	27.59	
	Circuit-/functional-training-based HIIT	3 (89)	−1.26	−2.37 to −0.14	0.03	11.46	83.01	

K (N) denotes the number of effect sizes included in the pooled estimate and the total number of participants contributing to that estimate. Hedges’ *g* represents the pooled effect size; negative values indicate a greater reduction in BMI with HIIT compared with the control condition. 95% CI, 95% confidence interval; *P*d, *p* value for the pooled effect estimate within each subgroup; *Q* and *I2*, heterogeneity statistics; *P*m, *p* value for between-subgroup differences; PCOS, polycystic ovary syndrome; HIIT, high-intensity interval training; BMI, body mass index.

**Table 5:** Subgroup analysis based on the meta-analysis results for body fat percentage.

Subgroup		K (N)	Hedges’ g	95%CI	Pd	Q	I^2^ (%)	Pm
Age stage								0.72
	<40 years	8 (207)	−0.82	−1.59 to −0.04	0.04	37.52	85.88	
	≥40 years	2 (90)	−0.65	−1.20 to −0.09	0.02	1.65	39.23	
Baseline BMI								0.66
	BMI<30 kg/m^2^	6 (170)	−0.90	−1.92 to 0.12	0.08	33.97	89.88	
	BMI ≥30 kg/m^2^	4 (127)	−0.65	−1.13 to −0.17	0.01	5.18	41.5	
Intervention duration								0.43
	≤8 weeks	6 (207)	−0.95	−1.93 to 0.04	0.06	32.94	90.82	
	>8 weeks	4 (90)	−0.50	−1.03 to 0.03	0.07	5.03	39.73	
Training frequency								0.08
	≤3 sessions/week	8 (244)	−0.45	−0.77 to −0.13	0.01	11.02	36.67	
	>3 sessions/week	2 (53)	−2.37	−4.47 to −0.26	0.03	8.73	88.54	
Health status								0.97
	General overweight/obesity or abnormal body composition	7 (192)	−0.75	−1.64 to 0.15	0.10	36.27	88.66	
	PCOS	2 (45)	−0.85	−1.54 to −0.15	0.02	1.35	25.72	
	Comorbid disease or special health condition	1 (60)	−0.88	−1.40 to −0.36	<0.01	n/a	n/a	
Exercise modality								0.27
	Running-/walking-based HIIT	5 (150)	−0.70	−1.27 to −0.12	0.02	10.7	64.63	
	Cycling-based HIIT	3 (69)	−0.22	−0.68 to 0.24	0.34	0.38	0	
	Circuit-/functional-training-based HIIT	2 (78)	−1.94	−4.87 to 0.99	0.20	21.68	95.39	

K (N) denotes the number of effect sizes included in the pooled estimate and the total number of participants contributing to that estimate. Hedges’ *g* represents the pooled effect size; negative values indicate a greater reduction in BF% with HIIT compared with the control condition. 95% CI, 95% confidence interval; *P*d, *p* value for the pooled effect estimate within each subgroup; *Q* and *I2*, heterogeneity statistics; *P*m, *p* value for between-subgroup differences; n/a, not applicable; PCOS, polycystic ovary syndrome; HIIT, high-intensity interval training; BF%, body fat percentage; BMI, body mass index.

**Table 6:** Subgroup analysis based on the meta-analysis results for fat mass.

Subgroup		K (N)	Hedges’ g	95%CI	Pd	Q	I^2^ (%)	Pm
Age stage								0.57
	<40 years	5 (109)	−0.78	−1.59 to 0.04	0.06	16.9	76.94	
	≥40 years	3 (120)	−0.52	−0.88 to −0.16	<0.01	0.1	0	
Baseline BMI								0.23
	BMI<30 kg/m^2^	5 (130)	−0.88	−1.55 to −0.20	0.01	13.05	71.63	
	BMI ≥30 kg/m^2^	3 (99)	−0.38	−0.82 to 0.06	0.09	2.4	16.16	
Intervention duration								0.26
	≤8 weeks	4 (139)	−0.93	−1.77 to −0.09	0.03	12.75	80.93	
	>8 weeks	4 (90)	−0.40	−0.80 to 0.01	0.05	2.94	0	
Training frequency								0.14
	≤3 sessions/week	6 (176)	−0.43	−0.72 to −0.13	<0.01	2.44	0	
	>3 sessions/week	2 (53)	−1.56	−3.04 to −0.09	0.04	5.73	82.56	
Health status								0.84
	General overweight/obesity or abnormal body composition	5 (122)	−0.77	−1.57 to 0.02	0.06	17.05	78.37	
	PCOS	1 (17)	−0.44	−1.36 to 0.47	0.34	n/a	n/a	
	Comorbid disease or special health condition	2 (90)	−0.54	−0.95 to −0.12	0.01	0.08	0	
Exercise modality								0.37
	Running-/walking-based HIIT	3 (100)	−0.61	−1.00 to −0.22	<0.01	0.4	0	
	Cycling-based HIIT	3 (69)	−0.29	−0.75 to 0.18	0.22	1.74	0	
	Circuit-/functional-training-based HIIT	2 (60)	−1.37	−3.21 to 0.47	0.14	10.1	90.1	

K (N) denotes the number of effect sizes included in the pooled estimate and the total number of participants contributing to that estimate. Hedges’ *g* represents the pooled effect size; negative values indicate a greater reduction in FM with HIIT compared with the control condition. 95% CI, 95% confidence interval; *P*d, *p* value for the pooled effect estimate within each subgroup; *Q* and *I2*, heterogeneity statistics; *P*m, *p* value for between-subgroup differences; n/a, not applicable; PCOS, polycystic ovary syndrome; HIIT, high-intensity interval training; FM, fat mass; BMI, body mass index.

**Table 7:** Subgroup analysis based on the meta-analysis results for maximal or peak oxygen uptake.

Subgroup		K (N)	Hedges’ g	95%CI	Pd	Q	I^2^ (%)	Pm
Age stage								0.4
	<40 years	9 (250)	1.18	0.65 to 1.70	<0.01	27.83	72.96	
	≥40 years	3 (120)	0.92	0.53 to 1.31	<0.01	1.94	8.14	
Baseline BMI								0.48
	BMI<30 kg/m^2^	7 (220)	1.21	0.73 to 1.68	<0.01	15.44	62.22	
	BMI ≥30 kg/m^2^	5 (150)	0.91	0.23 to 1.59	0.01	14.12	73.65	
Intervention duration								0.54
	≤8 weeks	6 (207)	1.21	0.68 to 1.74	<0.01	14.39	67.22	
	>8 weeks	6 (163)	0.96	0.37 to 1.55	<0.01	14.05	68.7	
Training frequency								<0.01
	≤3 sessions/week	11 (347)	0.98	0.64 to 1.32	<0.01	22.18	55.86	
	>3 sessions/week	1 (23)	2.66	1.56 to 3.76	<0.01	n/a	n/a	
Health status								0.8
	General overweight/obesity or abnormal body composition	6 (160)	1.23	0.59 to 1.86	<0.01	16.21	70.6	
	PCOS	4 (120)	1	0.20 to 1.80	0.01	11.28	75.9	
	Comorbid disease or special health condition	2 (90)	0.94	0.35 to 1.52	<0.01	1.75	42.72	
Exercise modality								0.02
	Running-/walking-based HIIT	6 (200)	1.41	0.91 to 1.92	<0.01	11.65	59.85	
	Cycling-based HIIT	4 (92)	0.49	0.09 to 0.90	0.02	2	0	
	Circuit-/functional-training-based HIIT	2 (78)	1.22	−0.00 to 2.44	0.05	6.27	84.06	

K (N) denotes the number of effect sizes included in the pooled estimate and the total number of participants contributing to that estimate. Hedges’ *g* represents the pooled effect size; positive values indicate a greater improvement in VO_2_max/VO_2_peak with HIIT compared with the control condition. 95% CI, 95% confidence interval; *P*d, *p* value for the pooled effect estimate within each subgroup; *Q* and *I2*, heterogeneity statistics; *P*m, *p* value for between-subgroup differences; n/a, not applicable; PCOS, polycystic ovary syndrome; HIIT, high-intensity interval training; VO_2_max, maximal oxygen uptake; VO_2_peak, peak oxygen uptake; BMI, body mass index.

**Table 8:** Subgroup Analysis based on the meta-analysis results for high-density lipoprotein cholesterol.

Subgroup		K (N)	Hedges’ g	95%CI	Pd	Q	I^2^ (%)	Pm
Age stage								0.03
	<40 years	5 (140)	0.35	−0.14 to 0.84	0.16	8.24	51.7	
	≥40 years	3 (113)	1.56	0.59 to 2.53	<0.01	8.41	80.3	
Baseline BMI								0.68
	BMI<30 kg/m^2^	4 (127)	1	−0.12 to 2.12	0.08	24.77	88.21	
	BMI ≥30 kg/m^2^	4 (126)	0.72	0.04 to 1.40	0.04	10.46	70.1	
Intervention duration								0.29
	≤8 weeks	3 (77)	1.36	0.02 to 2.69	0.05	14.5	85.76	
	>8 weeks	5 (176)	0.57	−0.03 to 1.16	0.06	16.45	72.75	
Training frequency								n/a
	≤3 sessions/week	8 (253)	0.84	0.24 to 1.45	0.01	35.38	80.9	
	>3 sessions/week	n/a	n/a	n/a	n/a	n/a	n/a	
Health status								<0.01
	General overweight/obesity or abnormal body composition	2 (49)	0.99	0.42 to 1.57	<0.01	0.43	<0.01	
	PCOS	4 (121)	0.13	−0.24 to 0.50	0.49	3.47	6.74	
	Comorbid disease or special health condition	2 (83)	1.93	0.69 to 3.16	<0.01	4.83	79.31	
Exercise modality								<0.01
	Running-/walking-based HIIT	3 (95)	0.22	−0.31 to 0.75	0.42	3.35	39.83	
	Cycling-based HIIT	4 (128)	0.87	0.25 to 1.48	0.01	8.29	63.27	
	Circuit-/functional-training-based HIIT	1 (30)	2.62	1.66 to 3.58	<0.01	n/a	n/a	

K (N) denotes the number of effect sizes included in the pooled estimate and the total number of participants contributing to that estimate. Hedges’ *g* represents the pooled effect size; positive values indicate a greater increase in HDL-C with HIIT compared with the control condition. 95% CI, 95% confidence interval; *P*d, *p* value for the pooled effect estimate within each subgroup; *Q* and *I2*, heterogeneity statistics; *P*m, *p* value for between-subgroup differences; n/a, not applicable; PCOS, polycystic ovary syndrome; HIIT, high-intensity interval training; HDL-C, high-density lipoprotein cholesterol; BMI, body mass index.

**Table 9:** Subgroup analysis based on the meta-analysis results for low-density lipoprotein cholesterol.

Subgroup		K (N)	Hedges’ g	95%CI	Pd	Q	I^2^ (%)	Pm
Age stage								0.03
	<40 years	5 (140)	−0.38	−0.79 to 0.03	0.07	5.75	32.55	
	≥40 years	3 (113)	−1.39	−2.20 to −0.58	<0.01	7.97	73.89	
Baseline BMI								0.54
	BMI<30 kg/m^2^	4 (127)	−0.60	−1.21 to −0.00	0.05	8.52	63.88	
	BMI ≥30 kg/m^2^	4 (126)	−0.94	−1.82 to −0.06	0.04	16.85	81.27	
Intervention duration								0.73
	≤8 weeks	3 (77)	−0.90	−1.72 to −0.08	0.03	6.03	67.53	
	>8 weeks	5 (176)	−0.71	−1.42 to −0.00	0.05	22.65	80.38	
Training frequency								n/a
	≤3 sessions/week	8 (253)	−0.78	−1.29 to −0.27	<0.01	29.41	74.16	
	>3 sessions/week	n/a	n/a	n/a	n/a	n/a	n/a	
Health status								<0.01
	General overweight/obesity or abnormal body composition	2 (49)	−0.40	−0.98 to 0.18	0.18	1.1	8.72	
	PCOS	4 (121)	−0.45	−0.94 to 0.03	0.07	5.19	43.37	
	Comorbid disease or special health condition	2 (83)	−1.79	−2.37 to −1.22	<0.01	1.28	22	
Exercise modality								0.19
	Running-/walking-based HIIT	3 (95)	−0.49	−1.17 to 0.18	0.15	5.19	60.41	
	Cycling-based HIIT	4 (128)	−0.81	−1.68 to 0.06	0.07	17.52	81.81	
	Circuit-/functional-training-based HIIT	1 (30)	−1.45	−2.24 to −0.67	<0.01	n/a	n/a	

K (N) denotes the number of effect sizes included in the pooled estimate and the total number of participants contributing to that estimate. Hedges’ *g* represents the pooled effect size; negative values indicate a greater reduction in LDL-C with HIIT compared with the control condition. 95% CI, 95% confidence interval; *P*d, *p* value for the pooled effect estimate within each subgroup; *Q* and *I2*, heterogeneity statistics; *P*m, *p* value for between-subgroup differences; n/a, not applicable; PCOS, polycystic ovary syndrome; HIIT, high-intensity interval training; LDL-C, low-density lipoprotein cholesterol; BMI, body mass index.

**Table 10:** Subgroup analysis based on the meta-analysis results for total cholesterol.

Subgroup		K(N)	Hedges’ g	95%CI	Pd	Q	I^2^ (%)	Pm
Age stage								<0.01
	<40 years	6 (163)	-0.05	−0.35 to 0.25	0.76	3.35	0	
	≥40 years	3 (113)	-0.92	−1.30 to −0.54	<0.01	1.67	0	
Baseline BMI								0.75
	BMI<30 kg/m^2^	5 (150)	-0.29	−0.82 to 0.24	0.28	10.14	61.97	
	BMI ≥30 kg/m^2^	4 (126)	-0.41	−0.94 to 0.12	0.13	6.59	54.2	
Intervention duration								0.69
	≤8 weeks	3 (77)	-0.47	−1.32 to 0.37	0.27	6.76	71.29	
	>8 weeks	6 (199)	-0.29	−0.69 to 0.12	0.17	10.26	51.13	
Training frequency								0.07
	≤3 sessions/week	8 (253)	-0.43	−0.78 to −0.08	0.02	13.64	48.18	
	>3 sessions/week	1 (23)	0.38	−0.42 to 1.18	0.35	n/a	n/a	
Health status								<0.01
	General overweight/obesity or abnormal body composition	3 (72)	0.01	−0.61 to 0.63	0.97	3.68	46.13	
	PCOS	4 (121)	-0.19	−0.54 to 0.16	0.29	1	0	
	Comorbid disease or special health condition	2 (83)	-1.07	−1.52 to −0.62	<0.01	0.23	0	
Exercise modality								0.02
	Running-/walking-based HIIT	4 (118)	-0.06	−0.41 to 0.29	0.75	2.39	0	
	Cycling-based HIIT	4 (128)	-0.44	−0.97 to 0.09	0.10	6.58	54.19	
	Circuit-/functional-training-based HIIT	1 (30)	-1.22	−1.98 to −0.46	<0.01	n/a	n/a	

K (N) denotes the number of effect sizes included in the pooled estimate and the total number of participants contributing to that estimate. Hedges’ *g* represents the pooled effect size; negative values indicate a greater reduction in TC with HIIT compared with the control condition. 95% CI, 95% confidence interval; *P*d, *p* value for the pooled effect estimate within each subgroup; *Q* and *I2*, heterogeneity statistics; *P*m, *p* value for between-subgroup differences; n/a, not applicable; PCOS, polycystic ovary syndrome; HIIT, high-intensity interval training; TC, total cholesterol; BMI, body mass index.

**Table 11:** Subgroup analysis based on the meta-analysis results for triglycerides.

Subgroup		K (N)	Hedges’ g	95%CI	Pd	Q	I^2^ (%)	Pm
Age stage								0.02
	<40 years	6 (163)	0.1	−0.20 to 0.40	0.51	2.23	0	
	≥40 years	3 (113)	−0.93	−1.76 to −0.09	0.03	8.63	77.51	
Baseline BMI								0.76
	BMI<30 kg/m^2^	5 (150)	−0.17	−0.85 to 0.51	0.62	15.14	76.45	
	BMI ≥30 kg/m^2^	4 (126)	−0.32	−0.99 to 0.35	0.35	11.49	70.89	
Intervention duration								0.58
	≤8 weeks	3 (77)	−0.46	−1.46 to 0.54	0.37	9.71	79.27	
	>8 weeks	6 (199)	−0.14	−0.66 to 0.38	0.60	17.47	69.93	
Training frequency								0.72
	≤3 sessions/week	8 (253)	−0.26	−0.76 to 0.25	0.32	27.62	74.85	
	>3 sessions/week	1 (23)	−0.09	−0.88 to 0.70	0.83	n/a	n/a	
Health status								<0.01
	General overweight/obesity or abnormal body composition	3 (72)	−0.06	−0.50 to 0.39	0.81	0.04	0	
	PCOS	4 (121)	0.15	−0.20 to 0.50	0.39	1.89	0	
	Comorbid disease or special health condition	2 (83)	−1.32	−1.79 to −0.85	<0.01	0.25	0	
Exercise modality								<0.01
	Running-/walking-based HIIT	4 (118)	0.14	−0.22 to 0.49	0.44	2.08	0	
	Cycling-based HIIT	4 (128)	−0.36	−1.00 to 0.28	0.27	10.51	68.77	
	Circuit-/functional-training-based HIIT	1 (30)	−1.48	−2.27 to −0.69	<0.01	n/a	n/a	

K (N) denotes the number of effect sizes included in the pooled estimate and the total number of participants contributing to that estimate. Hedges’ *g* represents the pooled effect size; negative values indicate a greater reduction in TG with HIIT compared with the control condition. 95% CI, 95% confidence interval; *P*d, *p* value for the pooled effect estimate within each subgroup; *Q* and *I2*, heterogeneity statistics; *P*m, *p* value for between-subgroup differences; n/a, not applicable; PCOS, polycystic ovary syndrome; HIIT, high-intensity interval training; TG, triglycerides; BMI, body mass index.

Subgroup analyses showed that differences in the effects of HIIT on body weight reduction according to age stage, baseline BMI, intervention duration, training frequency, health status, and exercise modality did not reach statistical significance (*p* > 0.05), suggesting that these factors did not show clear evidence of effect modification at this stage (**Table 3**). Further inspection of the subgroup results indicated that the reduction in body weight appeared to be relatively more pronounced among participants aged ≥40 years, those with baseline BMI<30 kg/m^2^, interventions lasting ≤8 weeks, and HIIT programs involving circuit-based or functional training modalities. However, none of the between-subgroup comparisons reached statistical significance (*p* > 0.05). Therefore, these differences should be interpreted as exploratory findings, and they do not provide sufficient evidence to conclude that age, baseline BMI, intervention duration, training frequency, health status, or exercise modality significantly modifies the effect of HIIT on body weight.

Meta-regression analyses showed that age (*β* = −0.011, 95% CI −0.033 to 0.011; *p* = 0.334), baseline BMI (*β* = 0.043, 95% CI −0.035 to 0.121; *p* = 0.284), intervention duration (*β* = 0.036, 95% CI −0.008 to 0.080; *p* = 0.110), and training frequency (*β* = −0.089, 95% CI −0.515 to 0.337; *p* = 0.683) were not significantly associated with the effect size of HIIT on body weight (all *p* > 0.05; **Figure 5**). These findings suggest that, in the present analysis, there was no clear evidence that these variables moderated the effect of HIIT on body weight.

**Figure 5:**
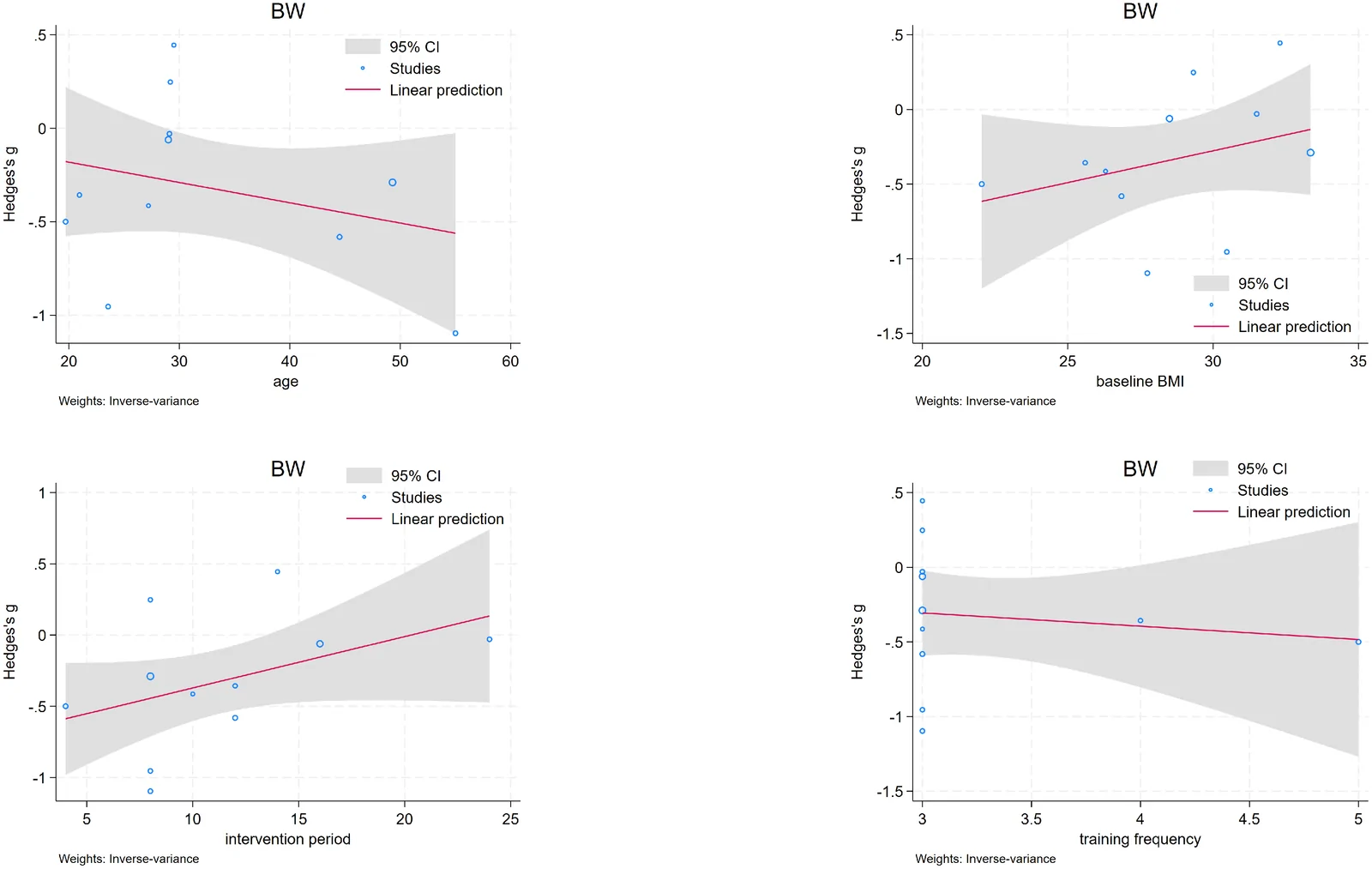
Bubble plots of the univariable meta-regression analyses for body weight. Panels show the associations between the effect size for body weight and age, baseline BMI, intervention duration, and training frequency. Each dot represents an individual effect size, and the shaded area indicates the 95% confidence interval (CI) around the fitted regression line. Effect sizes are expressed as Hedges’ *g*. The fitted lines suggest no clear evidence that age, baseline BMI, intervention duration, or training frequency was associated with the effect size of HIIT on body weight in the present analysis.

#### 3.3.2 Effects of HIIT on body mass index

The forest plot showed that 11 pooled effect sizes involving 343 participants contributed data to the BMI outcome (**Figure 6**). The random-effects model indicated that HIIT was associated with a statistically significant reduction in BMI compared with control conditions (Hedges’ g = −0.560, 95% CI −0.945 to −0.174; P = 0.005). Moderate-to-high between-study heterogeneity was observed (Q = 28.53, P = 0.002; I^2^ = 67.67%, τ^2^ = 0.2810), indicating that the magnitude of the estimated BMI reduction varied across studies. Accordingly, although the pooled estimate favored HIIT, the magnitude and consistency of the effect should be interpreted cautiously in view of the observed heterogeneity.

**Figure 6:**
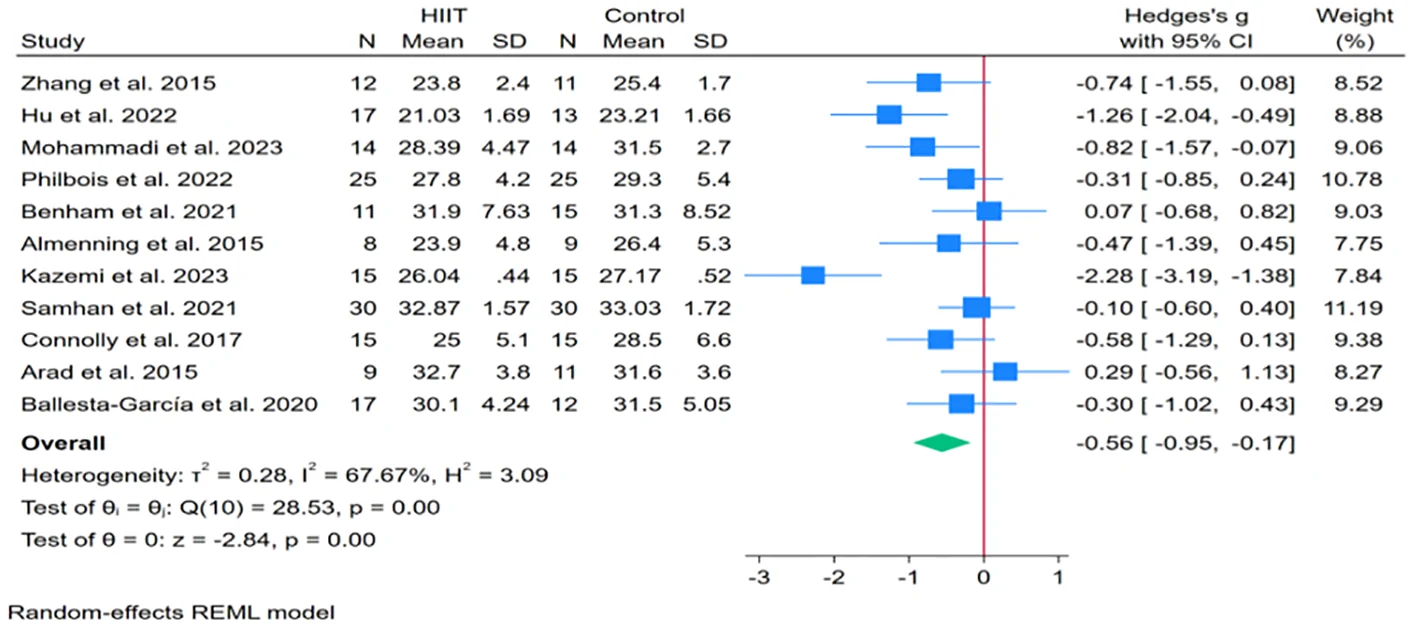
Forest plot of the effects of HIIT on body mass index. Squares represent study-specific effect estimates, with the size of each square proportional to the study weight. Horizontal lines indicate 95% confidence intervals (CIs). The diamond represents the pooled effect estimate, and its width indicates the 95% CI. Effect sizes are expressed as Hedges’ *g* and were pooled using a random-effects restricted maximum likelihood (REML) model. Negative values favor HIIT for reducing body mass index.

Subgroup analyses showed a statistically significant between-subgroup difference according to baseline BMI for the effect of HIIT on BMI reduction (*p* = 0.03). The reduction appeared to be more pronounced among participants with baseline BMI<30 kg/m^2^ (Hedges’ *g* = −0.90, 95% CI −1.47 to −0.34), whereas the effect did not reach statistical significance among those with baseline BMI ≥30 kg/m^2^ (Hedges’ *g* = −0.17, 95% CI −0.48 to 0.13). Differences in the effects of HIIT on BMI reduction according to age stage, intervention duration, training frequency, health status, and exercise modality did not reach statistical significance (*p* > 0.05), suggesting that these factors did not show clear evidence of effect modification in the present analysis (**Table 4**).

Further inspection of the subgroup results indicated that reductions in BMI appeared to be relatively more pronounced among participants aged ≥40 years, interventions lasting ≤8 weeks, interventions with a training frequency >3 sessions/week, and HIIT programs involving circuit-based or functional training modalities. However, the corresponding between-subgroup comparisons did not reach statistical significance (*p* > 0.05). Therefore, except for baseline BMI, which may have a potential effect-modifying role, the remaining subgroup differences should be interpreted as exploratory findings and do not provide sufficient evidence to conclude that age stage, intervention duration, training frequency, health status, or exercise modality significantly modifies the effect of HIIT on BMI.

Univariable meta-regression analyses did not show clear evidence of a study-level association between the BMI effect estimate and mean participant age (β = −0.003, 95% CI −0.031 to 0.024; P = 0.806; estimated R^2^ = 0.00%; residual I^2^ = 71.36%) or training frequency (β = −0.327, 95% CI −0.853 to 0.199; P = 0.223; estimated R^2^ = 9.39%; residual I^2^ = 65.73%). Baseline BMI showed a statistically significant positive study-level association with the BMI effect estimate (β = 0.116, 95% CI 0.023 to 0.210; P = 0.014). The baseline-BMI model accounted for an estimated 57.33% of the between-study variance, with residual I^2^ decreasing to 46.60% and residual τ^2^ to 0.1199. Intervention duration also showed a statistically significant positive study-level association with the BMI effect estimate (β = 0.067, 95% CI 0.005 to 0.130; P = 0.035). The intervention-duration model accounted for an estimated 35.82% of the between-study variance, with residual I^2^ decreasing to 57.07% and residual τ^2^ to 0.1804. Because negative Hedges’ g values indicate a greater reduction in BMI with HIIT, the positive coefficients suggest that studies involving participants with a higher mean baseline BMI or a longer intervention duration tended to report less-negative BMI effect estimates. However, these associations should be interpreted cautiously because only 11 effect sizes were available, the analyses were conducted at the study level, and the corresponding categorical subgroup comparison for intervention duration did not reach statistical significance. Accordingly, baseline BMI and intervention duration may represent potential study-level moderators of variation in the BMI effect estimates, but these findings should be regarded as exploratory and hypothesis-generating and should not be interpreted as causal (**Figure 7**).

**Figure 7:**
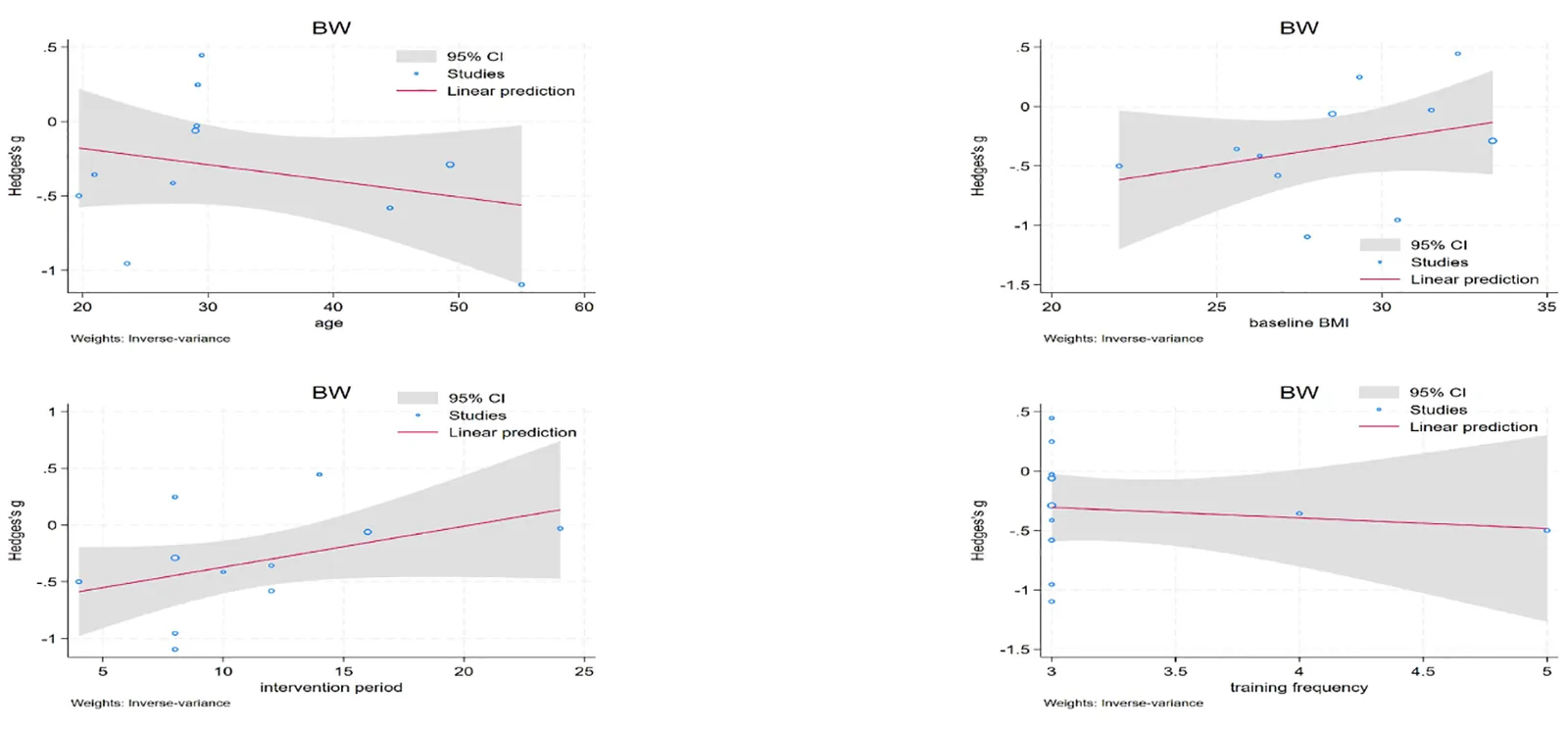
Bubble plots of the univariable meta-regression analyses for body mass index. Panels show the study-level associations between the BMI effect estimates and mean participant age, baseline BMI, intervention duration, and training frequency. Each dot represents an individual effect size, and the shaded area indicates the 95% confidence interval around the fitted regression line. Effect sizes are expressed as Hedges’ g. Baseline BMI and intervention duration showed statistically significant positive associations with the BMI effect estimate, whereas no clear evidence of an association was observed for mean participant age or training frequency. Because only 11 effect sizes were available and the analyses were based on study-level characteristics, these findings should be regarded as exploratory and should not be interpreted as causal.

Leave-one-out sensitivity analysis showed that the pooled BMI effect estimates ranged from −0.633 to −0.396 after the sequential omission of individual studies, while I^2^ ranged from 25.18% to 71.80% and τ^2^ ranged from 0.0435 to 0.3382. The pooled effect remained negative and statistically significant in all analyses, with corresponding P values ranging from 0.001 to 0.015. Thus, omission of any individual study did not alter the direction or statistical significance of the pooled BMI effect. Exclusion of Kazemi et al. (2023) produced the most pronounced change, attenuating the pooled estimate from −0.560 to −0.396, reducing I^2^ from 67.67% to 25.18%, and reducing τ^2^ from 0.2810 to 0.0435. This pattern suggests that Kazemi et al. (2023) may have contributed to the magnitude of the pooled estimate and to part of the observed between-study heterogeneity, although it does not establish the study as a definitive source of heterogeneity. Omission of the remaining studies did not result in a similarly marked reduction in heterogeneity. Omission of any individual study did not alter the direction or statistical interpretation of the pooled BMI estimate. However, the magnitude of the effect and the heterogeneity estimates varied, particularly after omission of Kazemi et al. (2023). The magnitude and consistency of the pooled BMI effect therefore remain uncertain and should be interpreted cautiously (**Supplementary Table 3**; **Supplementary Figure 19A**).

#### 3.3.3 Effects of HIIT on body fat percentage

The forest plot showed that 10 pooled effect sizes involving 297 participants contributed data to the body fat percentage outcome (**Figure 8**). The random-effects model indicated that HIIT was associated with a statistically significant reduction in body fat percentage compared with control conditions (Hedges’ g = −0.762, 95% CI −1.354 to −0.169; P = 0.012). Substantial between-study heterogeneity was observed (Q = 39.16, P< 0.001; I^2^ = 83.39%, τ^2^ = 0.7454), indicating considerable variation in the magnitude of the estimated body-fat-percentage reduction across studies. Accordingly, although the pooled estimate favored HIIT, the magnitude and consistency of the effect should be interpreted cautiously in view of the observed heterogeneity.

**Figure 8:**
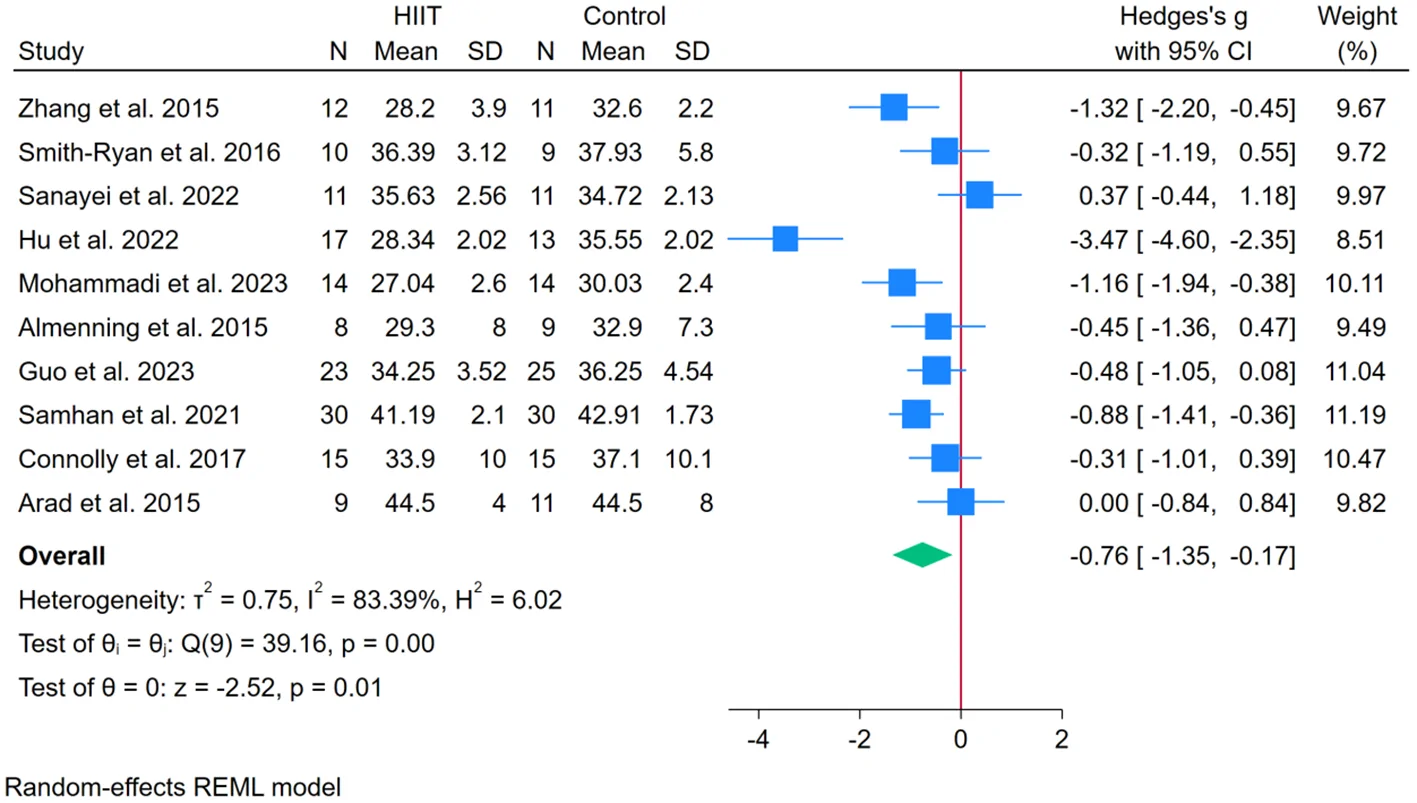
Forest plot of the effects of HIIT on body fat percentage. Squares represent study-specific effect estimates, with the size of each square proportional to the study weight. Horizontal lines indicate 95% confidence intervals (CIs). The diamond represents the pooled effect estimate, and its width indicates the 95% CI. Effect sizes are expressed as Hedges’ *g* and were pooled using a random-effects restricted maximum likelihood (REML) model. Negative values favor HIIT for reducing body fat percentage.

Subgroup analyses showed that differences in the effects of HIIT on body fat percentage reduction according to age stage, baseline BMI, intervention duration, training frequency, health status, and exercise modality did not reach statistical significance (*p* > 0.05), suggesting that these factors did not show clear evidence of effect modification in the present analysis (**Table 5**). Further inspection of the subgroup results indicated that reductions in body fat percentage appeared to be relatively more pronounced among participants aged<40 years, those with baseline BMI<30 kg/m^2^, interventions lasting ≤8 weeks, interventions with a training frequency >3 sessions/week, and HIIT programs involving circuit-based or functional training modalities. However, none of the corresponding between-subgroup comparisons reached statistical significance (*p* > 0.05). Therefore, these differences should be interpreted as exploratory findings and do not provide sufficient evidence to conclude that age stage, baseline BMI, intervention duration, training frequency, health status, or exercise modality significantly modifies the effect of HIIT on body fat percentage.

Univariable meta-regression analyses did not show clear evidence of a study-level association between the body fat percentage effect estimate and mean participant age (β = 0.033, 95% CI −0.028 to 0.094; P = 0.286; estimated R^2^ = 0.00%; residual I^2^ = 82.86%) or intervention duration (β = 0.085, 95% CI −0.085 to 0.255; P = 0.328; estimated R^2^ = 0.00%; residual I^2^ = 83.93%). Baseline BMI showed a positive study-level association that did not reach statistical significance (β = 0.151, 95% CI −0.011 to 0.313; P = 0.068). The baseline-BMI model accounted for an estimated 19.08% of the between-study variance, although substantial residual heterogeneity remained (residual I^2^ = 79.47%; residual τ^2^ = 0.6032); therefore, its potential moderating role remains uncertain. Training frequency showed a statistically significant negative study-level association with the body fat percentage effect estimate (β = −1.357, 95% CI −1.932 to −0.781; P< 0.001). The training-frequency model accounted for an estimated 89.08% of the between-study variance, with residual I^2^ decreasing to 36.51% and residual τ^2^ to 0.0814. Because negative Hedges’ g values indicate a greater reduction in body fat percentage with HIIT, the negative coefficient suggests that studies using a higher training frequency tended to report more-negative effect estimates. However, this association should be interpreted with particular caution because only 10 effect sizes were available, the >3 sessions/week category contained only two effect sizes, and the corresponding between-subgroup comparison did not reach statistical significance. The estimated proportion of explained variance may therefore be unstable and sensitive to the sparse distribution of training frequency and the influence of individual studies. Accordingly, training frequency may represent a potential study-level moderator of variation in the body fat percentage effect estimates, but the finding should be regarded as exploratory and hypothesis-generating and should not be interpreted as causal (**Figure 9**).

**Figure 9:**
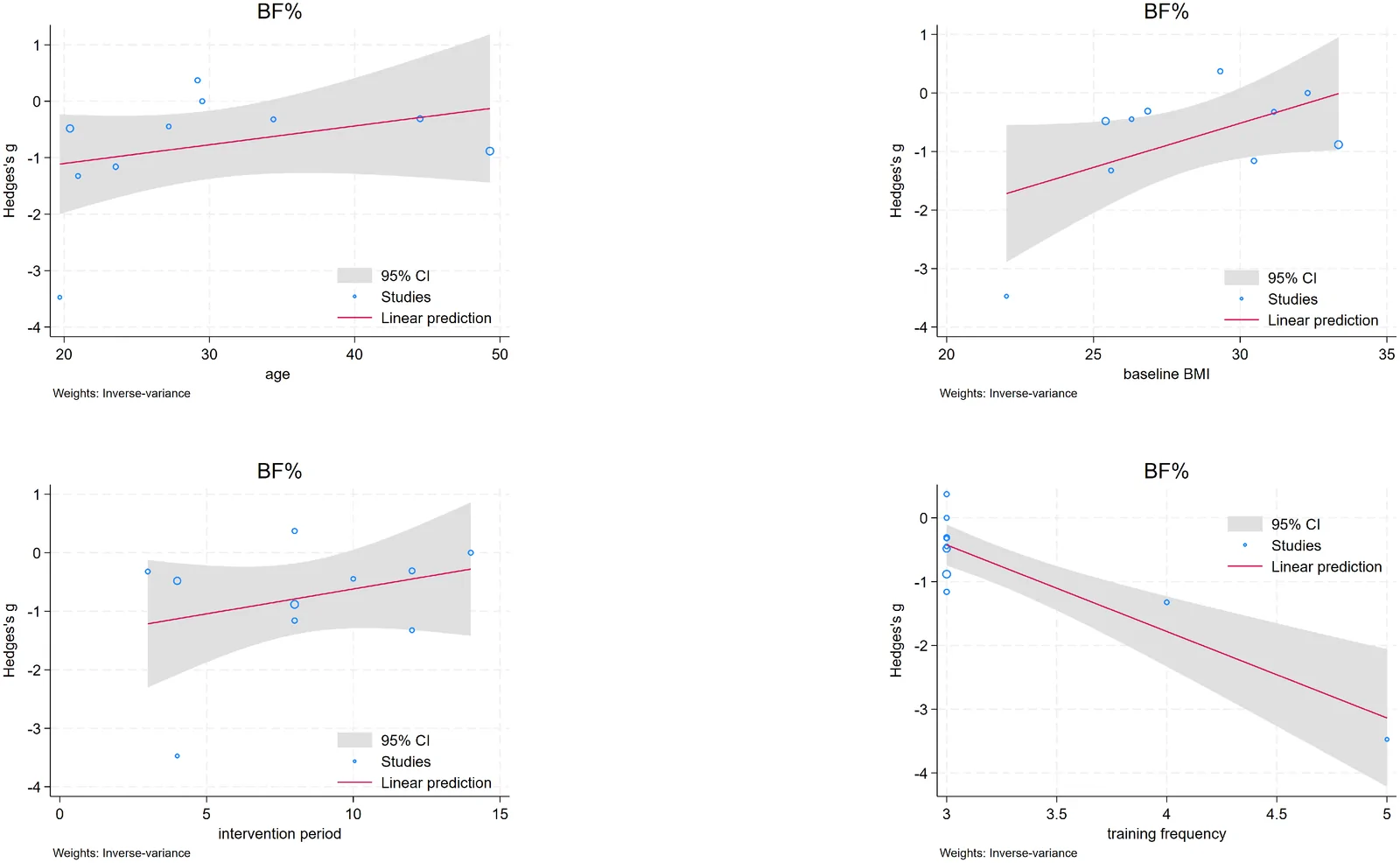
Bubble plots of the univariable meta-regression analyses for body fat percentage. Panels show the study-level associations between the body fat percentage effect estimates and mean participant age, baseline BMI, intervention duration, and training frequency. Each dot represents an individual effect size, and the shaded area indicates the 95% confidence interval around the fitted regression line. Effect sizes are expressed as Hedges’ g. Training frequency showed a statistically significant negative association with the body fat percentage effect estimate. No clear evidence of an association was observed for mean participant age or intervention duration, while the positive association with baseline BMI did not reach statistical significance. Because only 10 effect sizes were available and the >3 sessions/week category contained only two effect sizes, these findings should be regarded as exploratory and should not be interpreted as causal.

Leave-one-out sensitivity analysis showed that the pooled body fat percentage effect estimates ranged from −0.884 to −0.523 after the sequential omission of individual studies, while I^2^ ranged from 43.63% to 85.85% and τ^2^ ranged from 0.1080 to 0.8990. The pooled effect remained negative and statistically significant in all analyses, with corresponding P values ranging from 0.002 to 0.034. Thus, omission of any individual study did not alter the direction or statistical significance of the pooled body fat percentage effect. Exclusion of Hu et al. (2022) produced the most pronounced change, attenuating the pooled estimate from −0.762 to −0.523, reducing I^2^ from 83.39% to 43.63%, and reducing τ^2^ from 0.7454 to 0.1080. This pattern suggests that Hu et al. (2022) may have contributed substantially to the magnitude of the pooled estimate and to part of the observed between-study heterogeneity, although it does not establish the study as a definitive source of heterogeneity. Omission of Sanayei et al. (2022) and Arad et al. (2015) produced smaller changes in the magnitude of the pooled estimate but did not meaningfully reduce heterogeneity. Omission of any individual study did not alter the direction or statistical interpretation of the pooled BF% estimate. However, the effect magnitude and heterogeneity estimates were sensitive to omission of Hu et al. (2022). The magnitude and consistency of the pooled BF% effect therefore remain uncertain and should be interpreted cautiously (**Supplementary Table 3**; **Supplementary Figure 19B**).

#### 3.3.4 Effects of HIIT on fat mass

The forest plot showed that eight pooled effect sizes involving 229 participants contributed data to the fat mass outcome (**Figure 10**). The random-effects model indicated that HIIT was associated with a statistically significant reduction in fat mass compared with control conditions (Hedges’ g = −0.656, 95% CI −1.108 to −0.204; p = 0.004). Moderate-to-high between-study heterogeneity was observed (Q = 17.73, p = 0.013; I^2^ = 64.04%, τ^2^ = 0.2655), indicating that the magnitude of the estimated fat-mass reduction varied across studies. Accordingly, although the pooled estimate favored HIIT, the magnitude and consistency of the effect should be interpreted cautiously in view of the observed heterogeneity.

**Figure 10:**
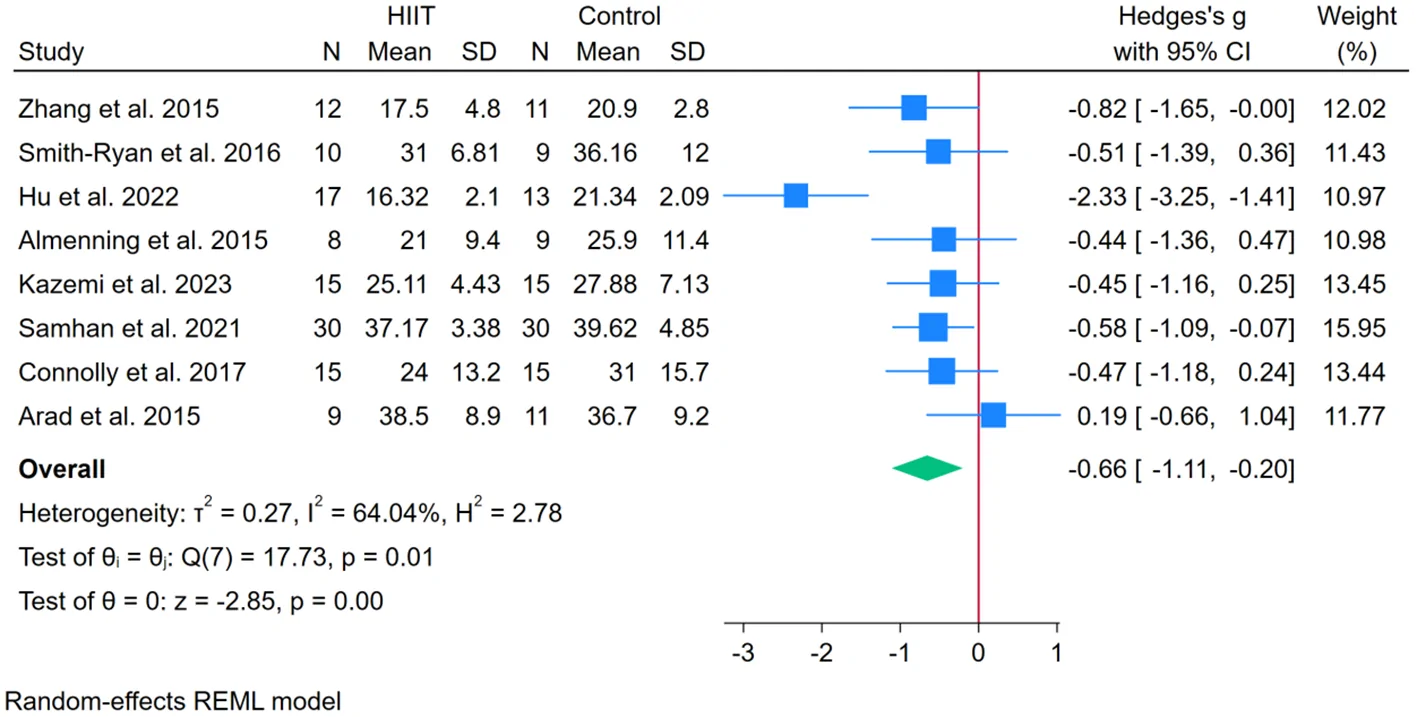
Forest plot of the effects of HIIT on fat mass. Squares represent study-specific effect estimates, with the size of each square proportional to the study weight. Horizontal lines indicate 95% confidence intervals (CIs). The diamond represents the pooled effect estimate, and its width indicates the 95% CI. Effect sizes are expressed as Hedges’ g and were pooled using a random-effects restricted maximum likelihood (REML) model. Negative values favor HIIT for reducing fat mass.

Subgroup analyses showed that differences in the effects of HIIT on fat mass reduction according to age stage, baseline BMI, intervention duration, training frequency, health status, and exercise modality did not reach statistical significance (*p* > 0.05), suggesting that these factors did not show clear evidence of effect modification in the present analysis (**Table 6**). Further inspection of the subgroup results indicated that reductions in fat mass appeared to be relatively more pronounced among participants aged<40 years, those with baseline BMI<30 kg/m^2^, interventions lasting ≤8 weeks, interventions with a training frequency >3 sessions/week, and HIIT programs involving circuit-based or functional training modalities. However, none of the corresponding between-subgroup comparisons reached statistical significance (*p* > 0.05). Therefore, these differences should be interpreted as exploratory findings and do not provide sufficient evidence to conclude that age stage, baseline BMI, intervention duration, training frequency, health status, or exercise modality significantly modifies the effect of HIIT on fat mass.

Univariable meta-regression analysis did not show clear evidence of a study-level association between mean participant age and the fat mass effect estimate (β = 0.021, 95% CI −0.015 to 0.057; p = 0.248; estimated R^2^ = 2.12%; residual I^2^ = 63.07%). Baseline BMI showed a statistically significant positive study-level association with the fat mass effect estimate (β = 0.119, 95% CI 0.015 to 0.222; p = 0.024). The baseline-BMI model accounted for an estimated 51.21% of the between-study variance, with residual I^2^ decreasing to 45.19% and residual τ^2^ to 0.1295. Because negative Hedges’ g values indicate a greater reduction in fat mass with HIIT, the positive regression coefficient suggests that studies involving participants with a higher mean baseline BMI tended to report less-negative fat mass effect estimates. Intervention duration showed a positive study-level association that did not reach statistical significance (β = 0.104, 95% CI −0.007 to 0.215; p = 0.066; estimated R^2^ = 42.67%; residual I^2^ = 51.17%), and its potential moderating role therefore remains uncertain. Training frequency showed a statistically significant negative study-level association with the fat mass effect estimate (β = −0.837, 95% CI −1.276 to −0.399; p< 0.001). Although the training-frequency model yielded an estimated R^2^ of 100.00%, with residual I^2^ decreasing to 0.00%, this estimate should be interpreted with particular caution because only eight effect sizes were available, the >3 sessions/week category contained only two effect sizes, and the corresponding between-subgroup comparison was not statistically significant. The estimated proportion of explained variance may therefore be unstable and should not be interpreted as evidence that training frequency completely accounts for the observed heterogeneity. Overall, baseline BMI and training frequency may represent potential study-level moderators of variation in the fat mass effect estimates, but these findings should be regarded as exploratory and hypothesis-generating and should not be interpreted as causal (**Figure 11**).

**Figure 11:**
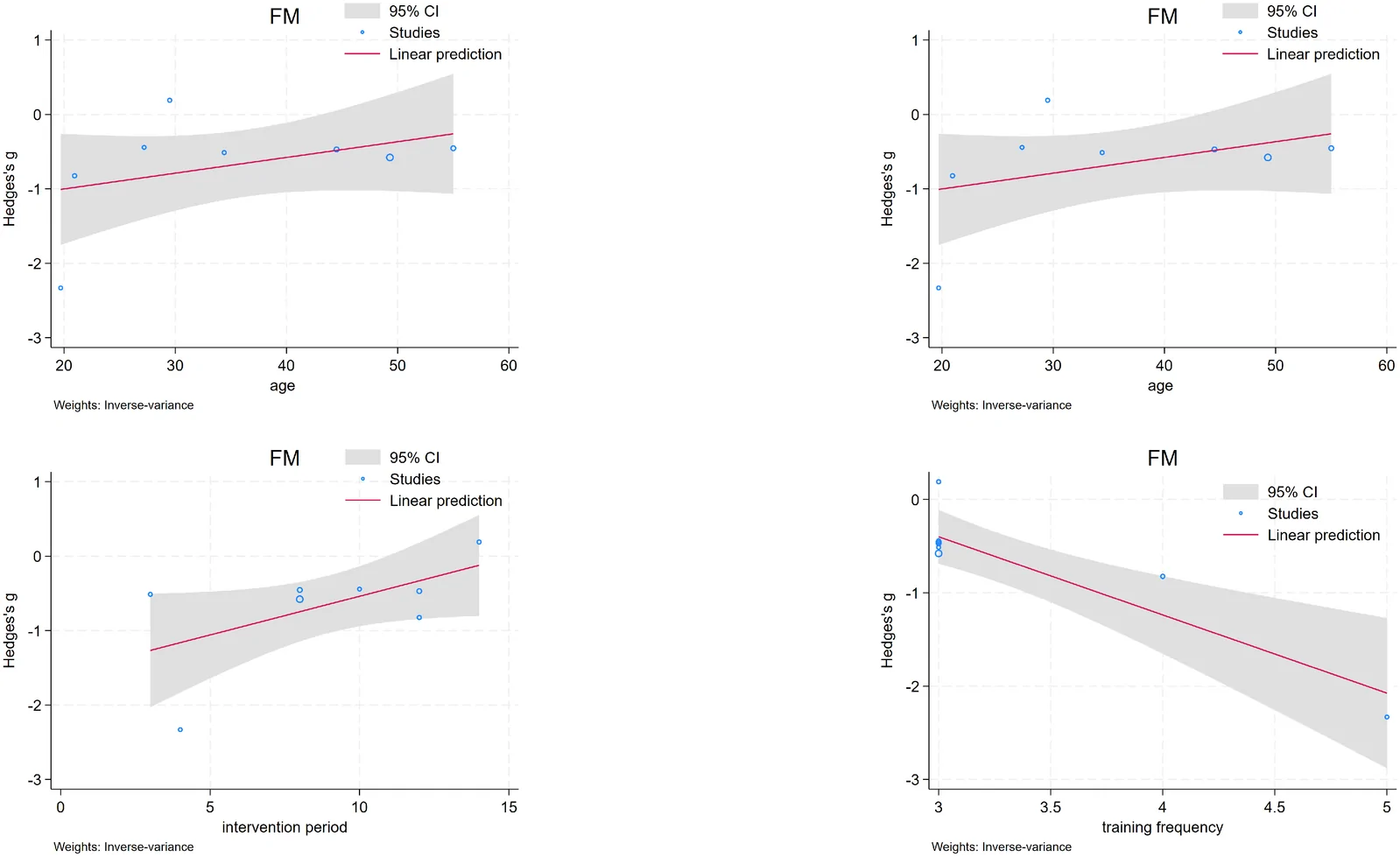
Bubble plots of the univariable meta-regression analyses for fat mass. Panels show the study-level associations between the fat mass effect estimates and mean participant age, baseline BMI, intervention duration, and training frequency. Each dot represents an individual effect size, and the shaded area indicates the 95% confidence interval around the fitted regression line. Effect sizes are expressed as Hedges’ g. Baseline BMI showed a statistically significant positive association with the fat mass effect estimate, whereas training frequency showed a statistically significant negative association. No clear evidence of an association was observed for mean age, and the association with intervention duration did not reach statistical significance. Because only eight effect sizes were available and the distribution of training frequency was limited, these findings should be regarded as exploratory and should not be interpreted as causal.

Leave-one-out sensitivity analysis showed that the pooled fat mass effect estimates ranged from −0.764 to −0.471 after the sequential omission of individual studies, while I^2^ ranged from 0.00% to 70.60% and τ^2^ ranged from 0.0000 to 0.3727. The pooled effect remained negative and statistically significant in all analyses, with corresponding p values ranging from 0.001 to 0.018. Thus, omission of any individual study did not alter the direction or statistical significance of the pooled fat mass effect. Exclusion of Hu et al. (2022) produced the most pronounced change, attenuating the pooled estimate from −0.656 to −0.471 and reducing both I^2^ and τ^2^ to 0. This pattern suggests that Hu et al. (2022) may have contributed substantially to the magnitude of the pooled estimate and to the observed between-study heterogeneity, although it does not establish the study as a definitive source of heterogeneity. Exclusion of Arad et al. (2015) changed the pooled estimate to −0.764 and reduced I^2^ to 58.93%, whereas omission of the remaining studies did not reduce heterogeneity. Omission of any individual study did not alter the direction or statistical interpretation of the pooled FM estimate. However, the magnitude of the effect and the estimated heterogeneity were sensitive to omission of Hu et al. (2022). The magnitude and consistency of the pooled FM effect therefore remain uncertain and should be interpreted cautiously (**Supplementary Table 3**; **Supplementary Figure 19C**).

### 3.4 Effects of HIIT on cardiometabolic health outcomes

#### 3.4.1 Effects of HIIT on maximal or peak oxygen uptake

The forest plot showed that 12 pooled effect sizes involving 370 participants contributed data to the VO_2_max/VO_2_peak outcome (**Figure 12**). The random-effects model indicated that HIIT was associated with a statistically significant improvement in VO_2_max/VO_2_peak compared with control conditions (Hedges’ g = 1.085, 95% CI 0.700 to 1.470; P< 0.001). Moderate-to-high between-study heterogeneity was observed (Q = 30.59, P = 0.001; I^2^ = 66.48%, τ^2^ = 0.2977), indicating that the magnitude of the estimated improvement varied across studies. Accordingly, although the pooled estimate favored HIIT, the magnitude and consistency of the effect should be interpreted cautiously in view of the observed heterogeneity.

**Figure 12:**
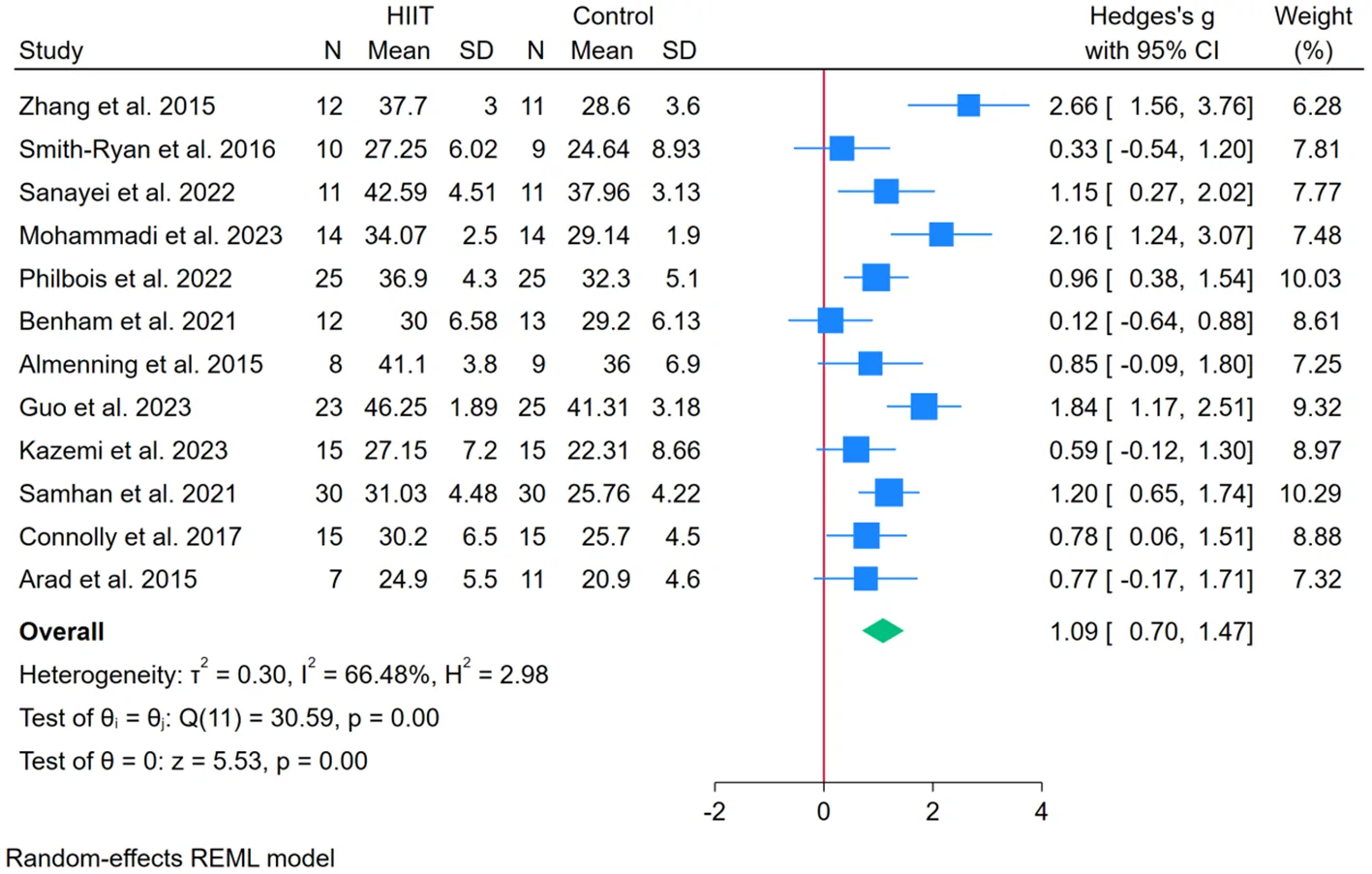
Forest plot of the effects of HIIT on maximal or peak oxygen uptake. Squares represent study-specific effect estimates, with the size of each square proportional to the study weight. Horizontal lines indicate 95% confidence intervals (CIs). The diamond represents the pooled effect estimate, and its width indicates the 95% CI. Effect sizes are expressed as Hedges’ *g* and were pooled using a random-effects restricted maximum likelihood (REML) model. Positive values favor HIIT for improving maximal or peak oxygen uptake.

Subgroup analyses showed that differences in the effects of HIIT on VO_2_max/VO_2_peak according to age stage, baseline BMI, intervention duration, and health status did not reach statistical significance (*p* > 0.05), suggesting that these factors did not show clear evidence of effect modification in the present analysis (**Table 7**). Further inspection of the subgroup results indicated that effect sizes appeared to be relatively greater among participants aged<40 years, those with baseline BMI<30 kg/m^2^, interventions lasting ≤8 weeks, and populations with general overweight/obesity or abnormal body composition. However, none of the corresponding between-subgroup comparisons reached statistical significance (*p* > 0.05); therefore, these findings should be interpreted as exploratory.

Notably, statistically significant between-subgroup differences were observed for training frequency and exercise modality (*p* < 0.05). The effect size appeared to be larger in the subgroup with a training frequency >3 sessions/week (Hedges’ *g* = 2.66, 95% CI 1.56 to 3.76); however, this subgroup included only one effect size, and the stability of this finding is therefore limited. Regarding exercise modality, running-/walking-based HIIT and circuit-/functional-training-based HIIT showed relatively larger effect sizes, whereas cycling-based HIIT showed a comparatively smaller effect size. Given that some subgroups included only a small number of studies, these findings should be interpreted cautiously and do not provide sufficient evidence to conclude that training frequency or exercise modality definitively modifies the effect of HIIT on VO_2_max/VO_2_peak.

Univariable meta-regression analyses did not show clear evidence of a study-level association between the VO_2_max/VO_2_peak effect estimate and mean participant age (β = −0.028, 95% CI −0.061 to 0.005; P = 0.091; estimated R^2^ = 17.05%; residual I^2^ = 61.46%), baseline BMI (β = −0.097, 95% CI −0.242 to 0.048; P = 0.191; estimated R^2^ = 3.93%; residual I^2^ = 64.80%), or intervention duration (β = −0.043, 95% CI −0.110 to 0.024; P = 0.208; estimated R^2^ = 12.69%; residual I^2^ = 63.07%). In contrast, training frequency showed a statistically significant positive study-level association with the VO_2_max/VO_2_peak effect estimate (β = 1.677, 95% CI 0.254 to 3.100; P = 0.021). The training-frequency model accounted for an estimated 39.18% of the between-study variance, with residual I^2^ decreasing to 55.86% and residual τ^2^ to 0.1811. Because positive Hedges’ g values indicate a greater improvement in VO_2_max/VO_2_peak with HIIT, this coefficient suggests that studies using a higher training frequency tended to report larger positive effect estimates. However, this finding should be interpreted with particular caution because only 12 effect sizes were available and the subgroup with a training frequency of >3 sessions/week contained only one effect size. The observed association may therefore be sensitive to the sparse distribution of training frequency and the influence of individual studies. Accordingly, training frequency may represent a potential study-level moderator, but the finding should be regarded as exploratory and hypothesis-generating and should not be interpreted as causal (**Figure 13**).

**Figure 13:**
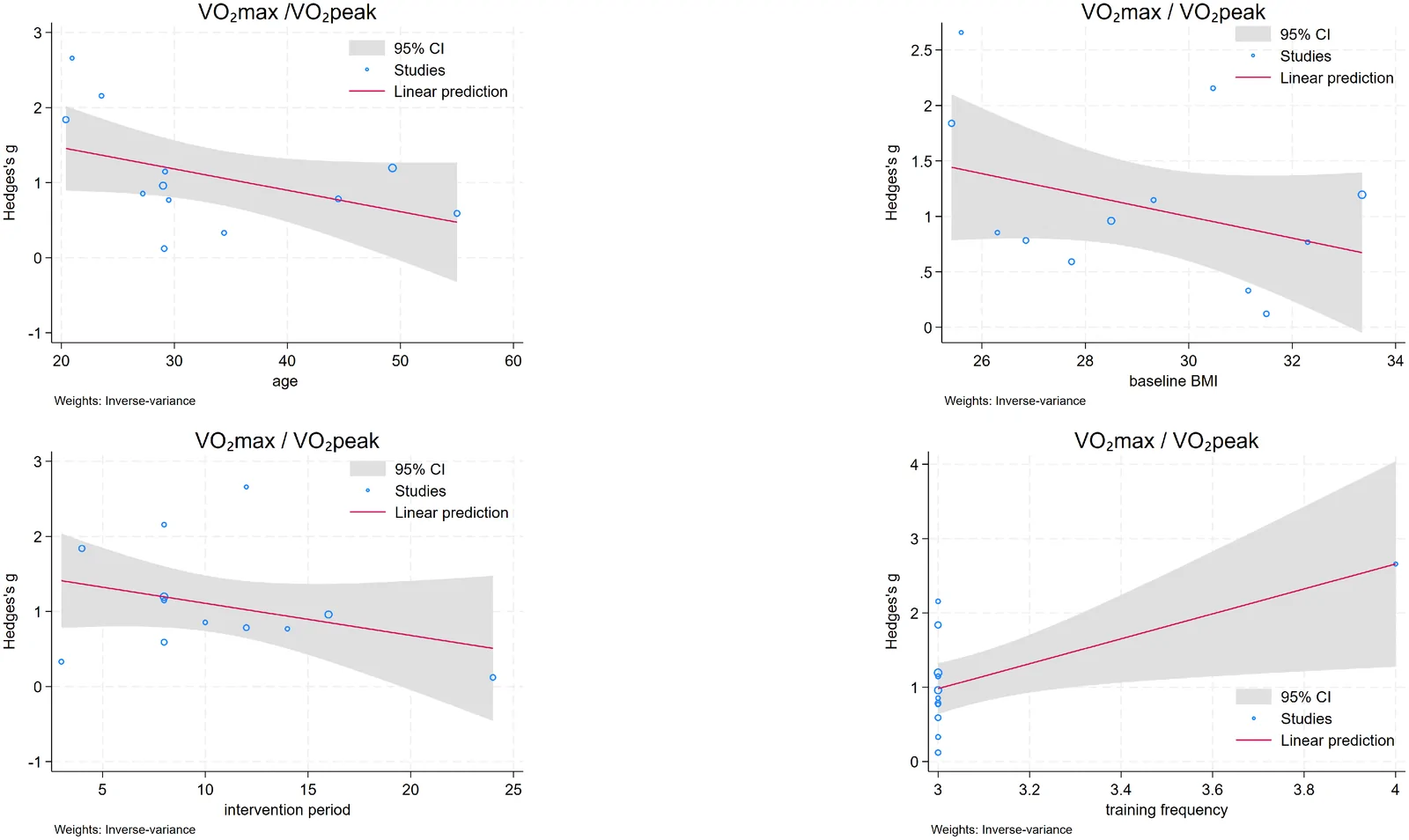
Bubble plots of the univariable meta-regression analyses for maximal or peak oxygen uptake. Panels show the study-level associations between the VO_2_max/VO_2_peak effect estimates and mean participant age, baseline BMI, intervention duration, and training frequency. Each dot represents an individual effect size, and the shaded area indicates the 95% confidence interval around the fitted regression line. Effect sizes are expressed as Hedges’ g. Training frequency showed a statistically significant positive association with the VO_2_max/VO_2_peak effect estimate, whereas no clear evidence of an association was observed for mean age, baseline BMI, or intervention duration. Because only 12 effect sizes were available and the subgroup with a training frequency of >3 sessions/week contained only one effect size, these findings should be regarded as exploratory and should not be interpreted as causal.

Leave-one-out sensitivity analysis showed that the pooled VO_2_max/VO_2_peak effect estimates ranged from 0.981 to 1.172 after the sequential omission of individual studies, while I^2^ ranged from 55.86% to 70.33% and τ^2^ ranged from 0.1811 to 0.3606. The pooled effect remained positive and statistically significant in all analyses, with all corresponding P values below 0.001. Thus, omission of any individual study did not materially alter the direction or statistical interpretation of the pooled VO_2_max/VO_2_peak effect. Exclusion of Zhang et al. (2015) produced the largest reduction in heterogeneity, reducing I^2^ from 66.48% to 55.86% and τ^2^ from 0.2977 to 0.1811, while the pooled estimate decreased from 1.085 to 0.981. I^2^ also decreased to 60.45% and 60.77% after exclusion of Benham et al. (2021) and Mohammadi et al. (2023), respectively. These patterns suggest that the corresponding studies may have contributed to part of the observed heterogeneity, but they do not establish any individual study as a definitive source of heterogeneity. Omission of any individual study did not materially alter the favorable direction or statistical interpretation of the pooled VO_2_max/VO_2_peak estimate. However, moderate heterogeneity remained and the magnitude of the effect varied across analyses. These sensitivity findings do not establish that the effect was precise or consistent across study settings (**Supplementary Table 3**; **Supplementary Figure 19D**).

#### 3.4.2 Effects of HIIT on high-density lipoprotein cholesterol

The forest plot showed that eight pooled effect sizes involving 253 participants contributed data to the HDL-C outcome (**Figure 14**). The random-effects model indicated that HIIT was associated with a statistically significant increase in HDL-C compared with control conditions (Hedges’ g = 0.843, 95% CI 0.240 to 1.446; P = 0.006). Substantial between-study heterogeneity was observed (Q = 35.38, P< 0.001; I^2^ = 80.90%, τ^2^ = 0.5998), indicating considerable variation in the magnitude of the estimated HDL-C response across studies. Accordingly, although the pooled estimate favored HIIT, the magnitude and consistency of the effect should be interpreted cautiously in view of the observed heterogeneity.

**Figure 14:**
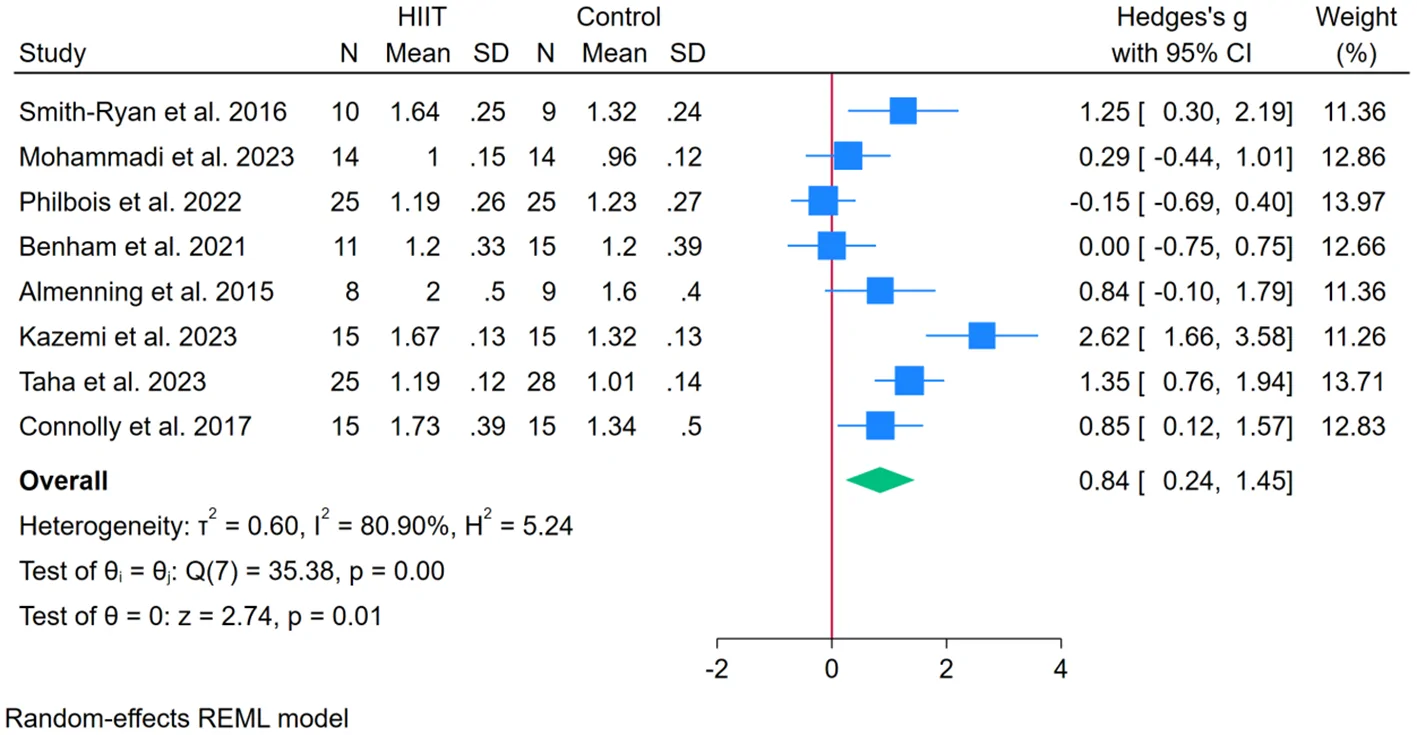
Forest plot of the effects of HIIT on high-density lipoprotein cholesterol. Squares represent study-specific effect estimates, with the size of each square proportional to the study weight. Horizontal lines indicate 95% confidence intervals (CIs). The diamond represents the pooled effect estimate, and its width indicates the 95% CI. Effect sizes are expressed as Hedges’ *g* and were pooled using a random-effects restricted maximum likelihood (REML) model. Positive values favor HIIT for increasing high-density lipoprotein cholesterol.

Subgroup analyses showed that between-subgroup differences in the effects of HIIT on HDL-C according to baseline BMI and intervention duration did not reach statistical significance (*p* > 0.05), suggesting no clear evidence of effect modification by these factors in the present analysis (**Table 8**). Further inspection of the subgroup estimates indicated that the improvement in HDL-C appeared to be relatively greater among participants with BMI ≥30 kg/m^2^ and in interventions lasting ≤8 weeks. However, the corresponding between-subgroup comparisons were not statistically significant (*p* > 0.05). Therefore, these subgroup patterns should be interpreted as exploratory and should not be taken as evidence that baseline BMI or intervention duration definitively modifies the effect of HIIT on HDL-C.

Statistically significant between-subgroup differences were observed for age stage, health status, and exercise modality (*p* < 0.05). The effect estimates appeared to be relatively larger among participants aged ≥40 years, participants with comorbid disease or special health conditions, and HIIT programs involving circuit-based or functional training modalities. However, these findings should be interpreted cautiously, as some subgroups included only a small number of studies; in particular, the circuit-/functional-training-based HIIT subgroup included only one effect size. Therefore, these results do not provide sufficient evidence to conclude that age stage, health status, or exercise modality consistently modifies the effect of HIIT on HDL-C. For training frequency, all included studies were concentrated in the ≤3 sessions/week subgroup, precluding a meaningful between-subgroup comparison.

Univariable meta-regression analysis showed a statistically significant positive study-level association between mean participant age and the HDL-C effect estimate (β = 0.063, 95% CI 0.029 to 0.097; P< 0.001). The age model accounted for an estimated 80.18% of the between-study variance, with residual I^2^ decreasing to 45.11% and residual τ^2^ to 0.1189. Because positive Hedges’ g values indicate a greater increase in HDL-C with HIIT, the positive regression coefficient suggests that studies involving participants with a higher mean age tended to report larger positive HDL-C effect estimates. However, this association should be interpreted cautiously because only eight effect sizes were available, and the estimated proportion of explained variance may be sensitive to the distribution of study-level characteristics and the influence of individual studies. No clear evidence of an association was observed for baseline BMI (β = −0.014, 95% CI −0.266 to 0.239; P = 0.915; estimated R^2^ = 0.00%; residual I^2^ = 83.23%). Intervention duration showed a negative study-level association that did not reach statistical significance (β = −0.081, 95% CI −0.172 to 0.009; P = 0.079; estimated R^2^ = 29.51%; residual I^2^ = 75.13%), and therefore its potential moderating role remains uncertain. Meta-regression according to training frequency was not conducted because the included studies showed insufficient between-study variation in this variable. Overall, mean participant age may represent a potential study-level moderator of variation in the HDL-C effect estimates, but these findings should be regarded as exploratory and hypothesis-generating and should not be interpreted as causal (**Figure 15**).

**Figure 15:**
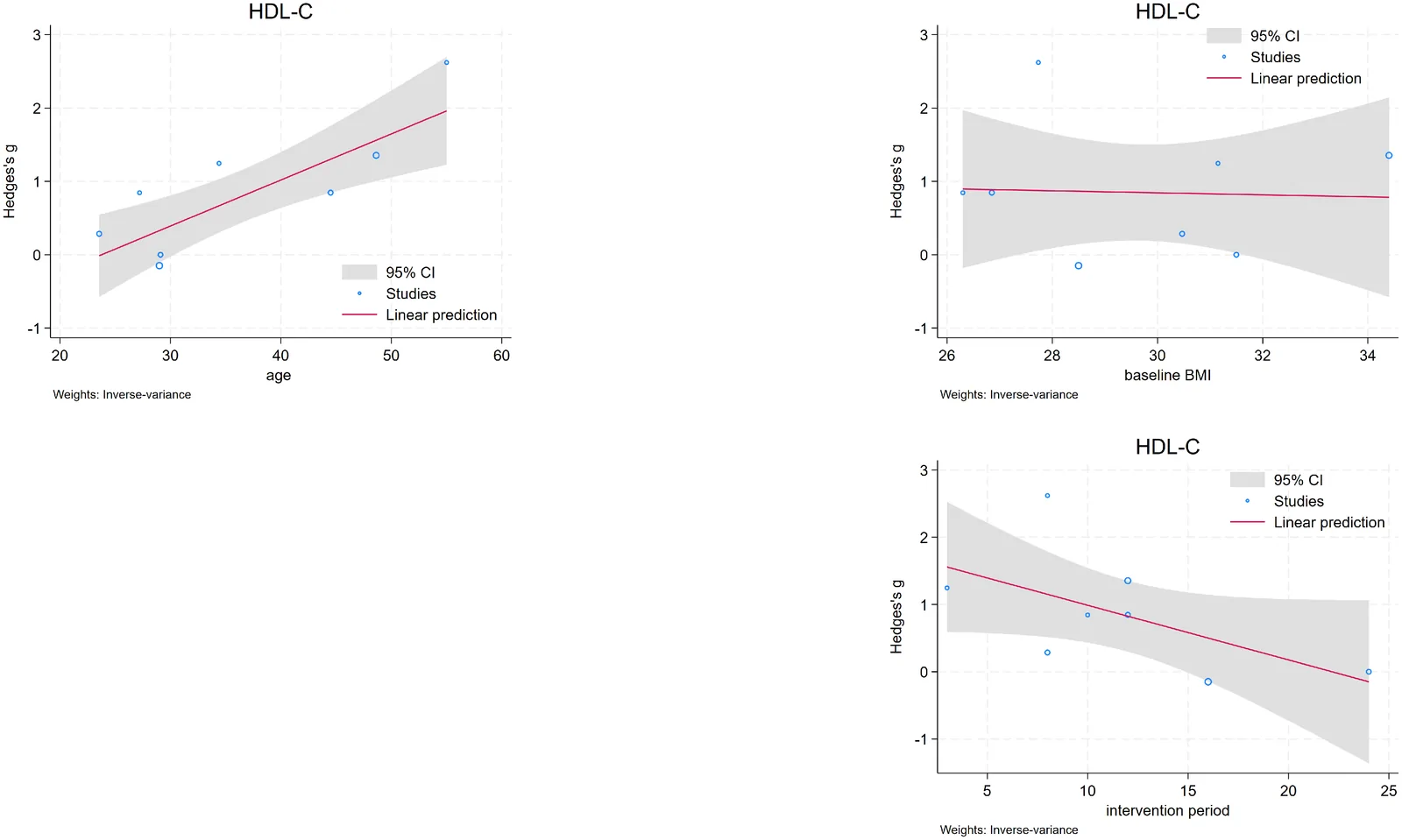
Bubble plots of the univariable meta-regression analyses for high-density lipoprotein cholesterol. Panels show the study-level associations between the HDL-C effect estimates and mean participant age, baseline BMI, and intervention duration. Each dot represents an individual effect size, and the shaded area indicates the 95% confidence interval around the fitted regression line. Effect sizes are expressed as Hedges’ g. Mean participant age showed a statistically significant positive association with the HDL-C effect estimate, whereas no clear evidence of an association was observed for baseline BMI. Intervention duration showed a negative association that did not reach statistical significance. Because only eight effect sizes were available, these findings should be regarded as exploratory and should not be interpreted as causal.

Leave-one-out sensitivity analysis showed that the pooled HDL-C effect estimates ranged from 0.605 to 1.000 after the sequential omission of individual studies, while I^2^ ranged from 65.77% to 84.34% and τ^2^ ranged from 0.2565 to 0.7342. The pooled effect remained positive and statistically significant in all analyses, with corresponding P values ranging from 0.001 to 0.027. Thus, omission of any individual study did not materially alter the direction or statistical interpretation of the pooled HDL-C effect. Exclusion of Kazemi et al. (2023) produced the largest change, reducing the pooled estimate from 0.843 to 0.605, I^2^ from 80.90% to 65.77%, and τ^2^ from 0.5998 to 0.2565. This pattern suggests that the study may have contributed to part of the observed heterogeneity and to the magnitude of the pooled estimate, but it does not establish the study as a definitive source of heterogeneity. Smaller changes in the magnitude of the pooled effect were observed after omission of Philbois et al. (2022) and Benham et al. (2021), without meaningful improvement in heterogeneity or alteration of the statistical interpretation. Omission of any individual study did not materially alter the direction or statistical interpretation of the pooled HDL-C estimate. However, the effect magnitude varied and substantial residual heterogeneity remained. The magnitude and consistency of the HDL-C effect therefore remain uncertain and should be interpreted cautiously (**Supplementary Table 3**; **Supplementary Figure 19E**).

#### 3.4.3 Effects of HIIT on low-density lipoprotein cholesterol

The forest plot showed that eight pooled effect sizes involving 253 participants contributed data to the LDL-C outcome (**Figure 16**). The random-effects model indicated that HIIT was associated with a statistically significant reduction in LDL-C compared with control conditions (Hedges’ g = −0.780, 95% CI −1.293 to −0.266; P = 0.003). Moderate-to-high between-study heterogeneity was observed (Q = 29.41, P< 0.001; I^2^ = 74.16%, τ^2^ = 0.4011), indicating that the magnitude of the estimated LDL-C-lowering effect varied across studies. Accordingly, although the pooled estimate favored HIIT, its magnitude should be interpreted cautiously in view of the observed heterogeneity.

**Figure 16:**
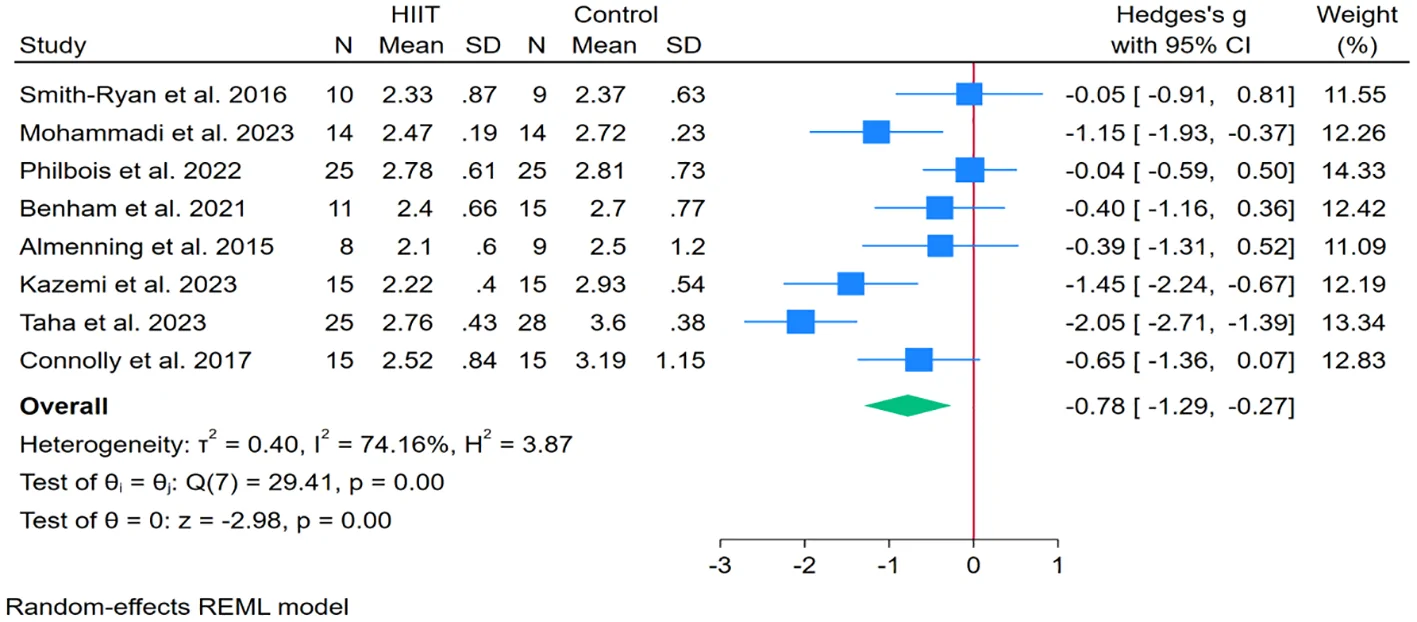
Forest plot of the effects of HIIT on low-density lipoprotein cholesterol. Squares represent study-specific effect estimates, with the size of each square proportional to the study weight. Horizontal lines indicate 95% confidence intervals (CIs). The diamond represents the pooled effect estimate, and its width indicates the 95% CI. Effect sizes are expressed as Hedges’ *g* and were pooled using a random-effects restricted maximum likelihood (REML) model. Negative values favor HIIT for reducing low-density lipoprotein cholesterol.

Subgroup analyses showed that between-subgroup differences in the effects of HIIT on LDL-C according to baseline BMI, intervention duration, and exercise modality did not reach statistical significance (*p* > 0.05), suggesting no clear evidence of effect modification by these factors in the present analysis (**Table 9**). Further inspection of the subgroup estimates indicated that reductions in LDL-C appeared to be relatively more pronounced among participants with BMI ≥30 kg/m^2^, interventions lasting ≤8 weeks, and HIIT programs involving circuit-based or functional training modalities. However, the corresponding between-subgroup comparisons were not statistically significant (*p* > 0.05). Therefore, these subgroup patterns should be interpreted as exploratory.

Statistically significant between-subgroup differences were observed for age stage and health status. Specifically, the LDL-C–lowering effect appeared to be more pronounced among participants aged ≥40 years than among those aged<40 years (*p* = 0.03), and a relatively larger effect estimate was also observed among participants with comorbid disease or special health conditions (*p* < 0.01). However, these findings should be interpreted cautiously because some subgroups included only a small number of studies. In particular, the circuit-/functional-training-based HIIT subgroup included only one effect size. For training frequency, all included studies were concentrated in the ≤3 sessions/week subgroup, which precluded a meaningful between-subgroup comparison.

Univariable meta-regression yielded a negative coefficient for mean participant age in relation to the LDL-C effect estimate (β = −0.04023, 95% CI −0.08047 to 0.00001; P = 0.0501). The age model accounted for an estimated 39.95% of the between-study variance, although considerable residual heterogeneity remained (residual I^2^ = 63.08%). Because the 95% CI marginally crossed zero and the P value did not meet the prespecified threshold of P< 0.05, this association was not considered statistically significant. Given that only eight effect sizes were available, the finding should be regarded as exploratory and should not be interpreted as evidence of a causal moderating effect. No clear evidence of an association was observed for baseline BMI (β = −0.118, 95% CI −0.308 to 0.071; P = 0.222; estimated R^2^ = 12.30%; residual I^2^ = 71.24%) or intervention duration (β = 0.019, 95% CI −0.074 to 0.112; P = 0.690; estimated R^2^ = 0.00%; residual I^2^ = 77.03%). Meta-regression according to training frequency was not conducted because the included studies showed insufficient between-study variation in this variable. Given that only eight effect sizes were available, these study-level findings should be regarded as exploratory and hypothesis-generating and should not be interpreted as evidence of a causal moderating effect (**Figure 17**).

**Figure 17:**
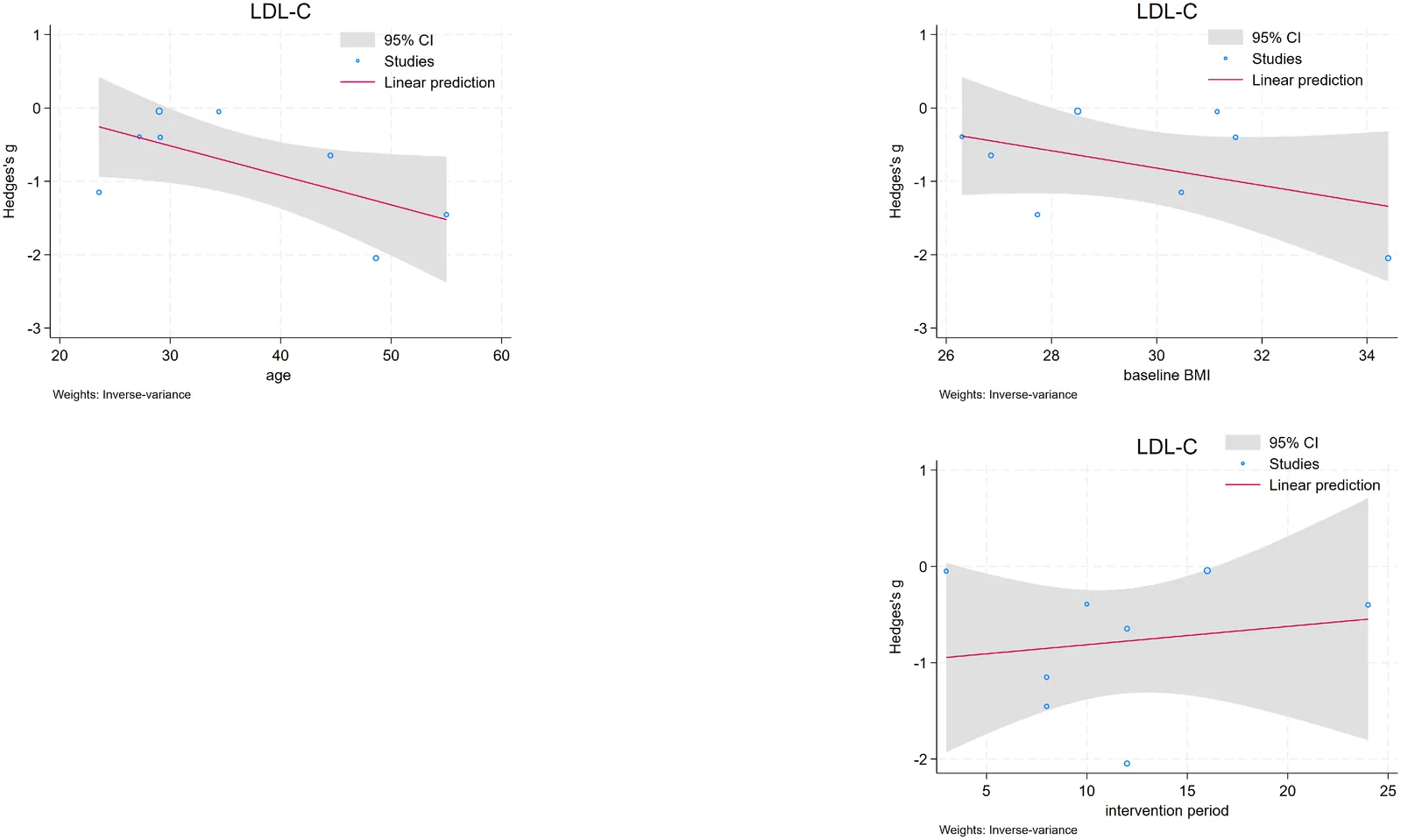
Bubble plots of the univariable meta-regression analyses for low-density lipoprotein cholesterol. Panels show the study-level associations between the LDL-C effect estimates and mean participant age, baseline BMI, and intervention duration. Each dot represents an individual effect size, and the shaded area indicates the 95% confidence interval around the fitted regression line. Effect sizes are expressed as Hedges’ g. The fitted relationship for mean age was negative, although the confidence interval marginally included the null value and the statistical evidence remained inconclusive. No clear evidence of an association was observed for baseline BMI or intervention duration. Because only eight effect sizes were available, these findings should be regarded as exploratory and should not be interpreted as causal.

Leave-one-out sensitivity analysis showed that the pooled LDL-C effect estimates ranged from −0.904 to −0.575 after the sequential omission of individual studies, while I^2^ ranged from 50.75% to 77.45% and τ^2^ ranged from 0.1494 to 0.4844. The direction of the pooled effect remained negative, and the corresponding P values ranged from 0.001 to 0.015; thus, the statistical interpretation of the LDL-C finding was not materially altered by omission of any individual study. Exclusion of Taha et al. (2023) produced the largest change, reducing I^2^ from 74.16% to 50.75% and τ^2^ from 0.4011 to 0.1494, while attenuating the pooled effect estimate from −0.780 to −0.575. This pattern suggests that the study may have contributed to part of the observed heterogeneity and to the magnitude of the pooled estimate, but it does not establish the study as a definitive source of heterogeneity. Smaller changes in the pooled effect were observed after exclusion of Philbois et al. (2022) and Smith-Ryan et al. (2016), without altering the direction or statistical significance of the result. Omission of any individual study did not materially alter the direction or statistical interpretation of the pooled LDL-C estimate. However, the effect magnitude varied and moderate-to-high residual heterogeneity remained. The magnitude and consistency of the LDL-C effect therefore remain uncertain and should be interpreted cautiously (**Supplementary Table 3**; **Supplementary Figure 19F**).

#### 3.4.4 Effects of HIIT on total cholesterol

The forest plot showed that nine pooled effect sizes involving 276 participants contributed data to the TC outcome (**Figure 18**). The random-effects model yielded a pooled effect estimate of Hedges’ g = −0.346 (95% CI −0.701 to 0.009; P = 0.056). Although the point estimate favored HIIT for reducing TC, the confidence interval included the line of no effect, and the pooled result did not reach statistical significance. Moderate between-study heterogeneity was observed (Q = 17.49, P = 0.025; I^2^ = 54.23%, τ^2^ = 0.1569). Therefore, the current evidence is insufficient to conclude that HIIT produces a clear reduction in TC, and the variability across studies warrants further exploration.

**Figure 18:**
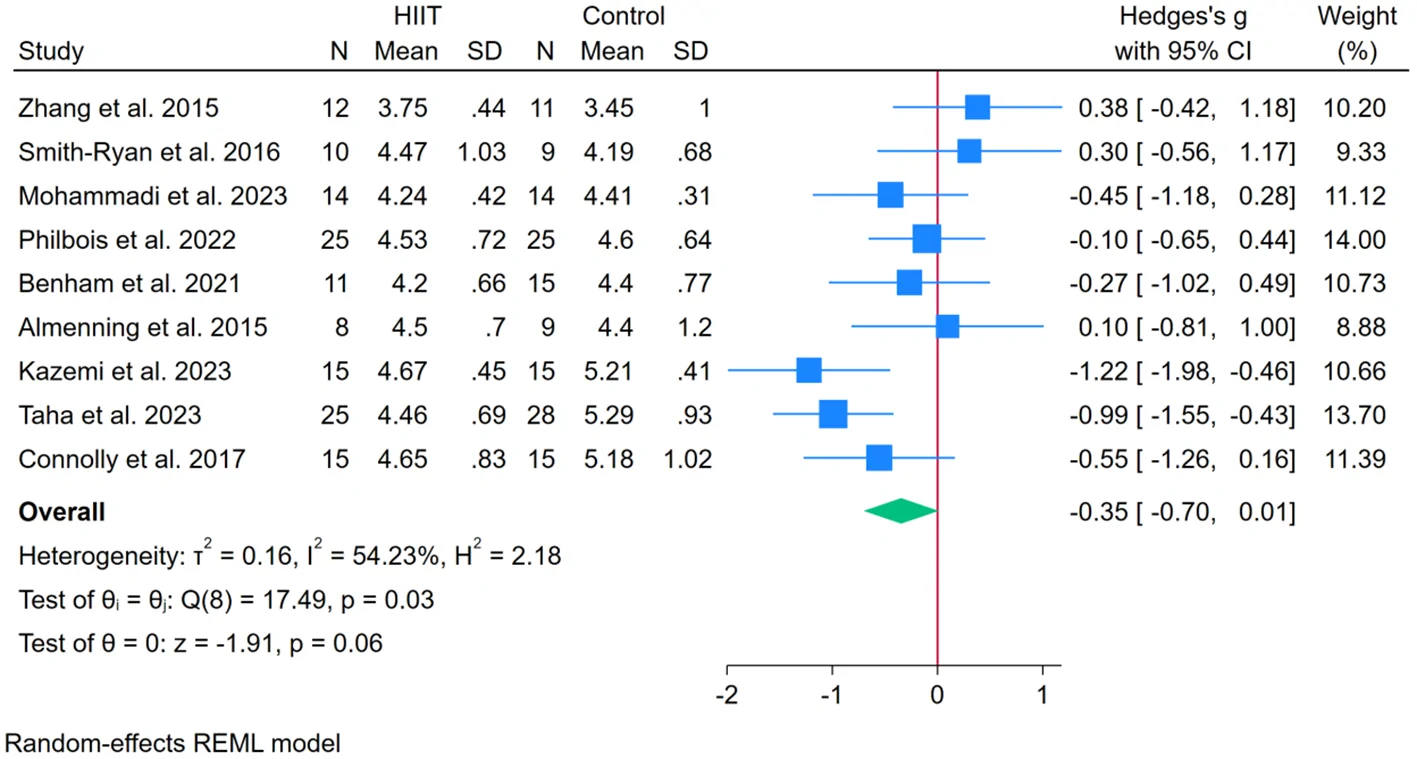
Forest plot of the effects of HIIT on total cholesterol. Squares represent study-specific effect estimates, with the size of each square proportional to the study weight. Horizontal lines indicate 95% confidence intervals (CIs). The diamond represents the pooled effect estimate, and its width indicates the 95% CI. Effect sizes are expressed as Hedges’ *g* and were pooled using a random-effects restricted maximum likelihood (REML) model. Negative values favor HIIT for reducing total cholesterol.

Subgroup analyses showed that between-subgroup differences in the effects of HIIT on TC according to baseline BMI, intervention duration, and training frequency did not reach statistical significance (*p* > 0.05), suggesting no clear evidence of effect modification by these factors in the present analysis (**Table 10**). Further inspection of the subgroup estimates indicated that reductions in TC appeared to be relatively more pronounced among participants with BMI ≥30 kg/m^2^, interventions lasting ≤8 weeks, and interventions with a training frequency ≤3 sessions/week. However, the corresponding between-subgroup comparisons were not statistically significant (*p* > 0.05). Therefore, these subgroup patterns should be interpreted as exploratory.

Statistically significant between-subgroup differences were observed for age stage, health status, and exercise modality. Specifically, the TC-lowering effect appeared to be more pronounced among participants aged ≥40 years (Hedges’ *g* = −0.92, 95% CI −1.30 to −0.54), and a relatively larger effect estimate was also observed among participants with comorbid disease or special health conditions (Hedges’ *g* = −1.07, 95% CI −1.52 to −0.62). Regarding exercise modality, circuit-/functional-training-based HIIT showed a relatively larger effect estimate (Hedges’ *g* = −1.22, 95% CI −1.98 to −0.46). However, these between-subgroup differences should be interpreted cautiously because some subgroups included only a small number of studies; in particular, the circuit-/functional-training-based HIIT subgroup included only one effect size.

Univariable meta-regression analysis showed a statistically significant study-level association between mean participant age and the TC effect estimate (β = −0.038, 95% CI −0.059 to −0.017; P< 0.001). The age model yielded an estimated R^2^ of 100.00%, with residual I^2^ decreasing to 0.00%. Because negative Hedges’ g values indicate a greater reduction in TC with HIIT, the negative regression coefficient suggests that studies involving participants with a higher mean age tended to report more negative TC effect estimates. However, the estimated proportion of explained between-study variance should be interpreted with particular caution because the analysis included only nine effect sizes and may be sensitive to the distribution of study-level characteristics and the influence of individual studies. No clear evidence of an association was observed for baseline BMI (β = −0.071, 95% CI −0.194 to 0.052; P = 0.258), intervention duration (β = 0.001, 95% CI −0.069 to 0.071; P = 0.985), or training frequency (β = 0.813, 95% CI −0.291 to 1.916; P = 0.149). Accordingly, mean age may represent a potential study-level moderator of variation in the TC effect estimates, but the finding should be regarded as exploratory and hypothesis-generating rather than causal (**Figure 19**).

**Figure 19:**
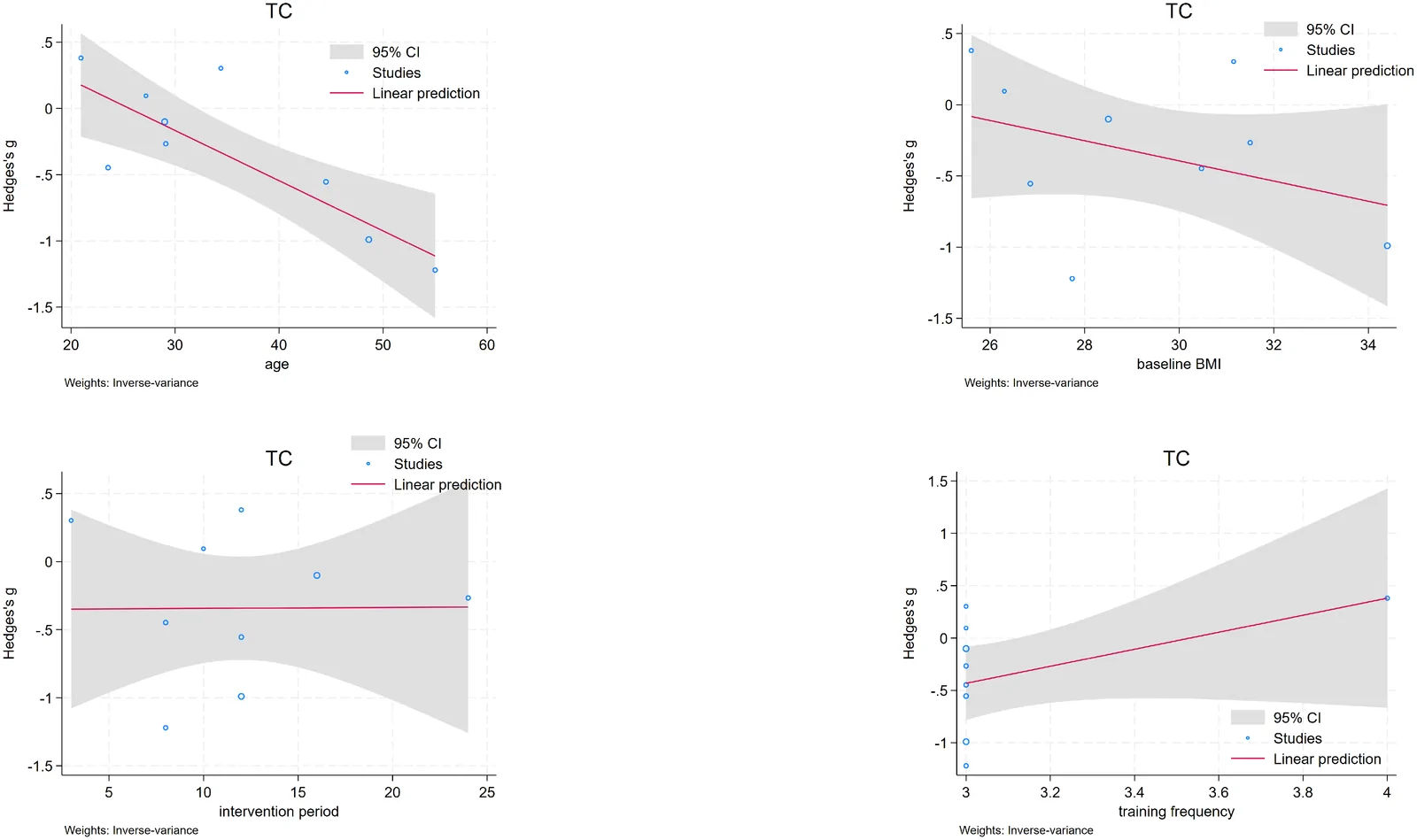
Bubble plots of the univariable meta-regression analyses for total cholesterol. Panels show the study-level associations between the TC effect estimates and mean participant age, baseline BMI, intervention duration, and training frequency. Each dot represents an individual effect size, and the shaded area indicates the 95% confidence interval around the fitted regression line. Effect sizes are expressed as Hedges’ g. Mean participant age showed a statistically significant negative association with the TC effect estimate, whereas no clear evidence of an association was observed for baseline BMI, intervention duration, or training frequency. Because only nine effect sizes were available, these findings should be regarded as exploratory and should not be interpreted as causal.

Leave-one-out sensitivity analysis showed that the pooled TC effect estimates ranged from −0.432 to −0.246 after the sequential omission of individual studies, while I^2^ ranged from 41.53% to 60.06% and τ^2^ ranged from 0.1014 to 0.1987. Exclusion of Taha et al. (2023) and Kazemi et al. (2023) reduced I^2^ from 54.23% to 41.53% and 43.86%, respectively, suggesting that these studies may have contributed to part of the observed between-study heterogeneity. However, these changes do not establish either study as a definitive source of heterogeneity. The pooled effect remained negative in all leave-one-out analyses. Nevertheless, exclusion of Zhang et al. (2015), Smith-Ryan et al. (2016), or Almenning et al. (2015) changed the pooled estimate from non-significant to statistically significant, with corresponding P values of 0.015, 0.026, and 0.045, respectively. Because the primary pooled result was close to the conventional threshold for statistical significance, these changes are more appropriately interpreted as sensitivity of the statistical significance to the omission of individual studies rather than as a reversal of the estimated effect. The direction of the TC estimate remained negative across the leave-one-out analyses, but its statistical significance was sensitive to omission of individual studies. The magnitude and statistical interpretation of the pooled TC effect therefore remain uncertain and should be interpreted cautiously (**Supplementary Table 3**; **Supplementary Figure 19G**).

#### 3.4.5 Effects of HIIT on triglycerides

The forest plot showed that nine pooled effect sizes involving 276 participants contributed data to the TG outcome (**Figure 20**). The random-effects model indicated that, compared with control conditions, the reduction in TG associated with HIIT did not reach statistical significance, with a pooled effect size of Hedges’ *g* = −0.24, 95% CI −0.69 to 0.21; *p* = 0.29. The heterogeneity test indicated moderate-to-high between-study heterogeneity (Q = 27.88, P< 0.01; I^2^ = 71.09%, τ^2^ = 0.3294). These findings suggest that HIIT may be associated with a potential trend toward lower TG levels in women; however, the overall effect remains uncertain and the statistical evidence is insufficient.

**Figure 20:**
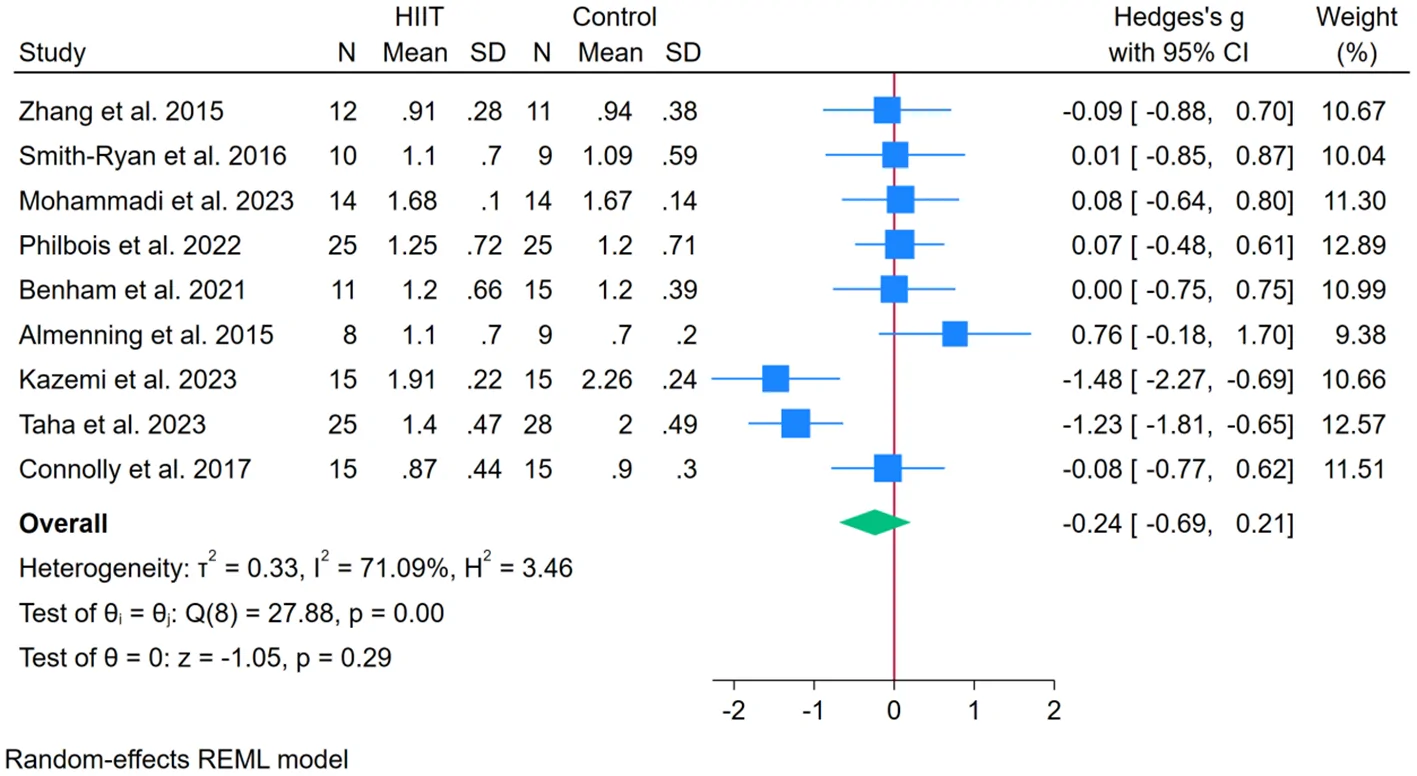
Forest plot of the effects of HIIT on triglycerides. Squares represent study-specific effect estimates, with the size of each square proportional to the study weight. Horizontal lines indicate 95% confidence intervals (CIs). The diamond represents the pooled effect estimate, and its width indicates the 95% CI. Effect sizes are expressed as Hedges’ *g* and were pooled using a random-effects restricted maximum likelihood (REML) model. Negative values favor HIIT for reducing triglycerides.

Subgroup analyses showed that between-subgroup differences in the effects of HIIT on TG according to baseline BMI, intervention duration, and training frequency did not reach statistical significance (*p* > 0.05), suggesting no clear evidence of effect modification by these factors in the present analysis (**Table 11**). Further inspection of the subgroup estimates indicated that reductions in TG appeared to be relatively more pronounced among participants with BMI ≥30 kg/m^2^, interventions lasting ≤8 weeks, and interventions with a training frequency ≤3 sessions/week. However, the corresponding between-subgroup comparisons were not statistically significant (*p* > 0.05). Therefore, these subgroup patterns should be interpreted as exploratory.

Statistically significant between-subgroup differences were observed for age stage, health status, and exercise modality. Specifically, the TG-lowering effect appeared to be more pronounced among participants aged ≥40 years (Hedges’ *g* = −0.93, 95% CI −1.76 to −0.09), and a relatively larger effect estimate was also observed among participants with comorbid disease or special health conditions (Hedges’ *g* = −1.32, 95% CI −1.79 to −0.85). Regarding exercise modality, circuit-/functional-training-based HIIT showed a relatively larger effect estimate (Hedges’ *g* = −1.48, 95% CI −2.27 to −0.69). However, these between-subgroup differences should be interpreted cautiously because some subgroups included only a small number of studies; in particular, the circuit-/functional-training-based HIIT subgroup included only one effect size. Therefore, these findings do not provide sufficient evidence to infer a stable effect-modifying role.

Univariable meta-regression analysis showed that mean participant age was significantly associated with the effect size of HIIT on TG (β = −0.047, 95% CI −0.071 to −0.024; P< 0.001). The age model accounted for 90.94% of the estimated between-study variance (R^2^ = 90.94%), and the residual I^2^ decreased to 18.06%. Because negative Hedges’ g values indicate a greater reduction in TG with HIIT, the negative regression coefficient suggests that studies involving older participants tended to report more pronounced TG-lowering effects. In contrast, baseline BMI (β = −0.092, 95% CI −0.250 to 0.066; P = 0.254), intervention duration (β = 0.015, 95% CI −0.072 to 0.101; P = 0.742), and training frequency (β = 0.168, 95% CI −1.374 to 1.710; P = 0.831) were not significantly associated with the TG effect estimates. However, because only nine effect sizes were available, these study-level associations were considered exploratory and should not be interpreted as causal (**Figure 21**).

**Figure 21:**
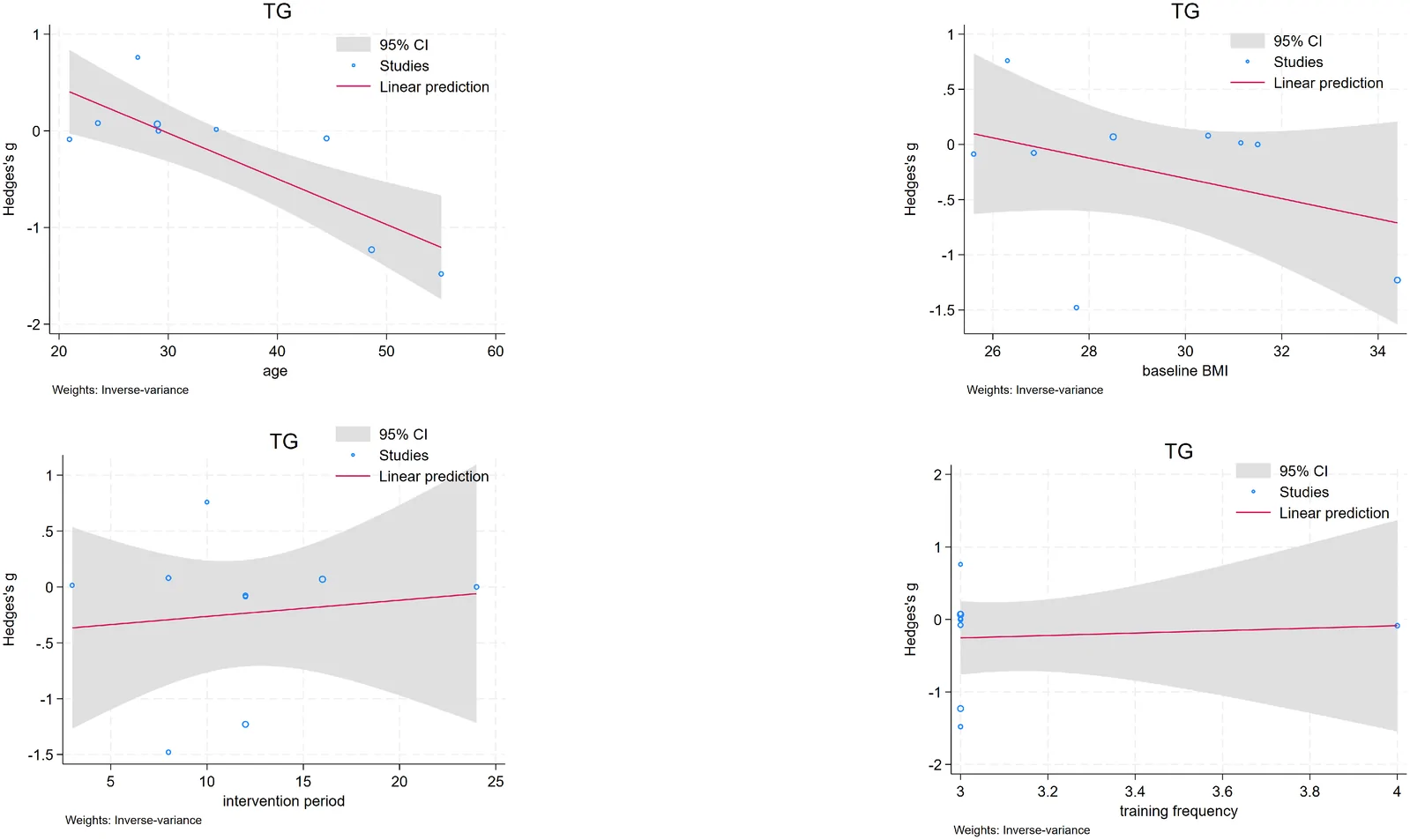
Bubble plots of the univariable meta-regression analyses for triglycerides. Panels show the associations between the effect size for TG and age, baseline BMI, intervention duration, and training frequency. Each dot represents an individual effect size, and the shaded area indicates the 95% confidence interval (CI) around the fitted regression line. Effect sizes are expressed as Hedges’ *g*. The fitted lines suggest that age may be associated with the effect size of HIIT on TG, whereas no clear evidence of an association was observed for baseline BMI, intervention duration, or training frequency. These findings should be interpreted cautiously because of the limited number of effect sizes included in the meta-regression analysis.

Leave-one-out sensitivity analysis showed that the pooled effect estimates ranged from −0.344 to −0.099 after the sequential exclusion of individual studies, while I^2^ ranged from 56.45% to 74.85% and τ^2^ ranged from 0.1855 to 0.3940. Exclusion of Taha et al. (2023) reduced I^2^ from 71.09% to 56.45%, whereas exclusion of Kazemi et al. (2023) reduced I^2^ to 59.68%, suggesting that these two studies may have contributed to the observed heterogeneity. Nevertheless, exclusion of any individual study did not change the negative direction or the non-significant statistical interpretation of the pooled TG effect; all recalculated 95% CIs crossed the line of no effect, and the corresponding P values ranged from 0.128 to 0.628. The non-significant statistical interpretation of the TG result was not altered by omission of any individual study. However, the pooled estimate remained imprecise and moderate residual heterogeneity persisted. The analysis therefore does not provide precise evidence regarding the effect of HIIT on TG (**Supplementary Table 3**; **Supplementary Figure 19H**).

### 3.5 Sensitivity analyses

Four complementary sets of sensitivity analyses were conducted. After studies judged to be at overall high risk of bias were excluded, the direction and statistical interpretation of the primary findings remained unchanged for all nine outcomes. BW, BMI, BF%, FM, VO_2_max/VO_2_peak, HDL-C, and LDL-C retained statistically significant effects in the same direction as the primary analyses, whereas TC and TG remained statistically non-significant. Between-study heterogeneity was substantially reduced for BF% and FM but was not consistently reduced for the other outcomes. These findings indicate that the primary statistical interpretations were not solely dependent on studies judged to be at high risk of bias; however, they do not establish that the estimated effect magnitudes or heterogeneity statistics were fully robust (**Supplementary Table 7**; **Supplementary Figures 20A–I**).

In the population-definition sensitivity analysis, exclusion of Hu et al. (2022) did not alter the direction or statistical significance of the pooled estimates for BW, BMI, BF%, or FM. Heterogeneity was markedly reduced for BF% and FM, changed only slightly for BMI, and increased modestly for BW. These findings suggest that inclusion of the normal-weight-obesity population may have contributed to part of the between-study variability in adiposity-related outcomes, particularly BF% and FM. Nevertheless, the direction and statistical interpretation of the four body-composition outcomes were not dependent on this single study (**Supplementary Table 8**; **Supplementary Figures 21A–D**).

In the broader population-eligibility sensitivity analysis, studies for which conventional overweight or obesity eligibility could not be clearly verified were excluded. The pooled directions remained favorable to HIIT for BW, BMI, BF%, FM, and VO_2_max/VO_2_peak; however, statistical significance was not retained for BW, BMI, or BF%, whereas the favorable effects for FM and VO_2_max/VO_2_peak remained statistically significant. TC and TG remained statistically non-significant, although the TC point estimate changed direction and only two studies remained for each of these outcomes. HDL-C and LDL-C were not quantitatively pooled because only one study remained for each outcome after exclusion. These findings indicate that several estimates, particularly those for BW, BMI, and BF%, were sensitive to population-eligibility assumptions and should be interpreted cautiously (**Supplementary Table 10**).

The leave-one-out sensitivity analyses produced broadly consistent findings. Sequential omission of individual studies did not alter the direction or statistical interpretation of the pooled estimates for BMI, BF%, FM, VO_2_max/VO_2_peak, HDL-C, LDL-C, or TG. In contrast, the statistical significance of the pooled TC estimate varied according to the study omitted, indicating that the TC result was comparatively less stable. For several outcomes, omission of individual studies nevertheless produced appreciable changes in the magnitude of the pooled effect or in the heterogeneity statistics. Therefore, the leave-one-out analyses support relative stability in the direction and statistical interpretation of most findings but do not demonstrate that the estimated effect magnitudes were precise or that between-study heterogeneity was consistently resolved (**Supplementary Table 3**; **Supplementary Figures 19A–H**).

### 3.6 Assessment of small-study effects and publication bias

Funnel plots were visually inspected for all nine outcomes (**Figure 22**). Some plots showed mild departures from symmetry; however, interpretation was limited by the relatively small number of studies contributing to several outcomes.

**Figure 22:**
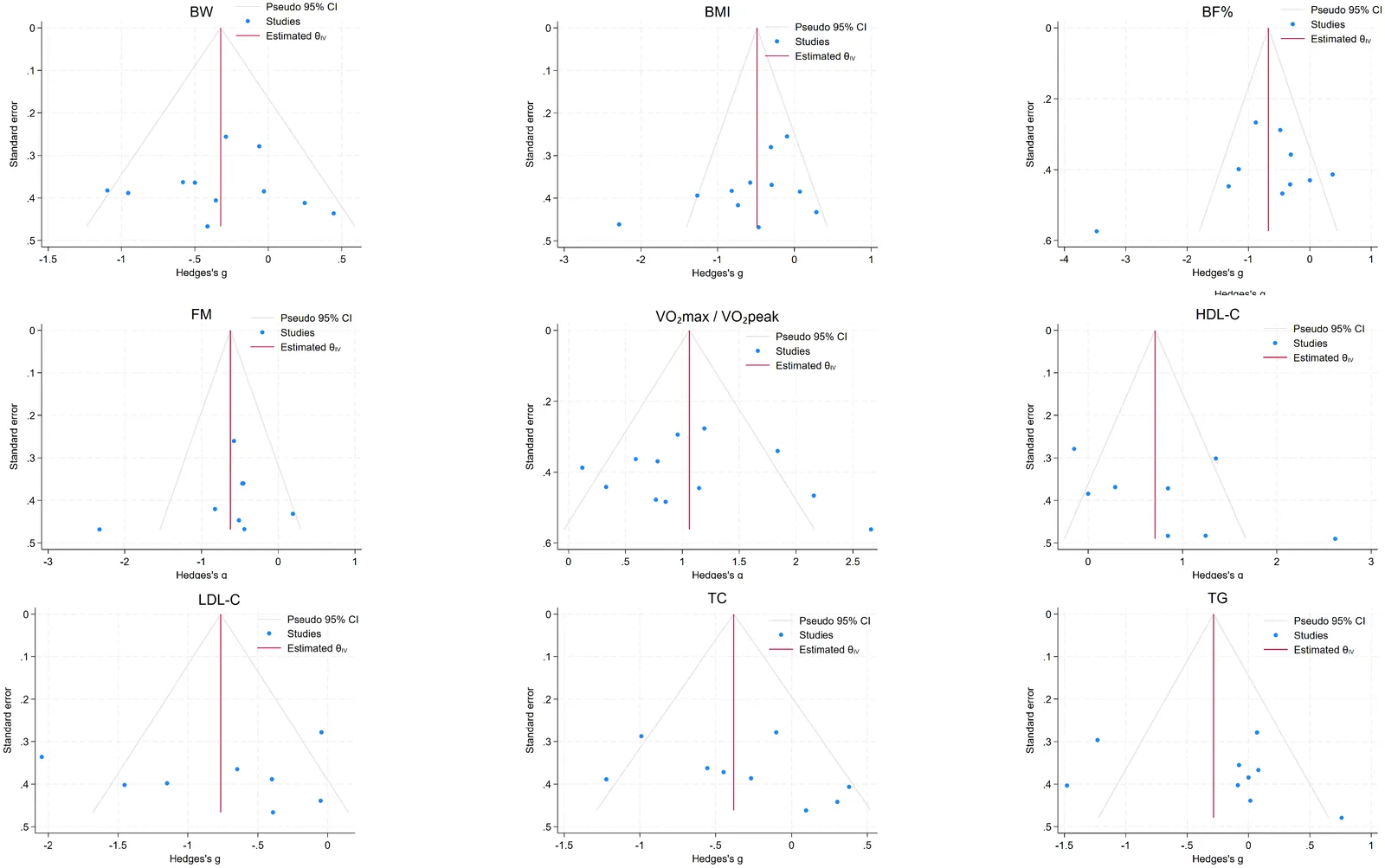
Funnel plots for the assessment of small-study effects and potential publication bias. Funnel plots are presented for body weight (BW), body mass index (BMI), body fat percentage (BF%), fat mass (FM), maximal or peak oxygen uptake (VO_2_max/VO_2_peak), high-density lipoprotein cholesterol (HDL-C), low-density lipoprotein cholesterol (LDL-C), total cholesterol (TC), and triglycerides (TG). Each dot represents an individual effect estimate. The vertical line indicates the pooled effect estimate, and the diagonal lines represent the pseudo 95% confidence limits. Visual inspection did not identify marked asymmetry, although the small number of studies for several outcomes limited the reliability of this assessment. Egger’s tests for outcomes with fewer than 10 independent effect estimates were treated as exploratory and were not interpreted as evidence that publication bias was absent.

For outcomes with at least 10 independent effect estimates, Egger’s regression tests did not show statistically detectable small-study effects for BW (k = 11, P = 0.9944), BMI (k = 11, P = 0.1562), BF% (k = 10, P = 0.1155), or VO_2_max/VO_2_peak (k = 12, P = 0.3540). These findings did not provide clear statistical evidence of funnel-plot asymmetry, but they should not be interpreted as demonstrating that publication bias was absent.

FM, HDL-C, LDL-C, TC, and TG included only 8–9 independent effect estimates. The corresponding Egger’s test results—FM (k = 8, P = 0.5117), HDL-C (k = 8, P = 0.1014), LDL-C (k = 8, P = 0.8404), TC (k = 9, P = 0.2116), and TG (k = 9, P = 0.3273)—were therefore treated as descriptive and exploratory because the statistical power to detect funnel-plot asymmetry was limited.

Overall, no outcome showed compelling evidence of marked funnel-plot asymmetry based on the available data. Nevertheless, the possibility of publication bias could not be excluded, particularly for outcomes based on fewer than 10 studies. These limitations were incorporated into the outcome-specific GRADE judgements rather than interpreting non-significant Egger’s tests as evidence that publication bias was absent.

### 3.7 GRADE assessment of the certainty of evidence

The certainty of evidence was reassessed after updating the risk-of-bias evaluation using RoB 2 and revising the criteria used to judge imprecision and publication bias. Evidence from randomized controlled trials initially started at high certainty.

Risk of bias was rated as serious for all outcomes because the body of evidence for each outcome included trials with some concerns or high overall RoB 2 judgements, primarily related to the randomization process, missing outcome data, and selection of the reported result. These judgements were based on the methodological limitations of the trials contributing to each outcome and were not based solely on the inability to blind participants and exercise personnel.

BW was not downgraded for inconsistency because between-study heterogeneity was low and the study estimates were generally consistent in direction. BMI, BF%, FM, VO_2_max/VO_2_peak, HDL-C, LDL-C, TC, and TG were downgraded by one level because moderate-to-substantial heterogeneity remained and was not explained with sufficient certainty. No outcome was downgraded for indirectness because the populations, HIIT interventions, comparators without structured exercise, and measured outcomes were directly aligned with the prespecified review question.

BW, BMI, BF%, FM, VO_2_max/VO_2_peak, HDL-C, and LDL-C were downgraded by one level for imprecision because the total information size was limited and the pooled confidence intervals included materially different magnitudes of effect. TC and TG were downgraded by two levels because their confidence intervals crossed the line of no effect and included both potentially meaningful benefit and little or no effect; the TG confidence interval also included a possible effect in the opposite direction.

No outcome was downgraded for publication bias after considering the scope of the electronic database and supplementary citation searches, study numbers and sizes, funnel-plot distributions, and Egger’s tests where sufficiently interpretable. However, this judgement should not be understood as demonstrating the absence of publication bias. Trial registries and dedicated grey-literature databases were not systematically searched. In addition, fewer than 10 studies were available for FM, HDL-C, LDL-C, TC, and TG, which limited the ability to assess publication bias for these outcomes. Therefore, the possibility of publication bias could not be excluded.

The certainty of evidence was rated as low for BW and very low for BMI, BF%, FM, VO_2_max/VO_2_peak, HDL-C, LDL-C, TC, and TG. Accordingly, HIIT may reduce BW compared with controls without structured exercise, whereas the evidence regarding the magnitude of its effects on the remaining outcomes is very uncertain. Detailed outcome-specific downgrade reasons are presented in **Table 1**.

## 4 Discussion

The present review found that the pooled estimates generally favored HIIT for body composition, cardiorespiratory fitness, and selected lipid outcomes when compared with controls without structured exercise. However, the certainty of evidence was low for BW and very low for all remaining outcomes. Across the additional sensitivity analyses, exclusion of studies judged to be at high risk of bias did not alter the direction or statistical interpretation of any of the nine outcomes. Similarly, exclusion of the study involving women with normal-weight obesity did not alter the statistical interpretation of BW, BMI, BF%, or FM. In the broader analysis excluding studies with unclear conventional population eligibility, the directions of most estimates were maintained, but statistical significance was not retained for BW, BMI, or BF%. The leave-one-out analyses also showed that the primary interpretation remained unchanged for most outcomes, although the statistical significance of the TC estimate varied according to the study omitted. These findings provide some reassurance regarding the direction of several outcomes but also indicate that some body-composition conclusions were sensitive to population-eligibility assumptions. Nevertheless, heterogeneity was not uniformly reduced, and the magnitude of several pooled estimates remained sensitive to study exclusion. The sensitivity analyses should therefore be regarded as exploratory assessments of stability rather than confirmation of precise or definitive intervention effects. Given the limited information size, clinical and methodological heterogeneity, and low to very low certainty of evidence, the findings require cautious interpretation.

### 4.1 Effects of HIIT on body composition

The pooled estimates favored HIIT for BW, BMI, BF%, and FM compared with controls without structured exercise. However, the certainty of evidence was low for BW and very low for BMI, BF%, and FM. Accordingly, HIIT may produce a modest reduction in BW, whereas the true magnitude and consistency of its effects on BMI, BF%, and FM remain very uncertain. Although these outcomes are all related to obesity status, they do not capture identical physiological constructs. BW and BMI primarily reflect overall body mass burden and height-adjusted adiposity, whereas BF% and FM more directly reflect the relative proportion and absolute amount of adipose tissue. The findings should therefore be interpreted as pooled directions across related but distinct body-composition constructs rather than as definitive evidence of a uniform weight-loss effect.

In terms of the direction of effect, the effects of HIIT on BW, BMI, BF%, and FM were all oriented toward favorable changes, suggesting that HIIT may have potential intervention value for obesity-related body composition outcomes in women. This finding is broadly consistent with previous evidence indicating that HIIT may improve body composition in individuals with overweight or obesity (Boutcher, 2011; Kong et al., 2016b; Maillard et al., 2016; Keating et al., 2017; Wewege et al., 2017; Batrakoulis et al., 2018; Maillard et al., 2018; Boutcher et al., 2019; Clark et al., 2019; Batitucci et al., 2022; Sanca-Valeriano et al., 2023). Compared with previous studies, the present review further focused on women with overweight or obesity and incorporated both general body mass indicators and adipose tissue-related outcomes, thereby allowing a more comprehensive evaluation of the effects of HIIT on female body composition. In particular, reductions in BF% and FM suggest that the effects of HIIT may extend beyond changes in body weight alone and may involve improvements at the level of adipose tissue.

From a mechanistic perspective, the effects of HIIT on body composition may be partly attributable to its potent metabolic stimulus. HIIT typically consists of repeated high-intensity exercise bouts interspersed with recovery periods, allowing a relatively high exercise load to be achieved within a short period of time. This may increase energy expenditure during exercise and may further augment total energy expenditure through excess post-exercise oxygen consumption (Kessler et al., 2012; Cassidy et al., 2017). In addition, high-intensity interval stimuli may promote skeletal muscle oxidative adaptations, enhance fat oxidation capacity, and improve insulin sensitivity, thereby potentially contributing to reductions in adipose tissue (Talanian et al., 2007; Trapp et al., 2008; Hood et al., 2011; Little et al., 2011; Cocks et al., 2013; Gillen et al., 2013; Jelleyman et al., 2015; dos Santos et al., 2021). These mechanisms provide a biologically plausible explanation for the improvements in BF% and FM observed in the present review. However, it should be emphasized that this meta-analysis did not directly synthesize outcomes related to fat oxidation, energy metabolism, insulin sensitivity, or relevant molecular mechanisms. Therefore, the above explanations should be regarded as biologically plausible inferences based on previous research, rather than causal mechanisms directly established by the present meta-analysis.

The between-study heterogeneity observed for BMI, BF%, and FM appeared to be multifactorial. Univariable meta-regression suggested that baseline BMI and intervention duration may be associated with variation in the BMI effect estimates, training frequency may be associated with variation in the BF% effect estimates, and baseline BMI and training frequency may be associated with variation in the FM effect estimates. However, these models were based on only 8–11 effect sizes, and the corresponding findings were not uniformly supported by the categorical subgroup comparisons. Because the analyses used aggregate study-level characteristics, the observed associations may also be affected by ecological confounding and correlations between participant characteristics and intervention design. These results should therefore be considered exploratory rather than evidence that the identified variables independently or causally modify the effects of HIIT.

The leave-one-out analyses showed that the negative direction and statistical significance of the pooled BMI, BF%, and FM effects remained unchanged after omission of individual studies. Nevertheless, omission of Kazemi et al. (2023) attenuated the BMI estimate from −0.560 to −0.396 and reduced I^2^ from 67.67% to 25.18%. Similarly, omission of Hu et al. (2022) attenuated the BF% estimate from −0.762 to −0.523 and reduced I^2^ from 83.39% to 43.63%; for FM, omission of the same study attenuated the estimate from −0.656 to −0.471 and reduced I^2^ to 0%. These patterns suggest that individual studies contributed to the magnitude of the pooled estimates and to part of the observed heterogeneity, but they do not establish those studies as definitive sources of heterogeneity. Differences in baseline adiposity, health and endocrine status, body-composition measurement methods, HIIT prescriptions, and comparator conditions may have jointly contributed to the variation across studies.

Overall, the pooled directions favored HIIT across the four body-composition outcomes. Nevertheless, the certainty of evidence was low for BW and very low for BMI, BF%, and FM. HIIT may therefore reduce BW, but the true magnitude of its effects on the remaining body-composition outcomes is very uncertain. The observed heterogeneity and sensitivity of several estimates to individual studies also prevent the results from being used to define an optimal HIIT prescription. Larger trials using standardized body-composition measurements and clearly reported training prescriptions are needed to obtain more precise estimates.

### 4.2 Effects of HIIT on cardiorespiratory fitness

The pooled estimate favored HIIT for VO_2_max/VO_2_peak in women with overweight or obesity. However, the certainty of evidence was very low because of risk-of-bias concerns, between-study heterogeneity, and limited precision. The result should therefore be interpreted as suggesting a possible improvement in cardiorespiratory fitness rather than as establishing a clear or precisely quantified effect. VO_2_max/VO_2_peak remains an important integrated indicator of oxygen uptake, circulatory transport, and skeletal muscle oxygen utilization and is closely related to exercise tolerance and broader cardiovascular health. Nevertheless, the clinical magnitude and consistency of the observed pooled effect require confirmation in more rigorous trials.

From a mechanistic perspective, the improvement in VO_2_max/VO_2_peak associated with HIIT may involve both central circulatory adaptations and peripheral skeletal muscle adaptations. At the central level, repeated bouts of high-intensity interval exercise acutely challenge heart rate, myocardial contractility, and circulatory blood flow demand, which may contribute to improvements in stroke volume, cardiac output, and vasodilatory function, thereby enhancing oxygen delivery during exercise (Kessler et al., 2012; Ramos et al., 2015; Cassidy et al., 2017). At the peripheral level, HIIT may promote skeletal muscle oxidative adaptations through repeated high-intensity metabolic stress, including improvements in mitochondrial function, oxidative enzyme activity, capillary perfusion, and muscular oxygen extraction capacity, which may collectively enhance the efficiency of oxygen utilization by skeletal muscle (Talanian et al., 2007; Cocks et al., 2013; Gillen et al., 2013). In addition, HIIT may improve fat oxidation capacity and insulin sensitivity, providing a metabolic basis for improvements in cardiorespiratory fitness (Talanian et al., 2007; Cocks et al., 2013; Batacan et al., 2017). Taken together, these mechanisms suggest that improvements in VO_2_max/VO_2_peak are unlikely to arise from a single physiological pathway, but may instead reflect coordinated adaptations in cardiovascular oxygen transport and skeletal muscle oxygen utilization.

The primary studies included in this review also provide some support for these mechanistic interpretations. Previous studies in female populations have shown that short-term HIIT may increase VO_2_max in women with overweight or obesity, and such improvements may be related to enhanced skeletal muscle mitochondrial oxidative enzyme activity, greater muscular oxygen extraction capacity, and increased stroke volume (Cocks et al., 2013; Gillen et al., 2013; Cassidy et al., 2017). In women with PCOS, improvements in aerobic capacity after HIIT have also been suggested to be associated with enhanced cardiac output, oxygen transport capacity, and peripheral oxygen utilization efficiency (Batacan et al., 2017). These findings are consistent with the direction of the pooled estimates in the present review, suggesting that HIIT may have potential value for improving cardiorespiratory fitness in women with overweight or obesity across different health-status subgroups. Overall, the effects of HIIT on VO_2_max/VO_2_peak may reflect the combined contribution of central circulatory adaptations and peripheral skeletal muscle metabolic adaptations.

Although the pooled VO_2_max/VO_2_peak estimate favored HIIT, moderate between-study heterogeneity remained. Univariable meta-regression suggested that training frequency may be associated with variation in the effect estimates; however, the subgroup with a training frequency of >3 sessions/week contained only one effect size, making this association particularly vulnerable to sparse-data and individual-study effects. The leave-one-out analyses provided some reassurance regarding the overall interpretation: all recalculated effect estimates remained positive and statistically significant, ranging from 0.981 to 1.172. Omission of Zhang et al. (2015) produced the largest reduction in heterogeneity, with I^2^ decreasing from 66.48% to 55.86% and τ^2^ from 0.2977 to 0.1811, but moderate residual heterogeneity remained. Thus, the favorable direction of the VO_2_max/VO_2_peak result was not dependent on a single study, whereas the variation in effect magnitude may reflect combined differences in training frequency, exercise modality, intervention dose, outcome-testing procedures, and participant health status. The training-frequency finding should therefore remain exploratory and should not be used to define an optimal HIIT frequency.

Overall, the pooled estimate suggests that HIIT may improve cardiorespiratory fitness in women with overweight or obesity. However, because the certainty of evidence was very low, the true magnitude and consistency of this effect remain very uncertain. The proposed central circulatory, skeletal muscle, and mitochondrial mechanisms are biologically plausible but were not directly evaluated in the present meta-analysis and should not be interpreted as causal pathways established by the pooled evidence.

Future studies should further clarify the effects of different HIIT doses on VO_2_max/VO_2_peak in women with overweight or obesity using standardized testing methods and more detailed reporting of training prescriptions. In addition, integrating cardiopulmonary exercise testing, hemodynamic measures, and skeletal muscle metabolic indicators may help more precisely elucidate the physiological mechanisms through which HIIT improves cardiorespiratory fitness.

### 4.3 Effects of HIIT on lipid profiles

The pooled estimates favored HIIT for an increase in HDL-C and a reduction in LDL-C, whereas the confidence intervals for TC and TG crossed the line of no effect. However, the certainty of evidence was very low for all four lipid outcomes. The evidence is therefore very uncertain regarding the true magnitude of the effects on HDL-C and LDL-C and does not establish clear effects on TC or TG. These findings should not be interpreted as demonstrating that HIIT comprehensively improves the lipid profile.

From a mechanistic perspective, the effects of HIIT on lipid profiles may be related to enhanced fatty acid oxidation, skeletal muscle metabolic adaptations, and improvements in insulin sensitivity. By alternating high-intensity exercise bouts with recovery periods, HIIT can impose a substantial metabolic load within a relatively short period, increase energy expenditure during and after exercise, and promote fatty acid mobilization and oxidative utilization (Kessler et al., 2012; Cassidy et al., 2017). Repeated high-intensity stimuli may also enhance skeletal muscle mitochondrial function, oxidative enzyme activity, and the capacity to utilize lipid substrates, thereby improving the clearance and utilization of fatty acids in peripheral tissues (Talanian et al., 2007; Cocks et al., 2013; Gillen et al., 2013). These skeletal muscle oxidative adaptations may not only contribute to reduced fat accumulation but may also influence LDL-C and TG through improvements in insulin sensitivity and hepatic lipoprotein metabolism (Cocks et al., 2013; Batacan et al., 2017). Therefore, the effects of HIIT on lipid profiles may not depend solely on body weight reduction, but may instead be related to the combined effects of reduced adipose tissue, enhanced skeletal muscle metabolic capacity, and improved systemic metabolic status.

The favorable directions observed for HDL-C and LDL-C may indicate that these lipoprotein-related markers respond to exercise-induced metabolic adaptations; however, given the very low certainty of evidence, they should not be described as established improvements. Exercise training may exert favorable effects on HDL-C and LDL-C by improving lipoprotein-metabolizing enzyme activity, facilitating cholesterol transport, and enhancing peripheral lipid utilization (Racil et al., 2013; Alvarez et al., 2018; Cicek et al., 2024). Previous studies have also suggested that HIIT may improve cardiometabolic health in individuals with overweight or obesity, with potential mechanisms including enhanced fat oxidation capacity, improved insulin sensitivity, and reduced inflammatory status (Talanian et al., 2007; Cocks et al., 2013; Kong et al., 2016a; Batacan et al., 2017; Camacho-Cardenosa et al., 2018; Reljic et al., 2020; Serrablo-Torrejon et al., 2020; Ahmad et al., 2023). These lines of evidence are consistent with the observed direction of increased HDL-C and reduced LDL-C in the present review, suggesting that HIIT may have favorable effects on lipoprotein metabolism in women with overweight or obesity.

In contrast, the responses of TC and TG to HIIT may be less consistent. TC reflects the combined contribution of multiple cholesterol fractions, and changes in TC may be offset by divergent changes in HDL-C and LDL-C. TG, in turn, is highly dependent on recent dietary intake, energy balance, hepatic lipid synthesis, and the degree of insulin resistance. In women with overweight or obesity, factors such as PCOS, menopausal status, a history of breast cancer treatment, or coexisting metabolic abnormalities may further influence baseline lipid metabolism. If dietary intake, medication use, and baseline lipid levels were not adequately controlled in the original studies, true changes in TC and TG may be more difficult to detect consistently during short-term exercise interventions. Therefore, the non-significant effects on TC and TG should not be interpreted simply as evidence that HIIT has no effect on these outcomes; rather, they indicate that the current evidence remains insufficient to support definitive conclusions.

The subgroup and meta-regression findings suggest that participant age may be one potential study-level factor associated with variability in lipid responses. Statistically significant study-level associations with age were observed for HDL-C, TC, and TG, while the association for LDL-C was borderline. Nevertheless, age may be correlated across studies with menopausal status, comorbid metabolic conditions, baseline lipid concentrations, medication use, and other unmeasured characteristics. The apparent age-related pattern may therefore reflect a combination of these factors rather than an independent age effect. Given the limited number of effect sizes available for each lipid outcome, these associations should be regarded as hypothesis-generating and not as evidence of an individual-level or causal effect of age.

The leave-one-out analyses showed that the stability of the statistical interpretation differed across lipid outcomes. For HDL-C and LDL-C, the direction and statistical interpretation remained unchanged after omission of individual studies, although the effect magnitudes varied and residual heterogeneity remained. For TG, all recalculated confidence intervals continued to cross the line of no effect, indicating that the uncertain and non-significant interpretation was not attributable to omission of a single study. For TC, statistical significance varied according to the study omitted, indicating particular sensitivity to individual studies. These findings should not be interpreted as demonstrating robust lipid effects; rather, they show that uncertainty remained in the magnitude, consistency, and precision of the pooled estimates.

Overall, the pooled directions favored an increase in HDL-C and a reduction in LDL-C, but the certainty of evidence was very low and the true effects remain very uncertain. No clear effect was established for TC or TG, and these estimates were also supported by very-low-certainty evidence. The lipid findings should therefore be regarded as preliminary and should not be used independently to support clinical recommendations.

Future studies should further clarify the effects of HIIT on lipid metabolism in women with overweight or obesity under more rigorous control of dietary intake, medication use, menstrual or menopausal status, and baseline lipid levels. In addition, incorporating measures related to fat oxidation capacity, insulin sensitivity, inflammatory responses, and lipoprotein metabolism may help more accurately elucidate the specific pathways through which HIIT may influence lipid profiles in this population.

### 4.4 Limitations of the present review

The present review has several limitations. First, the number of studies and participants available for several outcomes was limited, particularly for lipid-related outcomes and some subgroup and meta-regression analyses. The relatively small evidence base reduced the precision of the pooled estimates and limited the statistical power available to distinguish genuine effect modification from chance variation.

Second, considerable clinical and methodological diversity remained across the included studies. The HIIT protocols differed in exercise modality, training intensity, interval structure, training frequency, session duration, intervention duration, progression, and supervision. The study populations also varied in age, baseline adiposity, health status, endocrine status, and metabolic-risk profile, while comparator conditions and outcome-measurement methods were not uniform. Although random-effects models were used, these differences may have contributed to the observed heterogeneity and limit the extent to which a single pooled estimate can represent all populations and intervention settings. The revised RoB 2 assessment identified some concerns in 11 trials and high overall risk in four trials, primarily because of limitations in the randomization process, missing outcome data, and the availability of prospective protocols or prespecified analysis plans. Although potential differences across outcome types were considered when judging missing data, outcome measurement, and selection of the reported result, the use of one conservative summary row per study may not capture every difference between individual outcomes. The risk-of-bias findings should therefore be interpreted together with the detailed supporting judgements in **Supplementary Table 5**. Furthermore, the exclusive use of Hedges’ g provided a consistent standardized metric across the outcome-specific meta-analyses and reduced small-sample bias. However, standardized effects are less directly interpretable in familiar clinical units; for example, the pooled BW and BMI effects cannot be interpreted directly as absolute changes in kilograms or kg/m^2^. The findings should therefore be understood primarily in terms of the direction and standardized magnitude of the between-group effects.

Third, although the moderator domains and coding categories were prespecified before statistical analyses, all subgroup and meta-regression analyses remained exploratory and hypothesis-generating. Prespecification reduced the risk of purely data-driven moderator selection but did not overcome the limitations arising from the small number of studies, sparse or unbalanced moderator categories, aggregate study-level data, and multiple comparisons. The meta-regression models included only 8–12 independent effect estimates, and some subgroup categories contained only one or two studies. Consequently, the regression coefficients, residual heterogeneity estimates, and estimated R^2^ values may be unstable and sensitive to individual studies. In particular, an estimated R^2^ of 100% should not be interpreted as demonstrating that a moderator completely explains the observed heterogeneity. Study-level associations may also be affected by ecological confounding and correlations between covariates and should not be interpreted as individual-level or causal relationships. The large number of exploratory comparisons increases the possibility that some statistically significant findings arose by chance. Accordingly, these analyses should be used to generate hypotheses for future adequately powered trials and should not be used directly to define an optimal HIIT prescription.

Fourth, the additional sensitivity analyses were conducted *post hoc* and were frequently based on a limited number of studies. Exclusion of studies judged to be at overall high risk of bias did not alter the statistical interpretation of the nine outcomes, but it reduced the available evidence base, and the retained studies were not necessarily free from methodological concerns. In addition, the overall RoB 2 classifications represented conservative study-level summaries and may not fully capture outcome-specific differences in risk of bias. The population-definition analysis excluding Hu et al. (2022) examined the findings under a narrower conventional BMI-based population framework and should not be interpreted as demonstrating that the original study was ineligible. A separate sensitivity analysis excluding studies with unclear conventional population eligibility showed that statistical significance was not retained for BW, BMI, or BF%. HDL-C and LDL-C could not be quantitatively pooled after exclusion, whereas the TC and TG analyses were based on only two studies. Accordingly, the applicability of the pooled findings to populations defined strictly according to conventional BMI-based overweight or obesity criteria remains uncertain. The leave-one-out analyses identified studies associated with comparatively large changes in effect magnitude or heterogeneity, but they cannot determine causal sources of heterogeneity. I^2^ and τ^2^ estimates may also be unstable when calculated from small numbers of studies. Furthermore, change-score sensitivity analyses could not be performed because change scores and their dispersion measures were not reported sufficiently consistently. Collectively, the sensitivity analyses provide information regarding the relative stability of the primary findings but should not be regarded as confirmation that the estimated effects are precise, unbiased, or fully robust.

Fifth, the assessment of publication bias was limited by the small number of studies available for several outcomes. Egger’s tests for FM, HDL-C, LDL-C, TC, and TG were based on fewer than 10 independent effect estimates and therefore had limited power to detect funnel-plot asymmetry. Although no outcome was downgraded for publication bias in the GRADE assessment, this decision reflected the absence of direct evidence strongly suggesting publication bias rather than evidence that publication bias was absent. In addition, the certainty of evidence was low for BW and very low for all remaining outcomes, which limits confidence in the estimated magnitudes and should be considered when interpreting statistically significant pooled effects. Furthermore, trial registries and dedicated grey-literature databases were not systematically searched, which may have increased the possibility of missing ongoing, unpublished, or difficult-to-locate trials. In addition, the Cochrane Library, including the Cochrane Central Register of Controlled Trials, and Scopus were not systematically searched. Although PubMed, MEDLINE via Ovid, Embase, Web of Science Core Collection, and supplementary citation searching provided broad and complementary coverage, the omission of these additional sources may have resulted in eligible trials being missed. Accordingly, the search should be regarded as broad but not exhaustive.

Sixth, the present review did not assess the full spectrum of cardiometabolic health. The quantitative synthesis was limited to body composition, cardiorespiratory fitness, and selected lipid outcomes. Blood pressure, insulin resistance, glycemic control, and inflammatory markers were not systematically synthesized; therefore, the findings should not be generalized to overall cardiometabolic health.

Seventh, reporting of intervention implementation and safety was incomplete and inconsistent. Adherence and attendance were defined differently across trials, dropout reasons were not always fully reported, adverse-event reporting was limited, and actual completed exercise dose was rarely described in sufficient detail. The degree and setting of exercise supervision also varied. Consequently, the present review cannot determine the acceptability, safety, or real-world feasibility of a specific HIIT protocol, and practical recommendations should remain cautious.

Future randomized controlled trials should use larger sample sizes, more clearly defined participant groups, standardized outcome-assessment methods, and more transparent reporting of HIIT prescriptions. Greater control and reporting of dietary intake, medication use, habitual physical activity, menstrual or menopausal status, baseline metabolic profile, intervention adherence, and long-term follow-up are also needed to improve the precision, comparability, and clinical interpretability of the evidence.

### 4.5 Practical implications and future directions

The available evidence suggests that HIIT may be considered as one possible exercise option for adult women with overweight or obesity, particularly in relation to adiposity-related body-composition outcomes and cardiorespiratory fitness. However, the present review assessed only body composition, cardiorespiratory fitness, and selected lipid outcomes and should not be interpreted as demonstrating comprehensive cardiometabolic benefit. The certainty of evidence was low for BW and very low for all other outcomes; therefore, the pooled findings do not provide sufficiently certain evidence to support established benefits across all assessed outcomes.

Recommendations for routine clinical implementation remain premature because adherence, attrition, adverse-event, supervision, and actual completed-dose data were incompletely and inconsistently reported across the included trials, as summarized in **Supplementary Table 6**. An intervention that is effective under closely supervised trial conditions may not necessarily be equally acceptable, sustainable, feasible, or safe in routine practice. HIIT should therefore not be implemented as a fixed or universally applicable program. Where HIIT is considered, program design should be individualized according to baseline fitness, health status, comorbidities, exercise experience, preferences, and tolerance; introduced progressively; and accompanied by appropriate pre-exercise screening, professional supervision where required, intensity monitoring, and procedures for recording adherence and adverse events.

Although some subgroup and meta-regression analyses suggested possible differences according to participant or intervention characteristics, these analyses were exploratory and hypothesis-generating. They should not be translated into definitive age-, BMI-, duration-, frequency-, health-status-, or modality-specific HIIT prescriptions. The available data also do not establish a specific optimal training dose or a dose-response threshold. Current program design should therefore continue to be guided by established exercise principles, participant safety, feasibility, adherence, and individual preferences rather than by isolated moderator findings from the present review.

Future trials should separately report prescribed and completed exercise dose and provide standardized information on session attendance, adherence definitions, reasons for dropout, adverse events, supervision personnel and setting, dose modification, and progression. Larger and adequately powered randomized trials should also use standardized outcome measurements, transparent prespecified analyses, and longer follow-up to determine the clinical relevance, sustainability, and appropriate implementation of HIIT in this population (Garber et al., 2011).

## 5 Conclusion

The available evidence suggests that, compared with controls without structured exercise, HIIT may offer potential benefits for adiposity-related body-composition outcomes and cardiorespiratory fitness in adult women with overweight or obesity, whereas its effects on selected lipid outcomes remain uncertain. These findings should not be interpreted as a comprehensive evaluation of cardiometabolic health because blood pressure, insulin resistance, glycemic control, and inflammatory markers were not systematically synthesized. Confidence in the findings is limited by the low to very low certainty of evidence, small study samples, and substantial clinical and methodological heterogeneity.

HIIT may therefore be considered as one possible component of an individualized exercise program, but the current evidence does not establish its superiority over other active exercise modalities, a universally effective protocol, or an optimal training dose. Incomplete and inconsistent reporting of adherence, attrition, adverse events, supervision, and actual completed exercise dose also prevents firm conclusions regarding feasibility, safety, and routine clinical implementation. Larger and adequately powered randomized controlled trials with improved allocation procedures, more complete outcome data, standardized measurements, transparent prespecification, and standardized reporting of both efficacy and implementation outcomes are required before definitive clinical or public-health recommendations can be made.

## Data Availability

The original contributions presented in the study are included in the article/**Supplementary Material**. Further inquiries can be directed to the corresponding author.
