# Supplementary material for: Effects of high-intensity interval training on body composition, cardiorespiratory fitness, and selected lipid outcomes in adult women with overweight or obesity: a systematic review and meta-analysis
Source: Front Physiol. 2026 Sep 22;17:1904753. doi: 10.3389/fphys.2026.1904753 (PMC13639141; doi:10.3389/fphys.2026.1904753)
Supplement: Supplementary file 1 [file DataSheet1.pdf]

Database	Search strategy	Records retrieved (n)
PubMed	("High-Intensity Interval Training"[Mesh] OR "high-intensity interval training"[Title/Abstract] OR "high intensity interval training"[Title/Abstract] OR HIIT[Title/Abstract] OR "high-intensity interval exercise"[Title/Abstract] OR "high intensity interval exercise"[Title/Abstract] OR "high-intensity intermittent exercise"[Title/Abstract] OR "high intensity intermittent exercise"[Title/Abstract] OR "high-intensity intermittent training"[Title/Abstract] OR "high intensity intermittent training"[Title/Abstract] OR "sprint interval training"[Title/Abstract] OR "interval training"[Title/Abstract]) AND ("Obesity"[Mesh] OR "Overweight"[Mesh] OR obes*[Title/Abstract] OR overweight[Title/Abstract] OR adiposity[Title/Abstract] OR "normal-weight obesity"[Title/Abstract] OR "normal weight obesity"[Title/Abstract]) AND (randomized controlled trial[Publication Type] OR controlled clinical trial[Publication Type] OR random*[Title/Abstract] OR trial[Title/Abstract] OR placebo[Title/Abstract]) NOT (animals[Mesh] NOT humans[Mesh])	685
Web of Science Core Collection	TS=(("high-intensity interval training" OR "high intensity interval training" OR "high-intensity interval exercise" OR "high intensity interval exercise" OR "high-intensity intermittent training" OR "high intensity intermittent training" OR "high-intensity intermittent exercise" OR "high intensity intermittent exercise" OR "sprint interval training" OR "sprint interval exercise" OR "aerobic interval training" OR "interval aerobic training" OR "repeated sprint training" OR "high-intensity functional training" OR "high intensity functional training" OR "interval training" OR HIIT OR HIIE OR HIFT OR Tabata) AND (overweight OR obes* OR adipos* OR "excess weight" OR "weight excess" OR "normal-weight obesity" OR "normal weight obesity" OR BMI OR "body mass index") AND (random* OR trial* OR allocat* OR assign* OR control* OR crossover OR "cross-over" OR placebo* OR "intervention study" OR "intervention studies"))	1,585
Embase	('high intensity interval training'/exp OR 'high intensity interval training':ti,ab,kw OR 'high-intensity interval training':ti,ab,kw OR hiit:ti,ab,kw OR hiie:ti,ab,kw OR 'high intensity interval exercise':ti,ab,kw OR 'high-intensity interval exercise':ti,ab,kw OR 'high intensity intermittent exercise':ti,ab,kw OR 'high-intensity intermittent exercise':ti,ab,kw OR 'high intensity intermittent training':ti,ab,kw OR 'high-intensity intermittent training':ti,ab,kw OR 'sprint interval training':ti,ab,kw OR 'sprint interval exercise':ti,ab,kw OR 'aerobic interval training':ti,ab,kw OR 'interval training':ti,ab,kw) AND ('obesity'/exp OR 'overweight'/exp OR obes*:ti,ab,kw OR overweight:ti,ab,kw OR adipos*:ti,ab,kw OR 'excess weight':ti,ab,kw OR 'excess body weight':ti,ab,kw OR 'normal-weight obesity':ti,ab,kw OR 'normal weight obesity':ti,ab,kw) AND ('female'/exp OR woman:ti,ab,kw OR women:ti,ab,kw OR female*:ti,ab,kw) AND ('randomized controlled trial'/exp OR 'controlled clinical trial'/exp OR random*:ti,ab,kw OR allocat*:ti,ab,kw OR assign*:ti,ab,kw OR trial*:ti,ab,kw OR placebo*:ti,ab,kw OR crossover:ti,ab,kw OR 'cross	1,081

Database	Search strategy	Records retrieved (n)
	over':ti,ab,kw OR control*:ti,ab) NOT ('animal'/exp NOT 'human'/exp)	
MEDLINE via Ovid	1. exp High-Intensity Interval Training/ or high-intensity interval training.ti,ab,kf. or high intensity interval training.ti,ab,kf. or HIIT.ti,ab,kf. or high-intensity interval exercise.ti,ab,kf. or high intensity interval exercise.ti,ab,kf. or high-intensity intermittent exercise.ti,ab,kf. or high intensity intermittent exercise.ti,ab,kf. or high-intensity intermittent training.ti,ab,kf. or high intensity intermittent training.ti,ab,kf. or HIIE.ti,ab,kf. or sprint interval training.ti,ab,kf. or sprint interval exercise.ti,ab,kf. or aerobic interval training.ti,ab,kf. or repeated sprint training.ti,ab,kf. or interval training.ti,ab,kf. or Tabata.ti,ab,kf. 2. exp Overweight/ or exp Obesity/ or overweight.ti,ab,kf. or obes*.ti,ab,kf. or adipos*.ti,ab,kf. or excess weight.ti,ab,kf. or excess body weight.ti,ab,kf. or normal-weight obesity.ti,ab,kf. 3. exp Women/ or Female/ or wom#n.ti,ab,kf. or female*.ti,ab,kf. 4. exp randomized controlled trial/ or controlled clinical trial.pt. or randomized.ab. or randomised.ab. or placebo.ab. or randomly.ab. or trial.ab. or groups.ab. 5. 1 and 2 and 3 and 4 6. 5 not (exp animals/ not humans.sh.)	227

Supplementary Table S1. Search strategies and numbers of records retrieved.

Note: All database searches covered the period from database inception to 26 July 2026.

Study	Original study arm	Sample size, n	Intervention classification or co-intervention	Eligibility decision	Comparison retained in the meta-analysis	Rationale
Smith-Ryan et al., 2016	1MIN	11	Classified as SIT in the original study	Excluded from the primary analysis*	Not applicable	The original study distinguished the 1MIN protocol as SIT. This arm was outside the operational intervention definition adopted for the primary analysis.*
Smith-Ryan et al., 2016	2MIN	10	Classified as HIT in the original study; five 2-min intervals at 80%–100% VO_2peak	Included	2MIN versus non-exercise control	The protocol was explicitly classified as HIT and was consistent with the aerobic HIIT intervention definition used in the primary analysis.
Smith-Ryan et al., 2016	Non-exercise control	9	No structured exercise intervention	Included	Comparator for the 2MIN group	The group met the prespecified non-HIIT comparator criteria.

Study	Original study arm	Sample size, n	Intervention classification or co-intervention	Eligibility decision	Comparison retained in the meta-analysis	Rationale
Sanayei et al., 2022	HIIT + *Chlorella vulgaris*	12	HIIT combined with an active nutritional supplement	Excluded	Not applicable	The additional active supplementation was not matched in the selected comparator, and the independent effect of HIIT could not be isolated.
Sanayei et al., 2022	HIIT + placebo	11	HIIT combined with placebo supplementation	Included	HIIT + placebo versus placebo	Both groups received placebo supplementation; therefore, the principal between-group difference was the HIIT intervention.
Sanayei et al., 2022	*Chlorella vulgaris* only	12	Active nutritional supplementation without HIIT	Excluded	Not applicable	The group received an active non-exercise intervention and was not the matched comparator for estimating the independent effect of HIIT.
Sanayei et al., 2022	Placebo only	11	Placebo supplementation without HIIT	Included	Comparator for the HIIT + placebo group	The group provided the matched non-HIIT comparator required to isolate the effect of HIIT.

Supplementary Table S2. Arm-level eligibility decisions for included multi-arm trials

Note: HIIT, high-intensity interval training; HIT, high-intensity training; SIT, sprint interval training; VO_2peak, peak oxygen uptake. Each arm of a multi-arm trial was evaluated separately against the prespecified eligibility criteria. Arm-level decisions were based on intervention eligibility and the ability to isolate the independent effect of HIIT, rather than on the magnitude or statistical significance of the reported findings

The 1MIN arm was excluded because it was explicitly classified as sprint interval training in the original study and did not meet the operational definition of aerobic HIIT adopted for the primary analysis.

Outcome	Primary Hedges' g （95% CI; P）	Primary I²; τ²	Leave-one-out g range	Leave-one-out P range	Leave-one-out I² range; τ² range	Potentially influential study or studies	Robustness assessment
BMI	−0.560（−0.945 to −0.174; P=0.005）	67.67%; 0.2810	−0.633 to −0.396	0.001 to 0.015	25.18% to 71.80%; 0.0435 to 0.3382	Omission of Kazemi et al. 2023 changed g to −0.396 and reduced I² to 25.18% and τ² to 0.0435	Direction and statistical significance were stable, but the magnitude and heterogeneity estimates were sensitive to Kazemi et al. 2023
BF%	−0.762（−1.354 to −0.169; P=0.012）	83.39%; 0.7454	−0.884 to −0.523	0.002 to 0.034	43.63% to 85.85%; 0.1080 to 0.8990	Omission of Hu et al. 2022 changed g to −0.523 and reduced I² to 43.63% and τ² to 0.1080	Direction and statistical significance were stable, but the magnitude and heterogeneity estimates were sensitive to Hu et al. 2022

FM	−0.656（−1.108 to −0.204; P=0.004）	64.04%; 0.2655	−0.764 to −0.471	0.001 to 0.018	0.00% to 70.60%; 0.0000 to 0.3727	Omission of Hu et al. 2022 changed g to −0.471 and reduced both I^2 and τ^2 to 0	Direction and statistical significance were stable, although Hu et al. 2022 had a marked influence on the magnitude and heterogeneity estimates
VO_2max/VO_2peak	1.085（0.700 to 1.470; P<0.001）	66.48%; 0.2977	0.981 to 1.172	All P<0.001	55.86% to 70.33%; 0.1811 to 0.3606	Omission of Zhang et al. 2015 changed g to 0.981 and reduced I^2 to 55.86% and τ^2 to 0.1811	Direction and statistical significance were stable; no individual study altered the overall interpretation, although moderate heterogeneity remained
HDL-C	0.843（0.240 to 1.446; P=0.006）	80.90%; 0.5998	0.605 to 1.000	0.001 to 0.027	65.77% to 84.34%; 0.2565 to 0.7342	Omission of Kazemi et al. 2023 changed g to 0.605 and reduced I^2 to 65.77% and τ^2 to 0.2565	Direction and statistical significance were stable, but effect magnitude varied and substantial residual heterogeneity remained

LDL-C	−0.780（−1.293 to −0.266; P=0.003）	74.16%; 0.4011	−0.904 to −0.575	0.001 to 0.015	50.75% to 77.45%; 0.1494 to 0.4844	Omission of Taha et al. 2023 changed g to −0.575 and reduced I^2 to 50.75% and τ^2 to 0.1494	Direction and statistical significance were stable, but the effect magnitude and heterogeneity estimates were sensitive to Taha et al. 2023
TC	−0.346（−0.701 to 0.009; P=0.056）	54.23%; 0.1569	−0.432 to −0.246	0.015 to 0.161	41.53% to 60.06%; 0.1014 to 0.1987	Omission of Taha et al. 2023 changed g to −0.246 and I^2 to 41.53%; omission of Kazemi et al. 2023 changed g to −0.252 and I^2 to 43.86%	The direction remained negative, but statistical significance varied across analyses, indicating limited robustness
TG	−0.240（−0.689 to 0.209; P=0.295）	71.09%; 0.3294	−0.344 to −0.099	0.128 to 0.628	56.45% to 74.85%; 0.1855 to 0.3940	Omission of Taha et al. 2023 changed g to −0.099 and reduced I^2 to 56.45%; omission of Kazemi et al. 2023 changed g to −0.105 and I^2 to 59.68%	The negative direction and non-significant interpretation were stable, although moderate-to-high heterogeneity remained

Supplementary Table S3. Summary of leave-one-out sensitivity analyses for outcomes with I^2 ≥50% in the primary analysis

Table note: The primary estimate was calculated using all eligible studies. Leave-one-out ranges represent the minimum and maximum values obtained after sequential omission of individual studies. Negative Hedges' g values indicate effects favouring HIIT for BMI, BF%, FM, LDL-C, TC, and TG, whereas positive values favour HIIT for VO_2max/VO_2peak and HDL-C. Potentially influential studies were identified descriptively according to comparatively large changes in the pooled estimate or heterogeneity statistics; these findings do not establish a definitive source of heterogeneity, and no study was excluded from the primary analysis on this basis. BF%, body fat percentage; FM, fat mass; VO_2max/VO_2peak, maximal or peak oxygen uptake; HDL-C, high-density lipoprotein cholesterol; LDL-C, low-density lipoprotein cholesterol; TC, total cholesterol; TG, triglycerides; CI, confidence interval.

Outcome	K (N)	Primary I^2 (%)	Prespecified subgroup moderators	Meta-regression moderators	Important data-distribution limitations	Interpretation
BW	11 (336)	24.23	Age stage; baseline BMI; intervention duration; training frequency; health status; exercise modality	Mean age; baseline BMI; intervention duration; training frequency	Relatively low heterogeneity; some subgroup categories contained few effect estimates	Exploratory and hypothesis-generating
BMI	11 (343)	67.67	Age stage; baseline BMI; intervention duration; training frequency; health status; exercise modality	Mean age; baseline BMI; intervention duration; training frequency	Limited number of effect estimates and uneven distribution in some categories	Exploratory and hypothesis-generating
BF%	10 (297)	83.39	Age stage; baseline BMI; intervention duration; training frequency; health status; exercise modality	Mean age; baseline BMI; intervention duration; training frequency	The >3 sessions/week and circuit-/functional-training categories contained few effect estimates	Exploratory and hypothesis-generating
FM	8 (229)	64.04	Age stage; baseline BMI; intervention duration; training frequency; health status; exercise modality	Mean age; baseline BMI; intervention duration; training frequency	Fewer than 10 effect estimates; several moderator categories were sparsely populated	Highly exploratory and hypothesis-generating
VO_2max/VO_2peak	12	66.48	Age stage; baseline BMI;	Mean age; baseline BMI;	The >3 sessions/week subgroup contained only one effect	Exploratory and

Outcome	K (N)	Primary I² (%)	Prespecified subgroup moderators	Meta-regression moderators	Important data-distribution limitations	Interpretation
	(370)		intervention duration; training frequency; health status; exercise modality	intervention duration; training frequency	estimate	hypothesis-generating
HDL-C	8 (253)	80.90	Age stage; baseline BMI; intervention duration; training frequency; health status; exercise modality	Mean age; baseline BMI; intervention duration	Fewer than 10 effect estimates; training frequency lacked sufficient between-study variation; some modality categories contained one effect estimate	Highly exploratory and hypothesis-generating
LDL-C	8 (253)	74.16	Age stage; baseline BMI; intervention duration; training frequency; health status; exercise modality	Mean age; baseline BMI; intervention duration	Fewer than 10 effect estimates; training frequency lacked sufficient between-study variation; some modality categories contained one effect estimate	Highly exploratory and hypothesis-generating
TC	9 (276)	54.23	Age stage; baseline BMI; intervention duration; training frequency; health status; exercise modality	Mean age; baseline BMI; intervention duration; training frequency	Fewer than 10 effect estimates; the >3 sessions/week and circuit-/functional-training categories contained one effect estimate	Highly exploratory and hypothesis-generating
TG	9 (276)	71.09	Age stage; baseline BMI; intervention duration; training frequency; health status; exercise modality	Mean age; baseline BMI; intervention duration; training frequency	Fewer than 10 effect estimates; the >3 sessions/week and circuit-/functional-training categories contained one effect estimate	Highly exploratory and hypothesis-generating

Supplementary Table S4. Prespecified moderator definitions, data availability, and interpretation of subgroup and meta-regression analyses

Note: K (N) denotes the number of independent effect estimates and the total number of participants contributing to the corresponding outcome. Because only one eligible comparison per study was retained for each outcome, K also represents the number of contributing studies. The moderator domains and coding categories were defined before statistical analyses. All meta-regression models were univariable random-effects models. “Highly exploratory” indicates that fewer than 10 independent effect estimates were available and/or that the moderator distribution was sparse or unbalanced. These analyses were not interpreted as confirmatory evidence of causal effect modification and were not used to define an optimal HIIT prescription. BW, body weight; BMI, body mass index; BF%, body fat percentage; FM, fat mass; VO_2max, maximal oxygen uptake; VO_2peak, peak oxygen uptake; HDL-C, high-density lipoprotein cholesterol; LDL-C, low-density lipoprotein cholesterol; TC, total cholesterol; TG, triglycerides; HIIT, high-intensity interval training; PCOS, polycystic ovary syndrome.

**Study**	**Outcomes contributed**	**D1**	**D2**	**D3**	**D4**	**D5**	**Overall**
Zhang et al., 2015	BW, BMI, BF%, FM, VO_2max, TC, TG	Some concerns	Low	Some concerns	Some concerns	Some concerns	Some concerns
Smith-Ryan et al., 2016	BF%, FM, VO_2peak, HDL-C, LDL-C, TC, TG	Some concerns	Low	Some concerns	Some concerns	Some concerns	Some concerns
Sanayei et al., 2022	BW, BF%, VO_2max	Low	Low	Some concerns	Some concerns	Low	Some concerns

Hu et al., 2022	BW, BMI, BF%, FM	Some concerns	Low	High	Low	Some concerns	High
Mohammadi et al., 2023	BW, BMI, BF%, VO_2max, HDL-C, LDL-C, TC, TG	Low	Low	Low	Some concerns	Low	Some concerns
Philbois et al., 2022	BW, BMI, VO_2peak, HDL-C, LDL-C, TC, TG	Some concerns	Some concerns	High	Some concerns	Low	High
Benham et al., 2021	BW, BMI, VO_2max, HDL-C, LDL-C, TC, TG	Low	Low	Some concerns	Low	Low	Some concerns
Almenning et al., 2015	BW, BMI, BF%, FM, VO_2max, HDL-C, LDL-C, TC, TG	Low	Low	Some concerns	Some concerns	Low	Some concerns
Guo et al., 2023	BF%, VO_2max	Some concerns	Low	Low	Some concerns	Some concerns	Some concerns

Kazemi et al., 2023	BW, BMI, FM, VO_2peak, HDL-C, LDL-C，TC,TG	Some concerns	Low	Low	Some concerns	Some concerns	Some concerns
Taha et al., 2023	HDL-C, LDL-C, TC, TG	Some concerns	Low	Some concerns	Low	Some concerns	Some concerns
Samhan et al., 2021	BW, BMI, BF%, FM, VO_2peak	Low	Low	Low	Some concerns	Some concerns	Some concerns
Connolly et al., 2017	BW, BMI, BF%, FM, VO_2peak, HDL-C, LDL-C, TG	Some concerns	Low	Low	Some concerns	Some concerns	Some concerns
Arad et al., 2015	BW, BMI, BF%, FM, VO_2peak	Some concerns	Low	High	Some concerns	Some concerns	High
Ballesta-García et al., 2020	BMI	Low	Low	High	Some concerns	Low	High

Supplementary Table S5，Panel A

Study	Key supporting judgement
Zhang et al., 2015	Randomization and allocation-concealment procedures were insufficiently described. Attrition was reported but the analyses were mainly based on available participants, and blinding of assessors for all contributed outcomes was not clearly established. No prospectively available protocol or sufficiently detailed analysis plan was identified.
Smith-Ryan et al., 2016	Block randomization was reported, but allocation concealment was insufficiently described. Attrition was uneven between the selected intervention and control groups, and blinding of outcome assessors was not clearly reported for all outcomes. Trial registration was reported, but the prospective analytical plan could not be fully verified.
Sanayei et al., 2022	Computer-generated randomization and placebo matching supported low risk in the randomization and intervention-deviation domains. However, attrition differed between groups and was addressed using last-observation-carried-forward analysis. Some cardiorespiratory outcomes were susceptible to participant effort or test procedures.
Hu et al., 2022	Random sequence generation and allocation concealment were insufficiently described. Ten of 40 randomized participants did not complete the study, with greater loss in the control group than in the HIIT group. The substantial and unevenly distributed missing data resulted in a high-risk judgement.
Mohammadi et al., 2023	Computer-generated randomization and sequentially numbered, opaque, sealed envelopes were reported. Missing data were limited and balanced between groups. Some concerns remained because the field-based cardiorespiratory-fitness assessment could be influenced by participant effort and assessor procedures.
Philbois et al., 2022	Allocation concealment was insufficiently described, and the analysis excluded participants whose training adherence was below the specified threshold. A substantial proportion of enrolled participants did not complete follow-up, and the analysis was mainly based on completers, resulting in high risk due to missing outcome data.
Benham et al., 2021	Centralized REDCap randomization and blinded outcome assessment were reported. Some concerns remained because several randomized participants did not complete follow-up, although the analytical approach used available repeated measurements.
Almenning et al., 2015	Computer-generated randomization was conducted independently and trial registration and a study protocol were available. Approximately one fifth of randomized participants did not complete follow-up, and follow-up measurements were not fully blinded, resulting in some concerns.
Guo et al., 2023	Computer randomization was reported, but allocation concealment was not described. Outcome measurements were

	completed by the same investigator without reported blinding, and BIA and estimated cardiorespiratory-fitness testing could be influenced by operator procedures or participant effort. No prospective protocol was identified.
Kazemi et al., 2023	Participants were described as randomly allocated, but sequence-generation and allocation-concealment procedures were insufficiently reported. Several outcomes were estimated using field tests or prediction equations, and the trial registration was retrospective, resulting in some concerns.
Taha et al., 2023	Computer-generated randomization and blinded assessment of objective outcomes were reported, but allocation concealment and outcome-specific availability of all randomized participants were not fully described. The timing and detail of the prospective analysis plan could not be fully verified.
Samhan et al., 2021	Random sequence generation and sequentially numbered, opaque, sealed envelopes were adequately reported, and no substantial missing data were identified. Some concerns remained because VO_2peak testing was partly effort dependent and no prospectively available protocol or detailed analysis plan was identified.
Connolly et al., 2017	Participants were described as randomized, but sequence-generation and allocation-concealment procedures were insufficiently reported. Attrition was limited, although assessor blinding was not reported and some outcomes were effort or operator dependent. No prospective protocol was identified.
Arad et al., 2015	Random sequence generation and allocation concealment were insufficiently described. Eight of 28 randomized participants did not complete the study, and additional outcome-specific data were unavailable. The amount of missing data and completer-based analyses resulted in a high-risk judgement.
Ballesta-García et al., 2020	Block randomization, allocation concealment, blinded assessment, and prospective registration were reported. However, attrition was markedly greater in the control group than in the HIICT group and was addressed using last-observation-carried-forward analysis, resulting in high risk due to missing outcome data.

Supplementary Table S5，Panel B

Note: Risk of bias was assessed using the revised Cochrane Risk of Bias tool for randomized trials (RoB 2; version 22 August 2019), focusing on the effect of assignment to intervention. One conservative summary row was retained for each study. Potential differences across body-composition, cardiorespiratory-fitness, and lipid outcomes were considered when judging missing outcome data, outcome measurement, and selection of the reported result. When the judgement for a domain differed across eligible outcomes within the same study, the less favourable

judgement was retained in the study-level summary. The overall judgement was classified as high risk when at least one domain was judged to be at high risk; as some concerns when no domain was at high risk but at least one domain raised some concerns; and as low risk when all five domains were judged to be at low risk. D1, bias arising from the randomization process; D2, bias due to deviations from intended interventions; D3, bias due to missing outcome data; D4, bias in measurement of the outcome; D5, bias in selection of the reported result; BW, body weight; BMI, body mass index; BF%, body fat percentage; FM, fat mass; VO_2max, maximal oxygen uptake; VO_2peak, peak oxygen uptake; HDL-C, high-density lipoprotein cholesterol; LDL-C, low-density lipoprotein cholesterol; TC, total cholesterol; TG, triglycerides; HIIT, high-intensity interval training; HIICT, high-intensity interval circuit training.

**Study**	**Randomized participants, HIIT/control**	**Completed participants, HIIT/control**	**Dropouts and reasons**	**Supervision**	**Adherence/attendance**	**Adverse events**	**Prescribed training dose**	**Actual completed dose**
Zhang et al., 2015	14/14	12/11	HIIT: 2 withdrew for personal, non-exercise-related reasons. Control: records from 3 participants were invalid because dietary/physical-activity records were not completed.	All sessions were directly supervised by researchers; exercise HR was closely monitored.	94 ± 3%; defined as actual time at the prescribed intensity divided by total prescribed exercise time.	NR	Treadmill HIIT, 4 sessions/week for 12 weeks. Each session included a 10-min warm-up, four 4-min bouts at 85%-95% HRpeak, 3-min walking recovery at 50%-60% HRpeak, a reported 7-min rest, and a 5-min cool-down. Intensity progressed from 85% to 95% HRpeak.	94 ± 3% of the prescribed target-intensity duration.

Smith-Ryan et al., 2016[a]	11/10	10/9	Across the two interval-training arms, 1 participant withdrew after mild syncope following the pre-VO2peak test and 1 did not return after completing 8 of 9 sessions; the report did not assign both withdrawals to a specific interval arm.	One-to-one training under trained research staff in a highly controlled setting; HR and RPE were monitored.	100% among training completers; all 9 sessions completed.	No adverse events were reported during training; 1 participant withdrew after mild syncope following pre-intervention testing.	Cycle HIIT, 3 sessions/week for 3 weeks (9 sessions). The selected 2MIN protocol used five 2-min bouts at undulating 80%-100% VO2peak, with 1-min recovery; total high-intensity work was 10 min/session.	9 of 9 prescribed sessions; 100% adherence among training completers.
Sanayei et al., 2022	11/11	11/9	HIIT + placebo: no loss to follow-up. Placebo: 2 did not complete (1 poor compliance and 1 lost to contact).	HIIT sessions were supervised by an expert trainer.	Regular attendance was stated, but numerical exercise attendance/adherence was NR.	HIIT-specific adverse events were NR. One abdominal cramp occurred in a nonselected Chlorella-only arm.	Running HIIT, 3 sessions/week for 8 weeks (24 scheduled sessions), with 10-min warm-up and 10-min cool-down. Work-bout duration decreased and intensity progressed from 50%-60% to 90%-100% HRmax across the intervention.	NR
Hu et al., 2022	20/20	17/13	HIIT: 3 withdrew for personal reasons. Control: 7	Conducted by professional	NR	No exercise-related adverse events were	Combined-movement HIIT, 5 sessions/week	NR

			withdrew for personal reasons.	coaches/athletic staff; HR was monitored throughout sessions.		reported, including muscle injury, excessive fatigue, syncope, palpitation, angina, abnormal BP responses, nausea, vomiting, or dyspnea.	for 4 weeks. Sessions included a 10-min warm-up and three 9-min high-intensity blocks comprising 9 movements (40-s work/20-s interval), separated by 1-min recovery, targeting approximately 90% HRmax, followed by cool-down/stretching.	
Mohammadi et al., 2023	15/15	14/14	One participant in each group discontinued cooperation and was lost to follow-up.	All sessions were supervised by an international coach.	NR	NR	Running HIIT, 3 sessions/week for 8 weeks. Thirty-second work bouts at approximately 100%-110% maximal aerobic velocity alternated with 30-s active recovery at 50%; 4 laps and 4-6 sets were used, with 5-min recovery between laps and a 15-min warm-up.	NR

Philbois et al., 2022	37/36	25/25	HIIT: 12 discontinued (7 unavailable time, 2 disliked the training method, and 3 pregnancy). Control: 11 discontinued (6 did not answer follow-up calls and 5 unavailable time).	Laboratory sessions were supervised and monitored 3 times/week; HR was recorded continuously.	Only participants with >85% adherence were included in the analysis; exact attendance was NR.	NR	Treadmill HIIT, 3 sessions/week for 16 weeks, after a 2-week adaptation period. Sessions included a 5-min warm-up, repeated 2-min bouts at 85%-90% HR reserve alternating with 3-min recovery at 65%-70% HR reserve, and a 5-min cool-down; total session duration was 35-45 min.	>85% adherence was required for inclusion in the analysis; exact completed sessions were NR.
Benham et al., 2021	16/17	13/16	HIIT: 3 discontinued (2 moved and 1 personal reason). Control: 1 discontinued because a new job increased physical activity.	Three sessions/week: 2 supervised and 1 unsupervised session; unsupervised sessions were tracked using a Polar application/onli	Median HIIT adherence 65% (IQR 51%-85%); first 3 months 78% (55%-97%), final 3 months 44% (41%-69%).	No trial-related adverse events were reported. One unrelated musculoskeletal injury occurred in the nonselected continuous-aerobic-traini ng arm.	Cycle/treadmill/elliptical HIIT, 3 sessions/ week for 26 weeks (78 prescribed sessions). Sessions used a 5-min warm-up and cool-down and 10 cycles of 30 s at 90%HR reserve or RPE 9/10, alternating with 90 s of low-intensity	Median 65% of the 78 prescribed sessions (IQR 51%-85%).

				ne coaching platform.			recovery.	
Almenning et al., 2015	10/10	8/9	HIIT: 2 discontinued (1 pregnancy and 1 withdrawal). Control: 1 discontinued because of work commitments.	An exercise physiologist supervised at least 1 session/week; remaining sessions were self-directed, with HR files reviewed weekly.	90%; 27 ± 1.9 of 30 scheduled sessions.	No adverse events were reported.	Three sessions/week for 10 weeks (30 sessions). Two weekly sessions used 4 x 4 min at 90%-95% HRmax with 3-min active recovery at approximately 70% HRmax; 1 weekly session used 10 x 1-min all-out bouts with 1-min recovery. Exercise mode was self-selected.	27 ± 1.9 of 30 sessions; 90% adherence.
Guo et al., 2023[b]	26/26	23/25	HIIT: 3 withdrew for personal reasons. Control: 1 withdrew for personal reasons.	Sessions were supervised by a sport-science graduate and guided by an aerobics instructor; HR was monitored using Polar H7 devices.	NR	NR	Combined aerobic HIIT, 3 sessions/week for 4 weeks (12 sessions). Sessions included 5-min warm-up and cool-down, 8 movements repeated in 4 rounds, 10-s passive recovery, and	NR

							progressive work bouts of approximately 10-35 s, targeting at least 85% HRmax.	
Kazemi et al., 2023	15/15	15/15	No post-randomization dropout was reported.	All training sessions were supervised by 2 physical-fitness trainers.	NR	NR	Running/walking HIIT, 3 sessions/week for 8 weeks, with at least 48 h between sessions. Each session included a 10-min warm-up, three 3-min bouts at 80%-90% HRmax separated by 3-min active recovery at 55%-65% HRmax, and a 5-min cool-down.	NR
Taha et al., 2023	30/30	25/28	Before intervention, 3 HIIT and 2 control participants withdrew (reasons NR). During follow-up, 2 additional HIIT participants were lost to contact; no additional control loss was reported.	Formal supervision arrangements were NR; HR and RPE were monitored and recorded during sessions.	NR	NR	Cycle HIIT, 3 sessions/week for 12 weeks. Sessions included a 10-min warm-up, four 4-min bouts at 85%-90% peak HR/RPE 15-17 with 3-min active	NR

							recovery at 60%-70%peak HR/ RPE 11-13, and a 10-min cool-down. Intensity progressed after the	
Samhan et al., 2021	30/30	30/30	No post-randomization dropout was reported; 60 women completed the study.	All sessions were supervised by a trained physical therapist; HR was monitored throughout.	NR	No adverse events or complications related to exercise testing or the intervention were reported.	Treadmill HIIT, 3 sessions/week for 8 weeks (24 sessions). Each 38-min session included a 5-min warm-up at 50%-70% HRmax, four 4-min bouts progressing to 75%-90% HRmax, 3-min active recovery at 50%-60% HRmax, and a 5-min cool-down.	NR
Connolly et al., 2017	16/16	15/15	HIIT: 1 withdrew because of a non-study-related musculoskeletal injury. Control: 1 withdrew because of a substantial increase in habitual physical activity.	All sessions were supervised by university sport-and-health staff for safety; HR and RPE were recorded	35 ± 1 of 36 sessions; 2.9 ± 0.1 sessions/week.	One HIIT participant withdrew because of a non-study-related musculoskeletal injury; no participant withdrew because of HIIT intensity.	Self-paced cycle HIIT, 3 sessions/week for 12 weeks. After 5-min warm-up, participants completed five 5-min blocks of repeated 30-s low-, 20-s	35 ± 1 sessions; 2.9 ± 0.1 sessions/wee k; high-intensit y periods

				during every session.			moderate-, and 10-s high-intensity (>90% maximum effort) cycling, separated by 2-min passive recovery, followed by 5-min cool-down; effective interval time was 25 min/session.	reached approximately 94%-95% HRpeak.
Arad et al., 2015	14/14	9/11	HIIT: 5 discontinued (2 disruption caused by Hurricane Sandy and 3 unable to meet time commitments). Control: 3 discontinued (1 time commitment, 1 dissatisfied with no-exercise/weight-management requirements, and 1 relocation).	All sessions were supervised by an exercise physiologist.	Described as high, but a numerical value was NR.	NR	Cycle HIIT, 3 sessions/week for approximately 14 weeks (13-15 weeks when needed). Each 24-min session included 6-min warm-up at 50% HR reserve, four 30-60-s bouts at 75%-90% HR reserve with 180-210-s recovery at 50% HR reserve, and 5-min cool-down; progressive overload was applied.	NR
Ballesta-Garc	18/18	12/17	HIICT: 1 discontinued the	All sessions	Participants were	No adverse events	HIICT, 2	NR

ía et al., 2020			intervention. Control: 6 were lost to follow-up (1 illness and 5 caring for grandchildren).	were directed by a graduate in Physical Activity and Sport Sciences.	required to complete at least 80% of sessions; actual attendance was NR.	occurred in HIICT. Five events occurred in other groups (4 MICT and 1 control), and none occurred during training sessions.	sessions/week for 18 weeks, 60 min/session. A 2-week familiarization period was followed by four progressive 4-week mesocycles; sessions comprised warm-up, a high-speed circuit-training main phase targeting RPE 12-18, and cool-down.	

Supplementary Table S6. Implementation, feasibility, safety, and training-dose characteristics of the included HIIT interventions

Note: Values are presented as HIIT/control for the comparison included in the review. Completed participants refers to the post-intervention/evaluable sample reported by the original study. NR, not reported. Adherence/attendance was retained using the original study definition. Absence of adverse-event reporting was not interpreted as evidence that no adverse event occurred. Prescribed training dose includes exercise mode, intensity, interval/recovery structure, weekly frequency, session duration, intervention duration, and progression when reported. Actual completed dose was recorded only when explicitly reported. [a] For Smith-Ryan et al. (2016), the arm-specific table reported 2MIN n=10 and control n=9 at assessment, whereas the CONSORT diagram reported aggregate analysed counts of 20 interval-training and 10 control participants; the arm-specific values used in this review are shown. [b] For Guo et al. (2023), randomized group sizes were derived from the final sample plus the explicitly reported withdrawals (HIIT 23+3; control 25+1). HIIT, high-intensity interval training; HIICT, high-intensity interval circuit training; MICT, moderate-intensity continuous training; HR, heart rate; HRmax, maximal heart rate; HRpeak, peak heart rate; RPE, rating of perceived exertion; IQR, interquartile range; VO2peak, peak oxygen uptake.

Outcome	Studies excluded because of high overall risk of bias	Primary analysis, k	Primary Hedges' g (95% CI); P	Primary I^2; τ^2	Sensitivity analysis, k	Hedges' g after exclusion (95% CI); P	I^2; τ^2 after exclusion	Interpretation
BW	Hu et al. 2022; Philbois et al. 2022; Arad et al. 2015	11	−0.329 (−0.576 to −0.081); $P = 0.009$	24.23%; 0.0419	8	−0.432 (−0.721 to −0.142); $P = 0.004$	20.87%; 0.0363	The negative direction and statistical significance were maintained. Heterogeneity decreased slightly, supporting the stability of the primary result.
BMI	Hu et al. 2022; Philbois et al. 2022; Arad et al. 2015; Ballesta-García et al. 2020	11	−0.560 (−0.945 to −0.174); $P = 0.005$	67.67%; 0.2810	7	−0.663 (−1.203 to −0.123); $P = 0.016$	72.94%; 0.3797	The negative direction and statistical significance were maintained, although heterogeneity increased after exclusion.
BF%	Hu et al. 2022; Arad et al. 2015	10	−0.762 (−1.354 to −0.169); $P = 0.012$	83.39%; 0.7454	8	−0.579 (−0.923 to −0.234); $P = 0.001$	43.60%; 0.1048	The negative and statistically significant effect was maintained, while heterogeneity decreased substantially. The high-risk studies may have contributed to the heterogeneity, although this cannot be established definitively.
FM	Hu et al. 2022; Arad et al. 2015	8	−0.656 (−1.108 to −0.204); $P = 0.004$	64.04%; 0.2655	6	−0.549 (−0.839 to −0.258); $P < 0.001$	0.00%; 0.0000	The negative direction and statistical significance were maintained, and the estimated heterogeneity was eliminated after exclusion. This suggests that the excluded studies may have contributed to the original heterogeneity, but the finding remains exploratory.
VO_2max/VO_2peak	Philbois et al. 2022; Arad et al. 2015	12	1.085 (0.700 to 1.470); $P < 0.001$	66.48%; 0.2977	10	1.134 (0.662 to 1.607); $P < 0.001$	72.48%; 0.4105	The positive direction and statistical significance were maintained. Heterogeneity increased after exclusion, indicating that the excluded studies were unlikely to account for the observed heterogeneity.

Outcome	Studies excluded because of high overall risk of bias	Primary analysis, k	Primary Hedges' g (95% CI); P	Primary I²; τ²	Sensitivity analysis, k	Hedges' g after exclusion (95% CI); P	I²; τ² after exclusion	Interpretation
HDL-C	Philbois et al. 2022	8	0.843 (0.240 to 1.446); P = 0.006	80.90%; 0.5998	7	1.000 (0.392 to 1.609); P = 0.001	76.06%; 0.5041	The positive direction and statistical significance were maintained. Heterogeneity decreased modestly but remained substantial.
LDL-C	Philbois et al. 2022	8	−0.780 (−1.293 to −0.266); P = 0.003	74.16%; 0.4011	7	−0.904 (−1.439 to −0.370); P < 0.001	69.92%; 0.3617	The negative direction and statistical significance were maintained. Heterogeneity decreased modestly but remained substantial.
TC	Philbois et al. 2022	9	−0.346 (−0.701 to 0.009); P = 0.056	54.23%; 0.1569	8	−0.381 (−0.785 to 0.023); P = 0.065	56.89%; 0.1906	The negative direction and non-significant statistical interpretation were maintained. The heterogeneity estimates increased slightly.
TG	Philbois et al. 2022	9	−0.240 (−0.689 to 0.209); P = 0.295	71.09%; 0.3294	8	−0.282 (−0.790 to 0.225); P = 0.276	72.40%; 0.3837	The negative direction and non-significant statistical interpretation were maintained. Heterogeneity increased slightly and remained substantial.

Supplementary Table S7 Sensitivity analyses excluding studies judged to be at high risk of bias

Table note: Studies judged to be at high overall risk of bias according to the Cochrane Risk of Bias 2 tool were excluded in sensitivity analyses. For each outcome, the effect size and between-study heterogeneity were re-estimated using the same random-effects model with restricted maximum likelihood estimation as in the primary analysis. Negative Hedges' g values indicate effects favouring HIIT for BW, BMI, BF%, FM, LDL-C, TC, and TG, whereas positive values favour HIIT for VO_2max/VO_2peak and HDL-C. Across all nine outcomes, exclusion of high-risk-of-bias studies did not alter the direction or statistical interpretation of the pooled estimates. Changes in heterogeneity were interpreted descriptively and do not establish that the

excluded studies were definitive sources of heterogeneity. No study was removed from the primary analysis solely on the basis of these sensitivity analyses. BW, body weight; BMI, body mass index; BF%, body fat percentage; FM, fat mass; VO_2max/VO_2peak, maximal or peak oxygen uptake; HDL-C, high-density lipoprotein cholesterol; LDL-C, low-density lipoprotein cholesterol; TC, total cholesterol; TG, triglycerides; k, number of studies; CI, confidence interval; I^2, proportion of total variability attributable to between-study heterogeneity; τ^2, estimated between-study variance.

Outcome	Analysis	k	Hedges' g	95% CI	P value	I^2 (%)	τ^2	Interpretation
BW	Primary analysis	11	−0.329	−0.576 to −0.081	0.009	24.23	0.0419	—
BW	Excluding Hu et al. (2022)	10	−0.312	−0.587 to −0.037	0.026	32.09	0.0620	The direction and statistical significance of the pooled effect were maintained. The effect estimate changed only slightly, whereas I^2 and τ^2 increased modestly, providing little indication that Hu et al. (2022) accounted for the heterogeneity in the BW analysis.
BMI	Primary analysis	11	−0.560	−0.945 to −0.174	0.005	67.67	0.2810	—
BMI	Excluding Hu et al. (2022)	10	−0.489	−0.884 to −0.095	0.015	66.28	0.2608	The direction and statistical significance of the pooled effect were maintained, with a modest attenuation in effect magnitude. The heterogeneity estimates changed only slightly, suggesting limited influence on the observed heterogeneity.
BF%	Primary analysis	10	−0.762	−1.354 to −0.169	0.012	83.39	0.7454	—
BF%	Excluding Hu et al. (2022)	9	−0.523	−0.852 to −0.193	0.002	43.63	0.1080	The direction and statistical significance of the pooled effect were maintained, although the magnitude was attenuated. I^2 and τ^2 decreased markedly, suggesting that Hu et al. (2022) may have contributed substantially to the observed heterogeneity.
FM	Primary analysis	8	−0.656	−1.108 to	0.004	64.04	0.2655	—

Outcome	Analysis	k	Hedges' g	95% CI	P value	I² (%)	τ²	Interpretation
				−0.204				
FM	Excluding Hu et al. (2022)	7	−0.471	−0.746 to −0.196	<0.001	0.00	0.0000	The direction and statistical significance of the pooled effect were maintained, although the magnitude was attenuated. Both I² and τ² decreased to zero, suggesting that Hu et al. (2022) may have contributed substantially to the observed heterogeneity in the FM analysis.

Supplementary Table S8. Sensitivity analyses excluding Hu et al. (2022) because of unclear population eligibility

Table note: Primary analyses included all studies contributing data to the corresponding outcome. The sensitivity analyses excluded Hu et al. (2022), which enrolled women with normal-weight obesity and therefore did not clearly meet conventional BMI-based definitions of overweight or obesity. Negative Hedges' g values indicate effects favouring HIIT for all four outcomes. All estimates were calculated using random-effects models, with between-study variance estimated using the restricted maximum likelihood method. P values refer to tests of the pooled effects. The sensitivity analyses were exploratory. Marked reductions in I^2 or τ^2 after exclusion of a single study suggest a possible contribution to between-study heterogeneity but do not establish a definitive or sole source of heterogeneity. BW, body weight; BMI, body mass index; BF%, body fat percentage; FM, fat mass; CI, confidence interval; HIIT, high-intensity interval training; REML, restricted maximum likelihood.

Study	Population and clinical status	Original adiposity eligibility criterion	Applicability to the retained sample and data extraction	Review decision and rationale
Zhang et al., 2015	Chinese female university students with overweight or pre-obesity	BMI ≥25 kg/m² and BF% ≥28%	The criteria applied to all participants in the retained comparison. Data from the HIIT and non-training control arms were extracted.	**Clearly eligible.** The original study reported explicit BMI- and body-fat-based eligibility criteria.
Smith-Ryan et al., 2016	Sedentary women with overweight or obesity	BMI >25 kg/m²	The criterion applied to all participants in the retained comparison. Data from the 2MIN HIIT and non-exercise control arms were extracted; the 1MIN arm classified as SIT was not retained.	**Clearly eligible.** The original study reported an explicit BMI-based eligibility criterion.
Sanayei et al., 2022	Women with overweight or obesity	BMI 25–35 kg/m²	The criterion applied to all participants in the retained comparison. The placebo-matched comparison of HIIT plus placebo versus placebo alone was retained.	**Clearly eligible.** The original study reported an explicit BMI-based eligibility criterion.
Hu et al., 2022	Female university students with normal-weight obesity	BMI 18.5–24.9 kg/m² combined with BF% >30%	The criteria applied to all participants in the retained comparison, and the complete HIIT and control samples were extracted.	**Eligible under the prespecified adiposity-based definition of normal-weight obesity.** The potential influence of this phenotype was additionally examined in a separate sensitivity analysis.
Mohammadi et al., 2023	Women with PCOS	NR	The complete HIIT and control samples were extracted. No separately reported subgroup meeting an explicit overweight, obesity, or adiposity criterion was available.	**Conventional overweight or obesity eligibility unclear.** The study was retained in the primary analysis and classified for exclusion in the population-eligibility sensitivity analysis.
Philbois et al., 2022	Women with PCOS	NR	Data from the HIIT and non-exercise control arms were extracted; the MICT arm was not retained. No separately reported adiposity-defined subgroup was	**Conventional overweight or obesity eligibility unclear.** The study was retained in the primary analysis and classified for exclusion in the

Study	Population and clinical status	Original adiposity eligibility criterion	Applicability to the retained sample and data extraction	Review decision and rationale
			available.	population-eligibility sensitivity analysis.
Benham et al., 2021	Women with PCOS	NR	Data from the HIIT and non-exercise control arms were extracted; the continuous aerobic exercise arm was not retained. No separately reported adiposity-defined subgroup was available.	**Conventional overweight or obesity eligibility unclear.** The study was retained in the primary analysis and classified for exclusion in the population-eligibility sensitivity analysis.
Almenning et al., 2015	Women with PCOS	NR	Data from the HIIT and control arms were extracted; the strength-training arm was not retained. No separately reported adiposity-defined subgroup was available.	**Conventional overweight or obesity eligibility unclear.** The study was retained in the primary analysis and classified for exclusion in the population-eligibility sensitivity analysis.
Guo et al., 2023	Young female university students with overweight or obesity	BMI ≥25 kg/m² or BF% ≥30%	The criterion applied to all participants in the retained comparison, and the complete HIIT and control samples were extracted.	**Clearly eligible.** The original study reported an explicit BMI- or body-fat-based eligibility criterion.
Kazemi et al., 2023	Postmenopausal women with metabolic syndrome	NR as a mandatory overweight or obesity criterion	Data from the HIIT and control arms were extracted; the resistance-training arm was not retained. No separately reported adiposity-defined subgroup was available.	**Conventional overweight or obesity eligibility unclear.** Metabolic-syndrome status alone did not establish that every participant met an explicit overweight or obesity criterion.
Taha et al., 2023	Women with obesity and stable hypertension	The BMI criterion was reported as 0–39.9 kg/m² in the original article; the lower bound was unclear as printed	The complete HIIT and control samples were extracted. No separately reported adiposity-defined subgroup was available.	**Exact conventional eligibility criterion unclear.** Although the population was described as women with obesity, the printed BMI criterion could not be verified without inference.
Samhan et al., 2021	Female breast cancer survivors with overweight or obesity	BMI >25 kg/m²	The criterion applied to all participants in the retained comparison, and the complete HIIT and	**Clearly eligible.** The original study reported an explicit BMI-based eligibility criterion.

Study	Population and clinical status	Original adiposity eligibility criterion	Applicability to the retained sample and data extraction	Review decision and rationale
			control samples were extracted.	
Connolly et al., 2017	Inactive premenopausal women	NR	Data from the HIIT and non-exercise control arms were extracted; the continuous-training arm was not retained. No separately reported adiposity-defined subgroup was available.	**Conventional overweight or obesity eligibility unclear.** The study was retained in the primary analysis and classified for exclusion in the population-eligibility sensitivity analysis.
Arad et al., 2015	Sedentary, non-diabetic, premenopausal African-American women with overweight or obesity	BMI >25 kg/m²	The criterion applied to all participants in the retained comparison, and the complete HIIT and control samples were extracted.	**Clearly eligible.** The original study reported an explicit BMI-based eligibility criterion.
Ballesta-García et al., 2020	Middle-aged and older women	NR	Data from the HIICT and non-exercise control arms were extracted; the MICT arm was not retained. No separately reported adiposity-defined subgroup was available.	**Conventional overweight or obesity eligibility unclear.** The study was retained in the primary analysis and classified for exclusion in the population-eligibility sensitivity analysis.

Supplementary Table S9. Population eligibility and data-extraction decisions for the included studies

Note: NR, not reported; BMI, body mass index; BF%, body fat percentage; PCOS, polycystic ovary syndrome; HIIT, high-intensity interval training; SIT, sprint interval training; MICT, moderate-intensity continuous training; HIICT, high-intensity interval circuit training. Population eligibility was assessed from the adiposity-related eligibility criteria reported in the original trial publications. A group-level mean BMI was not considered sufficient evidence that every participant met conventional overweight or obesity criteria. Conventional eligibility was classified as unclear when no explicit individual-level BMI or body-fat threshold was reported or when the reported criterion could not be verified without inference. NR indicates that an explicit criterion was not reported or could not be verified from the available report; it does not necessarily indicate that no criterion was applied by the original investigators. Hu et al. (2022) was not classified as having unclear eligibility because normal-weight obesity was explicitly defined using a normal-range BMI combined with an elevated body-fat criterion. The BMI criterion for Taha et al.

(2023) is reproduced as printed in the original article and was not corrected by inference. These review-level classifications were used for sensitivity analyses and did not result in retrospective removal of studies from the primary analyses.

Outcome	Analysis	k	Hedges' g	95% CI	P value	I^2	τ^2	Cautious interpretation
BW	Primary analysis	11	−0.329	−0.576 to −0.081	0.0093	24.23%	0.0419	The pooled effect significantly favoured HIIT, with low between-study heterogeneity.
BW	Excluding studies with unclear population eligibility	5	−0.164	−0.474 to 0.146	0.3005	0.00%	0.0000	The direction remained favourable to HIIT, but the effect was attenuated and statistical significance was not retained, indicating sensitivity to population-eligibility assumptions.
BMI	Primary analysis	11	−0.560	−0.945 to −0.174	0.0045	67.67%	0.2810	The pooled effect significantly favoured HIIT, although substantial heterogeneity was present.
BMI	Excluding studies with unclear population eligibility	4	−0.440	−1.089 to 0.208	0.1834	69.26%	0.2990	The direction remained favourable to HIIT, but statistical significance was not retained and heterogeneity remained substantial.
BF%	Primary analysis	10	−0.762	−1.354 to −0.169	0.0118	83.39%	0.7454	The pooled effect significantly favoured HIIT, with considerable heterogeneity.
BF%	Excluding studies with unclear population eligibility	7	−0.833	−1.709 to 0.044	0.0626	89.39%	1.2275	The direction remained favourable to HIIT and the absolute point estimate increased slightly; however, the confidence interval widened, statistical significance was not retained, and heterogeneity increased.
FM	Primary analysis	8	−0.656	−1.108 to −0.204	0.0044	64.04%	0.2655	The pooled effect significantly favoured HIIT, although substantial heterogeneity was present.
FM	Excluding studies with unclear population	5	−0.793	−1.568 to −0.018	0.0450	80.14%	0.6163	The direction and statistical significance were retained, but precision decreased and heterogeneity increased considerably.

Outcome	Analysis	k	Hedges' g	95% CI	P value	I²	τ²	Cautious interpretation
	eligibility							
VO_2max/VO_2peak	Primary analysis	12	1.085	0.700 to 1.470	<0.001	66.48%	0.2977	The pooled effect significantly favoured HIIT, although substantial heterogeneity was present.
VO_2max/VO_2peak	Excluding studies with unclear population eligibility	6	1.298	0.710 to 1.887	<0.001	69.33%	0.3636	The direction and statistical significance were retained, and the point estimate increased; however, heterogeneity was not reduced.
TC	Primary analysis	9	−0.346	−0.701 to 0.009	0.0562	54.23%	0.1569	The pooled estimate was negative but did not reach statistical significance, with moderate heterogeneity.
TC	Excluding studies with unclear population eligibility	2	0.345	−0.241 to 0.931	0.2486	0.00%	0.0000	Statistical non-significance was retained, but the point estimate changed from negative to positive. Only two studies remained, making the result highly exploratory.
TG	Primary analysis	9	−0.240	−0.689 to 0.209	0.2949	71.09%	0.3294	The pooled estimate was negative but statistically non-significant, with substantial heterogeneity.
TG	Excluding studies with unclear population eligibility	2	−0.041	−0.622 to 0.541	0.8909	0.00%	0.0000	The direction and non-significant interpretation remained unchanged, but the estimate approached the null. Only two studies remained, making the result highly exploratory.

Supplementary Table S10. Summary of sensitivity analyses excluding studies with unclear population eligibility

Note: The primary analyses included all studies retained for each outcome. The sensitivity analyses excluded studies classified as having unclear conventional overweight or obesit y eligibility according to the review-level assessment presented in Supplementary Table S9. Negative Hedges' g values favour HIIT for BW, BMI, BF%, FM, TC, and TG, where as positive values favour HIIT for VO_2max/VO_2peak. HDL-C and LDL-C were not quantitatively pooled in this sensitivity analysis because only one study remained for each out

come after studies with unclear population eligibility were excluded. Results based on two studies should be considered highly exploratory, and an I^2 value of 0% should not be interpreted as evidence of confirmed homogeneity. Changes between the primary and sensitivity analyses were interpreted descriptively; no formal statistical comparison between t he two pooled estimates was conducted. All pooled estimates were calculated using random-effects models with between-study variance estimated by the restricted maximum-likeli hood method. BW, body weight; BMI, body mass index; BF%, body fat percentage; FM, fat mass; VO_2max/VO_2peak, maximal or peak oxygen uptake; TC, total cholesterol; TG, triglycerides; HIIT, high-intensity interval training; CI, confidence interval.

```
meta bias, egger

  Effect-size label: Hedges's g
        Effect size: _meta_es
          Std. err.: _meta_se

Regression-based Egger test for small-study effects
Random-effects model
Method: REML

H0: beta1 = 0; no small-study effects
            beta1 =      0.02
      SE of beta1 =     2.169
                z =      0.01
       Prob > |z| =    0.9944

.
```

Supplementary Fig. 1. Egger's regression plot for body weight.

```
meta bias, egger

  Effect-size label: Hedges's g
        Effect size: _meta_es
          Std. err.: _meta_se

Regression-based Egger test for small-study effects
Random-effects model
Method: REML

H0: beta1 = 0; no small-study effects
            beta1 =     -4.00
      SE of beta1 =     2.824
                z =     -1.42
       Prob > |z| =    0.1562
```

Supplementary Fig. 2. Egger's regression plot for BMI.

```
meta bias, egger

  Effect-size label: Hedges's g
        Effect size: _meta_es
          Std. err.: _meta_se

Regression-based Egger test for small-study effects
Random-effects model
Method: REML

H0: beta1 = 0; no small-study effects
            beta1 =     -5.41
      SE of beta1 =     3.439
                z =     -1.57
       Prob > |z| =    0.1155
```

Supplementary Fig. 3. Egger's regression plot for body fat percentage.

```
meta bias, egger

  Effect-size label: Hedges's g
        Effect size: _meta_es
          Std. err.: _meta_se

Regression-based Egger test for small-study effects
Random-effects model
Method: REML

H0: beta1 = 0; no small-study effects
            beta1 =     -2.34
      SE of beta1 =     3.561
                z =     -0.66
       Prob > |z| =    0.5117
```

Supplementary Fig. 4. Egger's regression plot for fat mass.

```
meta bias, egger

  Effect-size label: Hedges's g
        Effect size: _meta_es
          Std. err.: _meta_se

Regression-based Egger test for small-study effects
Random-effects model
Method: REML

H0: beta1 = 0; no small-study effects
            beta1 =       2.38
      SE of beta1 =      2.528
                z =       0.94
       Prob > |z| =     0.3454
```

Supplementary Fig. 5. Egger’s regression plot for maximal or peak oxygen uptake.

```
meta bias, egger

  Effect-size label: Hedges's g
        Effect size: _meta_es
          Std. err.: _meta_se

Regression-based Egger test for small-study effects
Random-effects model
Method: REML

H0: beta1 = 0; no small-study effects
            beta1 =       5.88
      SE of beta1 =      3.590
                z =       1.64
       Prob > |z| =     0.1014
```

Supplementary Fig. 6. Egger’s regression plot for high-density lipoprotein cholesterol.

```
meta bias, egger

  Effect-size label: Hedges's g
        Effect size: _meta_es
          Std. err.: _meta_se

Regression-based Egger test for small-study effects
Random-effects model
Method: REML

H0: beta1 = 0; no small-study effects
            beta1 =      1.02
      SE of beta1 =     5.083
                z =      0.20
       Prob > |z| =    0.8404

.
```

Supplementary Fig. 7. Egger's regression plot for low-density lipoprotein cholesterol.

```
meta bias, egger

  Effect-size label: Hedges's g
        Effect size: _meta_es
          Std. err.: _meta_se

Regression-based Egger test for small-study effects
Random-effects model
Method: REML

H0: beta1 = 0; no small-study effects
            beta1 =      3.77
      SE of beta1 =     3.014
                z =      1.25
       Prob > |z| =    0.2116
```

Supplementary Fig. 8. Egger's regression plot for total cholesterol.

```
. meta bias, egger

  Effect-size label: Hedges's g
        Effect size: _meta_es
          Std. err.: _meta_se

Regression-based Egger test for small-study effects
Random-effects model
Method: REML

H0: beta1 = 0; no small-study effects
            beta1 =      3.77
      SE of beta1 =     3.850
                z =      0.98
       Prob > |z| =    0.3273

.
```

Supplementary Fig. 9. Egger’s regression plot for triglycerides.

```
meta regress age, random(reml)

  Effect-size label: Hedges's g
        Effect size: _meta_es
          Std. err.: _meta_se

Random-effects meta-regression                      Number of obs  =         11
Method: REML                                        Residual heterogeneity:
                                                                tau2 =  .05164
                                                              I2 (%) =   27.70
                                                                  H2 =    1.38
                                                       R-squared (%) =    0.00
                                                    Wald chi2(1)     =    0.93
                                                    Prob > chi2      =  0.3338

    _meta_es   Coefficient  Std. err.      z    P>|z|     [95% conf. interval]

         age    -.0108571    .011233   -0.97   0.334    -.0328734    .0111591
       _cons     .0356231   .3990277    0.09   0.929    -.7464569    .8177031

Test of residual homogeneity: Q_res = chi2(9) = 12.85    Prob > Q_res = 0.1694
```

```
meta regress baselinebmi, random(reml)

  Effect-size label: Hedges's g
        Effect size: _meta_es
          Std. err.: _meta_se

Random-effects meta-regression                      Number of obs  =         11
Method: REML                                        Residual heterogeneity:
                                                                tau2 =  .04967
                                                              I2 (%) =   26.90
                                                                  H2 =    1.37
                                                       R-squared (%) =    0.00
                                                    Wald chi2(1)     =    1.15
                                                    Prob > chi2      =  0.2843

    _meta_es   Coefficient  Std. err.      z    P>|z|     [95% conf. interval]

 baselinebmi     .0425935   .0397837    1.07   0.284    -.0353811    .1205681
       _cons    -1.554563   1.152069   -1.35   0.177    -3.812577    .7034507

Test of residual homogeneity: Q_res = chi2(9) = 12.63    Prob > Q_res = 0.1802
```

```
meta regress interventionperiod, random(reml)

  Effect-size label: Hedges's g
        Effect size: _meta_es
          Std. err.: _meta_se

Random-effects meta-regression                      Number of obs  =         11
Method: REML                                        Residual heterogeneity:
                                                                tau2 =  .01508
                                                              I2 (%) =   10.23
                                                                  H2 =    1.11
                                                       R-squared (%) =   64.02
                                                    Wald chi2(1)     =    2.56
                                                    Prob > chi2      =  0.1097

          _meta_es   Coefficient  Std. err.      z    P>|z|     [95% conf. interval]

interventionperiod      .036086   .0225592    1.60   0.110    -.0081292    .0803012
             _cons    -.7325993   .2788019   -2.63   0.009    -1.279041   -.1861577

Test of residual homogeneity: Q_res = chi2(9) = 11.18    Prob > Q_res = 0.2638
```

```
meta regress trainingfrequency, random(reml)

  Effect-size label: Hedges's g
        Effect size: _meta_es
          Std. err.: _meta_se

Random-effects meta-regression                      Number of obs  =         11
Method: REML                                        Residual heterogeneity:
                                                                tau2 =  .06161
                                                              I2 (%) =   31.96
                                                                  H2 =    1.47
                                                       R-squared (%) =    0.00
                                                    Wald chi2(1)     =    0.17
                                                    Prob > chi2      =  0.6830

         _meta_es   Coefficient  Std. err.      z    P>|z|     [95% conf. interval]

trainingfrequency     -.088812   .2175014   -0.41   0.683    -.5151069    .3374829
            _cons    -.0395424    .722192   -0.05   0.956    -1.455013    1.375928

Test of residual homogeneity: Q_res = chi2(9) = 13.76    Prob > Q_res = 0.1313
```

Supplementary Fig. 10. Regression analysis plot for body weight.

```
meta regress age, random(reml)

  Effect-size label: Hedges's g
        Effect size: _meta_es
          Std. err.: _meta_se

Random-effects meta-regression                    Number of obs  =         11
Method: REML                                      Residual heterogeneity:
                                                              tau2 =   .3375
                                                            I2 (%) =   71.36
                                                                H2 =    3.49
                                                     R-squared (%) =    0.00
                                                  Wald chi2(1)     =    0.06
                                                  Prob > chi2      =  0.8064

    _meta_es | Coefficient  Std. err.      z    P>|z|     [95% conf. interval]
-------------+----------------------------------------------------------------
         age |  -.0034668   .0141451    -0.25   0.806    -.0311906    .0242571
       _cons |  -.4379055   .5525989    -0.79   0.428    -1.520979    .6451684

Test of residual homogeneity: Q_res = chi2(9) = 28.51    Prob > Q_res = 0.0008
```

```
meta regress baselinebmi, random(reml)

  Effect-size label: Hedges's g
        Effect size: _meta_es
          Std. err.: _meta_se

Random-effects meta-regression                    Number of obs  =         11
Method: REML                                      Residual heterogeneity:
                                                              tau2 =   .1199
                                                            I2 (%) =   46.60
                                                                H2 =    1.87
                                                     R-squared (%) =   57.33
                                                  Wald chi2(1)     =    6.00
                                                  Prob > chi2      =  0.0143

    _meta_es | Coefficient  Std. err.      z    P>|z|     [95% conf. interval]
-------------+----------------------------------------------------------------
 baselinebmi |   .1164213    .047513     2.45   0.014     .0232976    .2095451
       _cons |  -3.906529   1.382483    -2.83   0.005    -6.616145   -1.196912

Test of residual homogeneity: Q_res = chi2(9) = 17.45    Prob > Q_res = 0.0421
```

```
meta regress interventionperiod, random(reml)

  Effect-size label: Hedges's g
        Effect size: _meta_es
          Std. err.: _meta_se

Random-effects meta-regression                    Number of obs  =         11
Method: REML                                      Residual heterogeneity:
                                                              tau2 =   .1804
                                                            I2 (%) =   57.07
                                                                H2 =    2.33
                                                     R-squared (%) =   35.82
                                                  Wald chi2(1)     =    4.45
                                                  Prob > chi2      =  0.0350

          _meta_es | Coefficient  Std. err.      z    P>|z|     [95% conf. interval]
-------------------+----------------------------------------------------------------
interventionperiod |   .0674098   .0319678     2.11   0.035     .0047541    .1300655
             _cons |  -1.376439   .4278623    -3.22   0.001    -2.215034   -.5378441

Test of residual homogeneity: Q_res = chi2(9) = 21.27    Prob > Q_res = 0.0115
```

```
meta regress trainingfrequency, random(reml)

  Effect-size label: Hedges's g
        Effect size: _meta_es
          Std. err.: _meta_se

Random-effects meta-regression                    Number of obs  =         11
Method: REML                                      Residual heterogeneity:
                                                              tau2 =   .2547
                                                            I2 (%) =   65.73
                                                                H2 =    2.92
                                                     R-squared (%) =    9.39
                                                  Wald chi2(1)     =    1.48
                                                  Prob > chi2      =  0.2233

         _meta_es | Coefficient  Std. err.      z    P>|z|     [95% conf. interval]
------------------+----------------------------------------------------------------
trainingfrequency |  -.3269655   .2685139    -1.22   0.223     -.853243     .199312
            _cons |   .4785695   .8719898     0.55   0.583    -1.230499    2.187638

Test of residual homogeneity: Q_res = chi2(9) = 24.11    Prob > Q_res = 0.0041
```

Supplementary Fig. 11. Regression analysis plot for body mass index.

```
. meta regress age, random(reml)

  Effect-size label: Hedges's g
        Effect size: _meta_es
          Std. err.: _meta_se

Random-effects meta-regression                      Number of obs  =        10
Method: REML                                        Residual heterogeneity:
                                                                tau2 =   .7777
                                                              I2 (%) =   82.86
                                                                  H2 =    5.84
                                                       R-squared (%) =    0.00
                                                    Wald chi2(1)     =    1.14
                                                    Prob > chi2      =  0.2856

    _meta_es | Coefficient  Std. err.      z    P>|z|     [95% conf. interval]
-------------+-----------------------------------------------------------------
         age |   .0332711   .0311583     1.07   0.286    -.0277979    .0943402
       _cons |  -1.768864   .9910231    -1.78   0.074    -3.711233    .1735061

Test of residual homogeneity: Q_res = chi2(8) = 38.08     Prob > Q_res = 0.0000
```

```
meta regress baselinebmi, random(reml)

  Effect-size label: Hedges's g
        Effect size: _meta_es
          Std. err.: _meta_se

Random-effects meta-regression                      Number of obs  =        10
Method: REML                                        Residual heterogeneity:
                                                                tau2 =   .6032
                                                              I2 (%) =   79.47
                                                                  H2 =    4.87
                                                       R-squared (%) =   19.08
                                                    Wald chi2(1)     =    3.34
                                                    Prob > chi2      =  0.0676

    _meta_es | Coefficient  Std. err.      z    P>|z|     [95% conf. interval]
-------------+-----------------------------------------------------------------
 baselinebmi |   .1509771   .0826031     1.83   0.068    -.0109221    .3128763
       _cons |   -5.04668   2.364222    -2.13   0.033    -9.680469   -.4128905

Test of residual homogeneity: Q_res = chi2(8) = 35.52     Prob > Q_res = 0.0000
```

```
meta regress interventionperiod, random(reml)

  Effect-size label: Hedges's g
        Effect size: _meta_es
          Std. err.: _meta_se

Random-effects meta-regression                      Number of obs  =        10
Method: REML                                        Residual heterogeneity:
                                                                tau2 =   .7825
                                                              I2 (%) =   83.93
                                                                  H2 =    6.22
                                                       R-squared (%) =    0.00
                                                    Wald chi2(1)     =    0.96
                                                    Prob > chi2      =  0.3279

          _meta_es | Coefficient  Std. err.      z    P>|z|     [95% conf. interval]
-------------------+-----------------------------------------------------------------
interventionperiod |   .0850172   .0868939     0.98   0.328    -.0852917    .2553261
             _cons |  -1.470213   .7857925    -1.87   0.061    -3.010338    .0699121

Test of residual homogeneity: Q_res = chi2(8) = 37.33     Prob > Q_res = 0.0000
```

```
meta regress trainingfrequency, random(reml)

  Effect-size label: Hedges's g
        Effect size: _meta_es
          Std. err.: _meta_se

Random-effects meta-regression                      Number of obs  =        10
Method: REML                                        Residual heterogeneity:
                                                                tau2 =  .08139
                                                              I2 (%) =   36.51
                                                                  H2 =    1.58
                                                       R-squared (%) =   89.08
                                                    Wald chi2(1)     =   21.37
                                                    Prob > chi2      =  0.0000

         _meta_es | Coefficient  Std. err.      z    P>|z|     [95% conf. interval]
------------------+-----------------------------------------------------------------
trainingfrequency |  -1.356751   .2935036    -4.62   0.000    -1.932008   -.7814947
            _cons |    3.64555   .9516103     3.83   0.000     1.780428    5.510671

Test of residual homogeneity: Q_res = chi2(8) = 12.51     Prob > Q_res = 0.1298
```

Supplementary Fig. 12. Regression analysis plot for body fat percentage.

```
meta regress age, random(reml)

  Effect-size label: Hedges's g
        Effect size: _meta_es
          Std. err.: _meta_se

Random-effects meta-regression                      Number of obs  =          8
Method: REML                                        Residual heterogeneity:
                                                                tau2 =   .2598
                                                              I2 (%) =   63.07
                                                                  H2 =    2.71
                                                       R-squared (%) =    2.12
                                                    Wald chi2(1)     =    1.33
                                                    Prob > chi2      =  0.2481

    _meta_es   Coefficient  Std. err.      z    P>|z|     [95% conf. interval]

         age     .0211342      .0183     1.15   0.248    -.0147332    .0570017
       _cons    -1.423437   .7030964    -2.02   0.043    -2.801481   -.0453938

Test of residual homogeneity: Q_res = chi2(6) = 15.05     Prob > Q_res = 0.0199
```

```
meta regress baselinebmi, random(reml)

  Effect-size label: Hedges's g
        Effect size: _meta_es
          Std. err.: _meta_se

Random-effects meta-regression                      Number of obs  =          8
Method: REML                                        Residual heterogeneity:
                                                                tau2 =   .1295
                                                              I2 (%) =   45.19
                                                                  H2 =    1.82
                                                       R-squared (%) =   51.21
                                                    Wald chi2(1)     =    5.07
                                                    Prob > chi2      =  0.0243

    _meta_es   Coefficient  Std. err.      z    P>|z|     [95% conf. interval]

 baselinebmi     .1186486   .0526954     2.25   0.024     .0153675    .2219298
       _cons    -4.032425   1.515599    -2.66   0.008    -7.002945   -1.061905

Test of residual homogeneity: Q_res = chi2(6) = 10.98     Prob > Q_res = 0.0891
```

```
meta regress interventionperiod, random(reml)

  Effect-size label: Hedges's g
        Effect size: _meta_es
          Std. err.: _meta_se

Random-effects meta-regression                      Number of obs  =          8
Method: REML                                        Residual heterogeneity:
                                                                tau2 =   .1522
                                                              I2 (%) =   51.17
                                                                  H2 =    2.05
                                                       R-squared (%) =   42.67
                                                    Wald chi2(1)     =    3.39
                                                    Prob > chi2      =  0.0658

          _meta_es   Coefficient  Std. err.      z    P>|z|     [95% conf. interval]

interventionperiod     .1039081   .0564722     1.84   0.066    -.0067753    .2145915
             _cons    -1.578247     .54212    -2.91   0.004    -2.640782   -.5157108

Test of residual homogeneity: Q_res = chi2(6) = 12.19     Prob > Q_res = 0.0578
```

```
meta regress trainingfrequency, random(reml)

  Effect-size label: Hedges's g
        Effect size: _meta_es
          Std. err.: _meta_se

Random-effects meta-regression                      Number of obs  =          8
Method: REML                                        Residual heterogeneity:
                                                                tau2 = 1.4e-07
                                                              I2 (%) =    0.00
                                                                  H2 =    1.00
                                                       R-squared (%) =  100.00
                                                    Wald chi2(1)     =   14.00
                                                    Prob > chi2      =  0.0002

         _meta_es   Coefficient  Std. err.      z    P>|z|     [95% conf. interval]

trainingfrequency    -.8371747   .2237729    -3.74   0.000    -1.275762   -.3985878
            _cons     2.111305   .7433612     2.84   0.005      .654344    3.568266

Test of residual homogeneity: Q_res = chi2(6) =  3.74     Prob > Q_res = 0.7122
```

Supplementary Fig. 13. Regression analysis plot for fat mass.

```
meta regress age, random(reml)

  Effect-size label: Hedges's g
        Effect size: _meta_es
          Std. err.: _meta_se

Random-effects meta-regression                    Number of obs  =         12
Method: REML                                      Residual heterogeneity:
                                                              tau2 =    .2469
                                                            I2 (%) =    61.46
                                                                H2 =     2.59
                                                     R-squared (%) =    17.05
                                                  Wald chi2(1)     =     2.86
                                                  Prob > chi2      =   0.0909

------------------------------------------------------------------------------
    _meta_es | Coefficient  Std. err.      z    P>|z|     [95% conf. interval]
-------------+----------------------------------------------------------------
         age |  -.0284094   .0168031    -1.69   0.091    -.0613429    .0045241
       _cons |   2.034579   .5928696     3.43   0.001     .8725763    3.196583
------------------------------------------------------------------------------
Test of residual homogeneity: Q_res = chi2(10) = 25.41   Prob > Q_res = 0.0046
```

```
meta regress baselinebmi, random(reml)

  Effect-size label: Hedges's g
        Effect size: _meta_es
          Std. err.: _meta_se

Random-effects meta-regression                    Number of obs  =         12
Method: REML                                      Residual heterogeneity:
                                                              tau2 =     .286
                                                            I2 (%) =    64.80
                                                                H2 =     2.84
                                                     R-squared (%) =     3.93
                                                  Wald chi2(1)     =     1.71
                                                  Prob > chi2      =   0.1910

------------------------------------------------------------------------------
    _meta_es | Coefficient  Std. err.      z    P>|z|     [95% conf. interval]
-------------+----------------------------------------------------------------
 baselinebmi |  -.0969155   .0741087    -1.31   0.191    -.2421659    .0483348
       _cons |   3.905761   2.166075     1.80   0.071    -.3396682     8.15119
------------------------------------------------------------------------------
Test of residual homogeneity: Q_res = chi2(10) = 27.58   Prob > Q_res = 0.0021
```

```
meta regress interventionperiod, random(reml)

  Effect-size label: Hedges's g
        Effect size: _meta_es
          Std. err.: _meta_se

Random-effects meta-regression                    Number of obs  =         12
Method: REML                                      Residual heterogeneity:
                                                              tau2 =    .2599
                                                            I2 (%) =    63.07
                                                                H2 =     2.71
                                                     R-squared (%) =    12.69
                                                  Wald chi2(1)     =     1.58
                                                  Prob > chi2      =   0.2081

-------------------------------------------------------------------------------------
           _meta_es | Coefficient  Std. err.      z    P>|z|     [95% conf. interval]
--------------------+----------------------------------------------------------------
 interventionperiod |  -.0428307   .0340262    -1.26   0.208    -.1095208    .0238594
              _cons |   1.537379   .4069718     3.78   0.000     .7397292    2.335029
-------------------------------------------------------------------------------------
Test of residual homogeneity: Q_res = chi2(10) = 25.25   Prob > Q_res = 0.0049
```

```
meta regress trainingfrequency, random(reml)

  Effect-size label: Hedges's g
        Effect size: _meta_es
          Std. err.: _meta_se

Random-effects meta-regression                    Number of obs  =         12
Method: REML                                      Residual heterogeneity:
                                                              tau2 =    .1811
                                                            I2 (%) =    55.86
                                                                H2 =     2.27
                                                     R-squared (%) =    39.18
                                                  Wald chi2(1)     =     5.34
                                                  Prob > chi2      =   0.0209

------------------------------------------------------------------------------------
          _meta_es | Coefficient  Std. err.      z    P>|z|     [95% conf. interval]
-------------------+----------------------------------------------------------------
 trainingfrequency |   1.677058   .7258587     2.31   0.021     .2544008    3.099715
             _cons |  -4.049803    2.22576    -1.82   0.069    -8.412213    .3126063
------------------------------------------------------------------------------------
Test of residual homogeneity: Q_res = chi2(10) = 22.18   Prob > Q_res = 0.0142
```

Supplementary Fig. 14. Regression analysis plot for maximal or peak oxygen uptake.

```
meta regress age, random(reml)

  Effect-size label: Hedges's g
        Effect size: _meta_es
          Std. err.: _meta_se

Random-effects meta-regression                    Number of obs  =          8
Method: REML                                      Residual heterogeneity:
                                                              tau2 =   .1189
                                                            I2 (%) =   45.11
                                                                H2 =    1.82
                                                     R-squared (%) =   80.18
                                                  Wald chi2(1)     =   13.04
                                                  Prob > chi2      =  0.0003

    _meta_es   Coefficient  Std. err.      z    P>|z|     [95% conf. interval]

         age      .0627931   .0173923    3.61   0.000     .0287049    .0968813
       _cons     -1.492693   .6558343   -2.28   0.023    -2.778104   -.2072812

Test of residual homogeneity: Q_res = chi2(6) = 10.99   Prob > Q_res = 0.0888
```

```
meta regress baselinebmi, random(reml)

  Effect-size label: Hedges's g
        Effect size: _meta_es
          Std. err.: _meta_se

Random-effects meta-regression                    Number of obs  =          8
Method: REML                                      Residual heterogeneity:
                                                              tau2 =    .735
                                                            I2 (%) =   83.23
                                                                H2 =    5.96
                                                     R-squared (%) =    0.00
                                                  Wald chi2(1)     =    0.01
                                                  Prob > chi2      =  0.9149

    _meta_es   Coefficient  Std. err.      z    P>|z|     [95% conf. interval]

 baselinebmi     -.0137735   .1288495   -0.11   0.915    -.2663138    .2387668
       _cons      1.257078   3.839197    0.33   0.743    -6.267611    8.781766

Test of residual homogeneity: Q_res = chi2(6) = 34.56   Prob > Q_res = 0.0000
```

```
meta regress interventionperiod, random(reml)

  Effect-size label: Hedges's g
        Effect size: _meta_es
          Std. err.: _meta_se

Random-effects meta-regression                    Number of obs  =          8
Method: REML                                      Residual heterogeneity:
                                                              tau2 =   .4228
                                                            I2 (%) =   75.13
                                                                H2 =    4.02
                                                     R-squared (%) =   29.51
                                                  Wald chi2(1)     =    3.08
                                                  Prob > chi2      =  0.0792

          _meta_es   Coefficient  Std. err.      z    P>|z|     [95% conf. interval]

interventionperiod     -.0812775   .0462981   -1.76   0.079    -.1720202    .0094652
             _cons      1.801343   .6144752    2.93   0.003     .5969941    3.005692

Test of residual homogeneity: Q_res = chi2(6) = 23.38   Prob > Q_res = 0.0007
```

Supplementary Fig. 15. Regression analysis plot for high-density lipoprotein cholesterol.

```
meta regress age, random(reml)

  Effect-size label: Hedges's g
        Effect size: _meta_es
          Std. err.: _meta_se

Random-effects meta-regression                    Number of obs  =          8
Method: REML                                      Residual heterogeneity:
                                                              tau2 =   .2409
                                                            I2 (%) =   63.08
                                                                H2 =    2.71
                                                     R-squared (%) =   39.95
                                                  Wald chi2(1)     =    3.84
                                                  Prob > chi2      =  0.0501

    _meta_es   Coefficient  Std. err.      z    P>|z|     [95% conf. interval]

         age      -.040228   .0205317   -1.96   0.050    -.0804694    .0000134
       _cons      .6905934   .7825258    0.88   0.377    -.8431289    2.224316

Test of residual homogeneity: Q_res = chi2(6) = 16.48   Prob > Q_res = 0.0114
```

```
meta regress baselinebmi, random(reml)

  Effect-size label: Hedges's g
        Effect size: _meta_es
          Std. err.: _meta_se

Random-effects meta-regression                    Number of obs  =          8
Method: REML                                      Residual heterogeneity:
                                                              tau2 =   .3518
                                                            I2 (%) =   71.24
                                                                H2 =    3.48
                                                     R-squared (%) =   12.30
                                                  Wald chi2(1)     =    1.49
                                                  Prob > chi2      =  0.2216

    _meta_es   Coefficient  Std. err.      z    P>|z|     [95% conf. interval]

 baselinebmi     -.1182148   .0967141   -1.22   0.222    -.3077709    .0713413
       _cons      2.726544   2.879578    0.95   0.344    -2.917326    8.370414

Test of residual homogeneity: Q_res = chi2(6) = 21.16   Prob > Q_res = 0.0017
```

```
meta regress interventionperiod, random(reml)

  Effect-size label: Hedges's g
        Effect size: _meta_es
          Std. err.: _meta_se

Random-effects meta-regression                    Number of obs  =          8
Method: REML                                      Residual heterogeneity:
                                                              tau2 =   .4643
                                                            I2 (%) =   77.03
                                                                H2 =    4.35
                                                     R-squared (%) =    0.00
                                                  Wald chi2(1)     =    0.16
                                                  Prob > chi2      =  0.6901

          _meta_es   Coefficient  Std. err.      z    P>|z|     [95% conf. interval]

interventionperiod      .0189303   .0474836    0.40   0.690    -.0741358    .1119965
             _cons     -1.002563   .6250372   -1.60   0.109    -2.227614     .222487

Test of residual homogeneity: Q_res = chi2(6) = 27.64   Prob > Q_res = 0.0001
```

Supplementary Fig. 16. Regression analysis plot for low-density lipoprotein cholesterol.

```
meta regress age, random(reml)

  Effect-size label: Hedges's g
        Effect size: _meta_es
          Std. err.: _meta_se

Random-effects meta-regression                      Number of obs  =         9
Method: REML                                        Residual heterogeneity:
                                                                tau2 = 1.3e-07
                                                              I2 (%) =    0.00
                                                                  H2 =    1.00
                                                       R-squared (%) =  100.00
                                                    Wald chi2(1)     =   12.48
                                                    Prob > chi2      =  0.0004

     _meta_es   Coefficient  Std. err.       z    P>|z|     [95% conf. interval]

          age   -.0379202    .010735    -3.53   0.000    -.0589605    -.01688
        _cons    .9709278   .4011741     2.42   0.016     .184641    1.757215

Test of residual homogeneity: Q_res = chi2(7) =   5.01    Prob > Q_res = 0.6589
```

```
meta regress baselinebmi, random(reml)

  Effect-size label: Hedges's g
        Effect size: _meta_es
          Std. err.: _meta_se

Random-effects meta-regression                      Number of obs  =         9
Method: REML                                        Residual heterogeneity:
                                                                tau2 =   .1377
                                                              I2 (%) =   50.11
                                                                  H2 =    2.00
                                                       R-squared (%) =   12.25
                                                    Wald chi2(1)     =    1.28
                                                    Prob > chi2      =  0.2584

     _meta_es   Coefficient  Std. err.       z    P>|z|     [95% conf. interval]

  baselinebmi   -.0708197   .0626626    -1.13   0.258    -.1936362    .0519967
        _cons    1.730275   1.847373     0.94   0.349    -1.89051     5.351059

Test of residual homogeneity: Q_res = chi2(7) = 13.95    Prob > Q_res = 0.0520
```

```
meta regress interventionperiod, random(reml)

  Effect-size label: Hedges's g
        Effect size: _meta_es
          Std. err.: _meta_se

Random-effects meta-regression                      Number of obs  =         9
Method: REML                                        Residual heterogeneity:
                                                                tau2 =   .1972
                                                              I2 (%) =   60.10
                                                                  H2 =    2.51
                                                       R-squared (%) =    0.00
                                                    Wald chi2(1)     =    0.00
                                                    Prob > chi2      =  0.9849

           _meta_es   Coefficient  Std. err.       z    P>|z|     [95% conf. interval]

interventionperiod    .0006747   .0357198     0.02   0.985    -.0693348    .0706843
             _cons   -.350066    .4677674    -0.75   0.454    -1.266873    .5667412

Test of residual homogeneity: Q_res = chi2(7) = 17.41    Prob > Q_res = 0.0149
```

```
meta regress trainingfrequency, random(reml)

  Effect-size label: Hedges's g
        Effect size: _meta_es
          Std. err.: _meta_se

Random-effects meta-regression                      Number of obs  =         9
Method: REML                                        Residual heterogeneity:
                                                                tau2 =   .1203
                                                              I2 (%) =   48.18
                                                                  H2 =    1.93
                                                       R-squared (%) =   23.32
                                                    Wald chi2(1)     =    2.08
                                                    Prob > chi2      =  0.1491

          _meta_es   Coefficient  Std. err.       z    P>|z|     [95% conf. interval]

trainingfrequency     .8125787   .5632335     1.44   0.149    -.2913386    1.916496
            _cons    -2.869846   1.754464    -1.64   0.102    -6.308532    .5688408

Test of residual homogeneity: Q_res = chi2(7) = 13.64    Prob > Q_res = 0.0581
```

Supplementary Fig. 17. Regression analysis plot for total cholesterol.

```
meta regress age, random(reml)

  Effect-size label: Hedges's g
        Effect size: _meta_es
          Std. err.: _meta_se

Random-effects meta-regression                    Number of obs  =          9
Method: REML                                      Residual heterogeneity:
                                                              tau2 =   .02985
                                                            I2 (%) =    18.06
                                                                H2 =     1.22
                                                     R-squared (%) =    90.94
                                                  Wald chi2(1)    =     15.23
                                                  Prob > chi2     =    0.0001

    _meta_es | Coefficient  Std. err.      z    P>|z|     [95% conf. interval]
-------------+----------------------------------------------------------------
         age |  -.0472547   .0121087    -3.90   0.000    -.0709874   -.0235221
       _cons |   1.393815   .4482177     3.11   0.002     .5153245    2.272306
------------------------------------------------------------------------------
Test of residual homogeneity: Q_res = chi2(7) =  8.80    Prob > Q_res = 0.2670
```

```
meta regress baselinebmi, random(reml)

  Effect-size label: Hedges's g
        Effect size: _meta_es
          Std. err.: _meta_se

Random-effects meta-regression                    Number of obs  =          9
Method: REML                                      Residual heterogeneity:
                                                              tau2 =     .304
                                                            I2 (%) =    68.80
                                                                H2 =     3.21
                                                     R-squared (%) =     7.71
                                                  Wald chi2(1)    =      1.30
                                                  Prob > chi2     =    0.2541

    _meta_es | Coefficient  Std. err.      z    P>|z|     [95% conf. interval]
-------------+----------------------------------------------------------------
 baselinebmi |  -.0918317   .0805243    -1.14   0.254    -.2496565    .0659931
       _cons |   2.447667    2.36816     1.03   0.301    -2.193842    7.089175
------------------------------------------------------------------------------
Test of residual homogeneity: Q_res = chi2(7) = 21.54    Prob > Q_res = 0.0031
```

```
meta regress interventionperiod, random(reml)

  Effect-size label: Hedges's g
        Effect size: _meta_es
          Std. err.: _meta_se

Random-effects meta-regression                    Number of obs  =          9
Method: REML                                      Residual heterogeneity:
                                                              tau2 =    .3857
                                                            I2 (%) =    74.42
                                                                H2 =     3.91
                                                     R-squared (%) =     0.00
                                                  Wald chi2(1)    =      0.11
                                                  Prob > chi2     =    0.7419

           _meta_es | Coefficient  Std. err.      z    P>|z|     [95% conf. interval]
--------------------+----------------------------------------------------------------
 interventionperiod |   .0145759   .0442601     0.33   0.742    -.0721723    .1013242
              _cons |  -.4102021   .5775224    -0.71   0.478    -1.542125     .721721
-------------------------------------------------------------------------------------
Test of residual homogeneity: Q_res = chi2(7) = 27.14    Prob > Q_res = 0.0003
```

```
meta regress trainingfrequency, random(reml)

  Effect-size label: Hedges's g
        Effect size: _meta_es
          Std. err.: _meta_se

Random-effects meta-regression                    Number of obs  =          9
Method: REML                                      Residual heterogeneity:
                                                              tau2 =    .3907
                                                            I2 (%) =    74.85
                                                                H2 =     3.98
                                                     R-squared (%) =     0.00
                                                  Wald chi2(1)    =      0.05
                                                  Prob > chi2     =    0.8308

           _meta_es | Coefficient  Std. err.      z    P>|z|     [95% conf. interval]
--------------------+----------------------------------------------------------------
  trainingfrequency |   .1681844   .7868788     0.21   0.831     -1.37407    1.710439
              _cons |  -.7599895   2.457159    -0.31   0.757    -5.575932    4.055953
-------------------------------------------------------------------------------------
Test of residual homogeneity: Q_res = chi2(7) = 27.62    Prob > Q_res = 0.0003
```

Supplementary Fig. 18. Regression analysis plot for triglycerides.

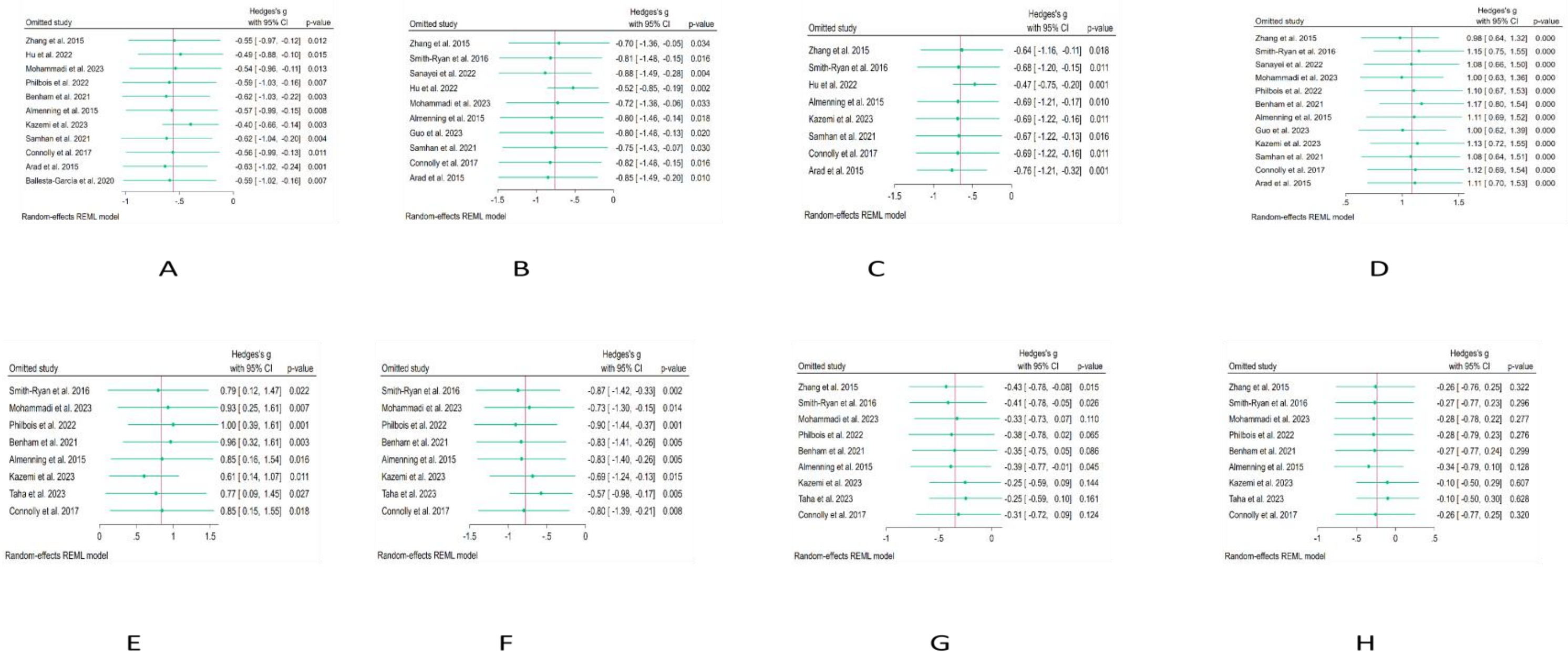

Supplementary Fig. 19A–H. Leave-one-out sensitivity analyses for BMI, BF%, FM, VO_2max/VO_2peak, HDL-C, LDL-C, TC, and TG

Note. Each panel presents the pooled Hedges' g and 95% confidence interval after sequential omission of the indicated study. The vertical reference line represents the pooled estimate based on all eligible studies for the corresponding outcome. All analyses were conducted using the random-effects model, with between-study variance estimated using the restricted maximum likelihood method. Panels represent: (A) body mass index; (B) body fat percentage; (C) fat mass; (D) maximal or peak oxygen uptake; (E) high-density lipoprotein cholesterol; (F) low-density lipoprotein cholesterol; (G) total cholesterol; and (H) triglycerides. BMI, body mass index; BF%, body fat percentage; FM, fat mass; VO_2 max/VO_2 peak, maximal or peak oxygen uptake; HDL-C, high-density lipoprotein cholesterol; LDL-C, low-density lipoprotein cholesterol; TC, total cholesterol; TG, triglycerides; CI, confidence interval.

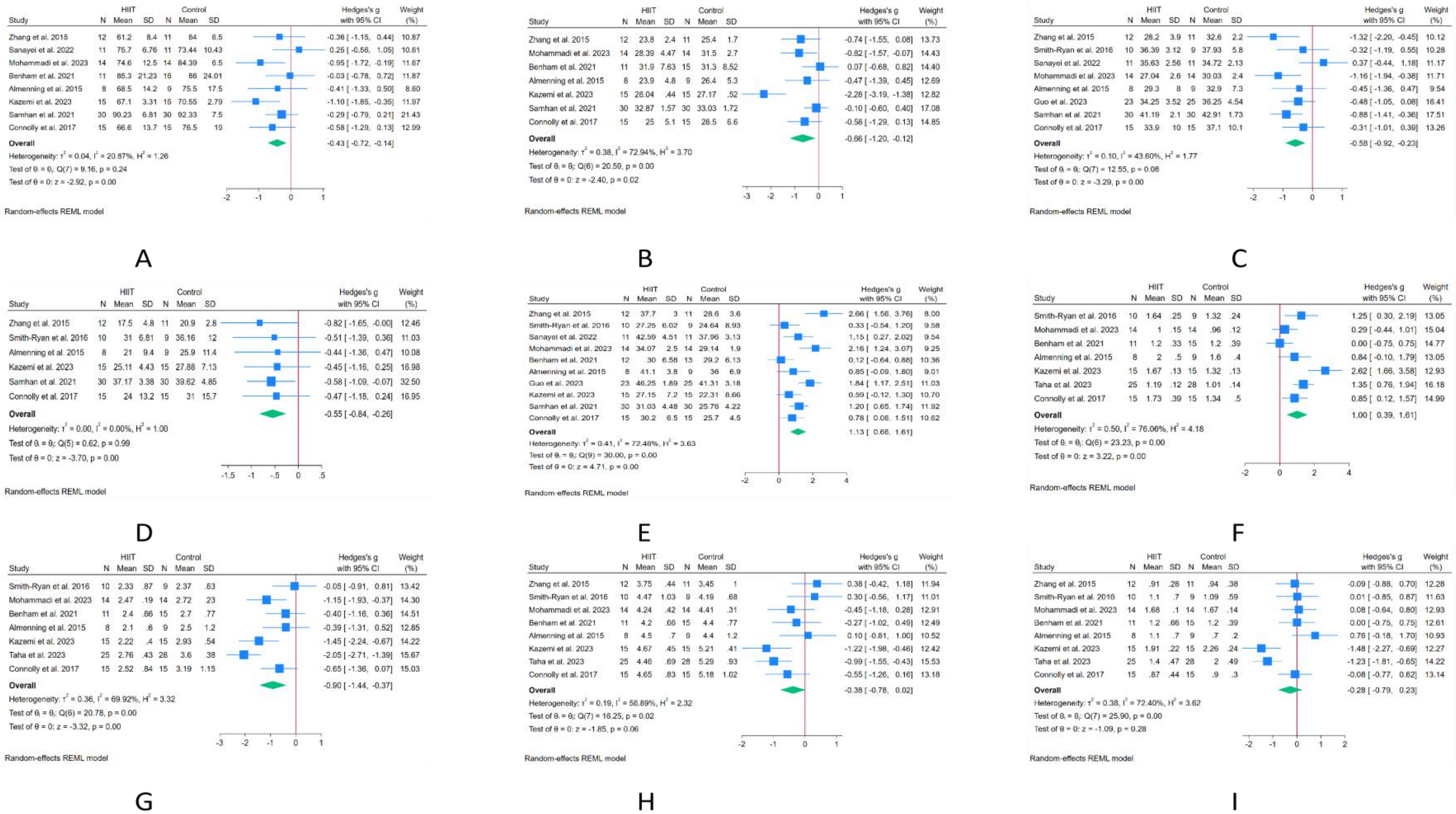

Supplementary Fig. 20A-I. Sensitivity analyses excluding studies judged to be at high risk of bias across the nine outcomes.

Figure note: Each panel shows the pooled Hedges' g and 95% confidence interval after exclusion of studies judged to be at overall high risk of bias according to the RoB 2 assessment. Random-effects models were fitted using the restricted maximum likelihood (REML) method. Negative

Hedges' g values favor HIIT for BW, BMI, BF%, FM, LDL-C, TC, and TG, whereas positive values favor HIIT for VO_2max/VO_2peak and HDL-C. Panels represent: (A) BW; (B) BMI; (C) BF%; (D) FM; (E) VO_2max/VO_2peak; (F) HDL-C; (G) LDL-C; (H) TC; and (I) TG.

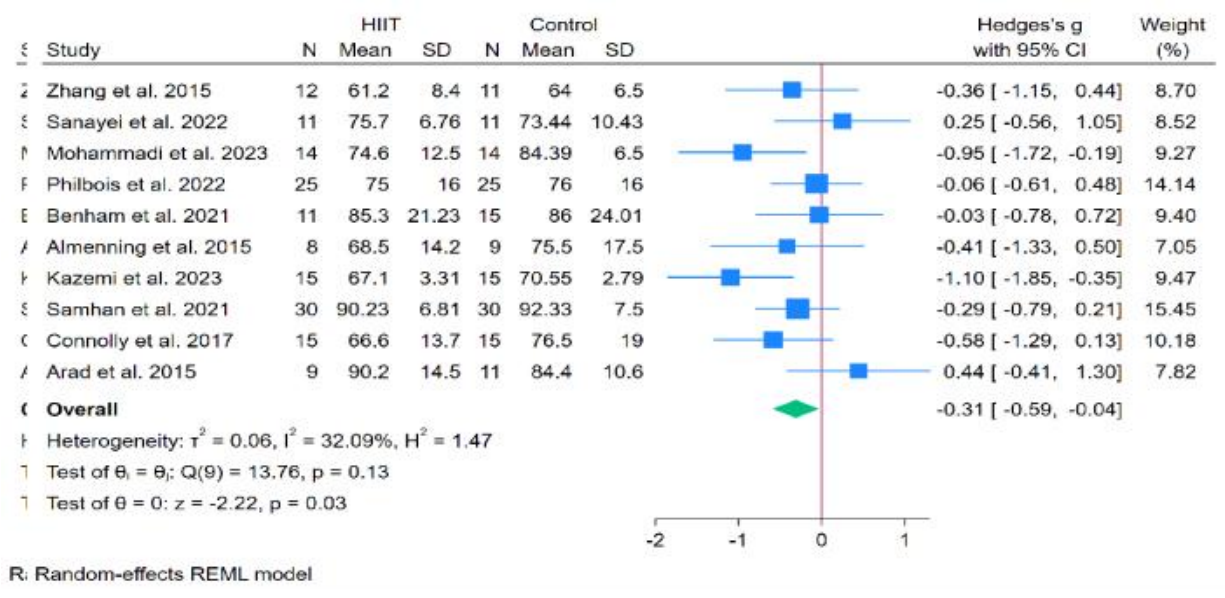

A

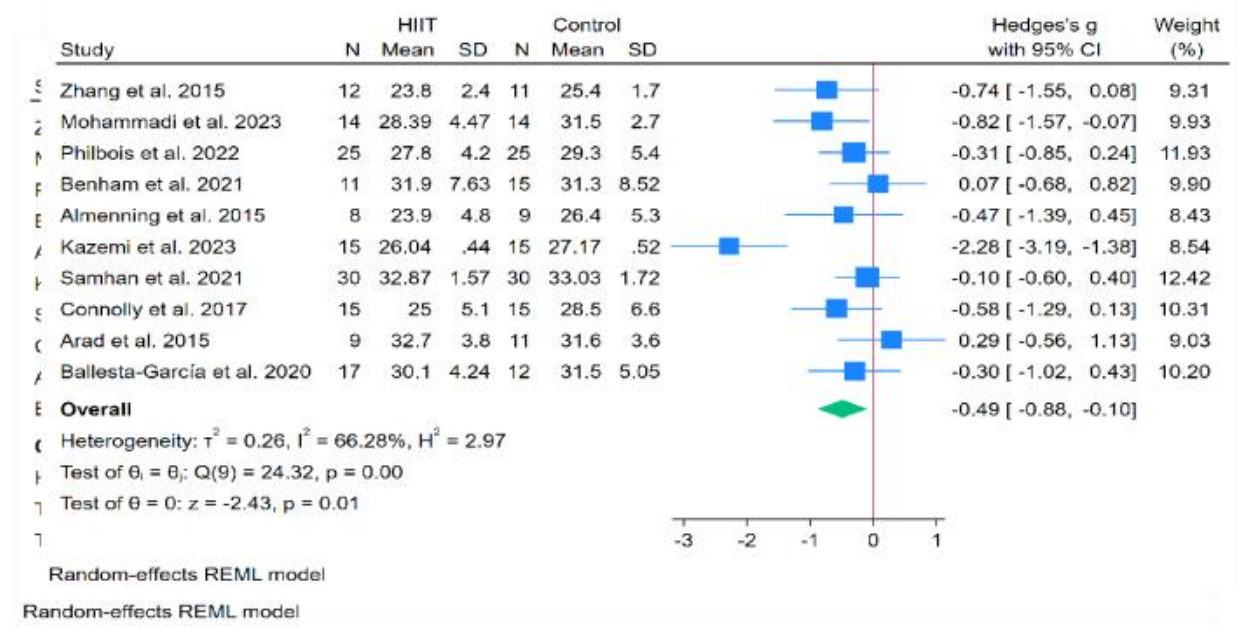

B

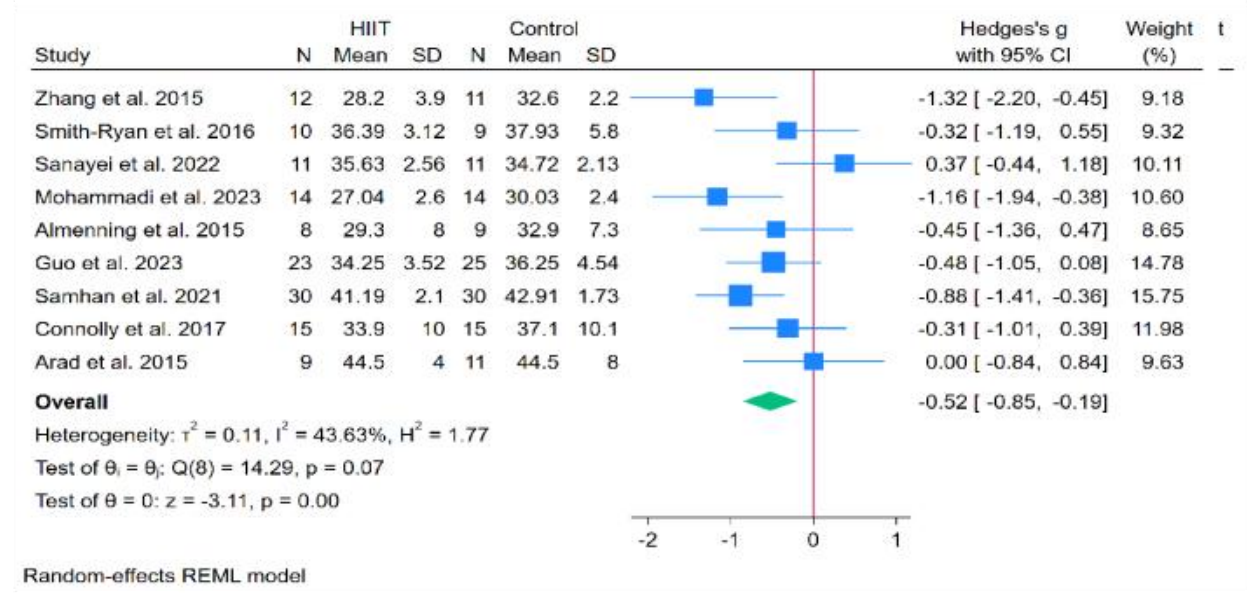

C

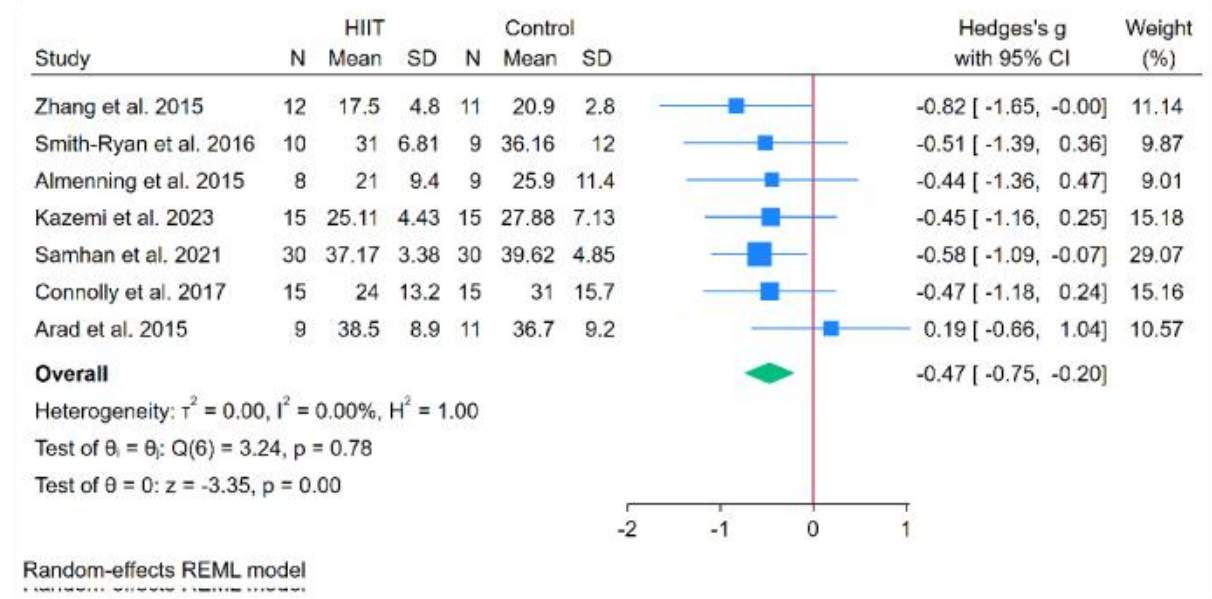

D

Supplementary Fig. 21A–D. Sensitivity analyses excluding Hu et al. (2022) because of unclear population eligibility

Note: Each panel presents the random-effects meta-analysis after excluding Hu et al. (2022), which enrolled women with normal-weight obesity and therefore did not clearly meet conventional BMI-based definitions of overweight or obesity. Panels represent: (A) body weight; (B) body mass index; (C) body fat percentage; and (D) fat mass. Squares indicate study-specific Hedges' g estimates, with square sizes proportional to the corresponding study weights; horizontal lines represent 95% confidence intervals, and diamonds represent the pooled effect estimates. Between-study variance was estimated using the restricted maximum likelihood method. Negative Hedges' g values indicate effects favouring HIIT, corresponding to lower values of the respective body-composition outcomes. These sensitivity analyses were exploratory and should not be interpreted as establishing Hu et al. (2022) as the definitive or sole source of heterogeneity. HIIT, high-intensity interval training; BMI, body mass index; BF%, body fat percentage; FM, fat mass; CI, confidence interval; REML, restricted maximum likelihood.
